# Structure-to-process design framework for developing safer pesticides

**DOI:** 10.1126/sciadv.abn2058

**Published:** 2022-03-30

**Authors:** Jessica M. Lewer, Zachary R. Stickelman, Jessica H. Huang, John F. Peloquin, Jakub Kostal

**Affiliations:** Department of Chemistry, The George Washington University, 800 22nd St NW, Ste 4000, Washington, DC 20052-0066, USA.

## Abstract

Rational design of pesticides with tunable degradation properties and minimal ecotoxicity is among the grand challenges of green chemistry. While computational approaches have gained traction in predictive toxicology, current methods lack the necessary multifaceted approach and design-vectoring tools needed for system-based chemical development. Here, we report a tiered computational framework, which integrates kinetics and thermodynamics of indirect photodegradation with predictions of ecotoxicity and performance, based on cutoff values in mechanistically derived physicochemical properties and electronic parameters. Extensively validated against experimental data and applied to 700 pesticides on the U.S. Environmental Protection Agency’s registry, our simple yet powerful approach can be used to screen existing molecules to identify application-ready candidates with desirable characteristics. By linking structural attributes to process-based outcomes and by quantifying trade-offs in safety, depletion, and performance, our method offers a user-friendly roadmap to rational design of novel pesticides.

## Teaser

System-based computational approach affords design of pesticides with tunable degradation properties and minimal ecotoxicity.

## INTRODUCTION

Green chemistry principles dictate industry should be developing safer chemicals that do not persist in the environment, to lower risk of adverse effects to human and environmental health (*1*). While the concept of safer chemical design was pioneered nearly 100 years ago (*2*), it is estimated that more than 85% of commercial chemicals introduced in the United States annually have insufficient experimental health and safety data (*3*). The U.S. Environmental Protection Agency (EPA) tackles this challenge by using a variety of techniques to fill data gaps to evaluate chemical hazard, exposure, and risk. Nonetheless, the potential threat that these chemicals pose has gained considerable traction in recent years, along with the realization that animal testing methods are not pragmatic by means of speed, economics, or ethics (*3*–*5*). In our attempts to mitigate this threat, in vitro and in silico models, collectively referred to as New Approach Methodologies, have been promoted to inform hazard and risk assessments (*4*). These methods have successfully addressed many industry needs, such as streamlining toxicity testing, down-selecting compounds in preclinical settings, predicting drug rankings, or elucidating chemical bioaccumulation (*4*).

In developing new chemicals, drug discovery is a prime example of a sector that uses systems thinking (*6*), evaluating a multitude of factors such as potency, selectivity, and human, as well as environmental safety in their approach. This type of holistic design methodology has increasingly relied on computational modeling to alleviate costs and reduce time to market (*7*). In principle, a similar approach can be devised to inform design of commodity chemicals, such as cosmetics, cleaners, or pesticides (*3*). In our previous work, we have proposed a framework, akin to the drug discovery processes, for the design of safer chemicals, which incorporates drivers of toxicity, metabolism, and functionality, using a proof-of-concept model based on organophosphate flame retardants (*5*). We have also postulated that in translating methods from drug discovery to commodity-chemical design, one must be cognizant of key sector differences (*8*), which can not only yield opportunities (e.g., most bulk chemicals are not designed to be biologically active) but also pose challenges (e.g., the pharmaceutical industry enjoys cost-benefit ratios in new product development vastly different from other chemical manufacturers).

Pesticides are a unique class of commodity chemicals in that they are, such as pharmaceuticals, intended to be biologically active or, more specifically, to exhibit selective toxicity. From a risk assessment standpoint, this is problematic, as the use of pesticides continues to rise with increasing crop demand (up to 3.5 million metric tons globally in 2020) (*9*), while the global cost-benefit ratio has declined due to persistence and associated negative environmental effects (*10*). Because pesticides’ risk to humans and the environment is a function of both exposure (driven by persistence) and hazard (driven by intrinsic toxicity) (*11*, *12*), elucidating the underlying structural drivers is necessary in developing safer alternatives.

While chemicals can be broken down by a host of biotic and abiotic processes, photolysis is our first line of defense against pesticides. Specifically, reactions with photochemically produced reactive intermediates (PPRIs) represent the most ubiquitous abiotic degradation pathway undertaken by agrochemicals after use (*13*–*15*). Zeng and Arnold (*16*) showed that among the various PPRI compounds, oxidation via excited triplet state chromophoric dissolved organic matter (^3^CDOM^*^) is the most prolific. ^3^CDOM^*^ accounts for as much as 80% of pesticide degradation due to a variety of sensitizers present in the environment, with reduction potentials ranging from 0.15 to 2.38 V (*16*, *17*). Crucially, this range encompasses reduction potentials of other PPRI molecules, including singlet oxygen (0.65 V), hydroxyl radical (2.33 V), superoxide (0.94 V), and hydrogen peroxide (0.32 V) (fig. S1) (*16*). Thus, ^3^CDOM^*^ is an effective proxy system for all PPRI electron-transfer reactions and, by extension, the majority of abiotic degradation processes.

Failing to control pesticides’ degradation exposes us to the hazards that these chemicals pose to living systems, which can lead to devastating consequences, as observed throughout history for chemicals such as DDT, bisphenol A, and paraquat (*5*, *9*, *18*). In contrast to other chemical classes, the intended selective toxicity of pesticides that defines their function spells trouble for species we intend to keep away from harm (*9*). Without adequate toxicology screening of each new compound, these effects can translate to nontarget organisms and may be found long after the chemical has been commercialized (*9*, *18*). To that end, the amended FIFRA (Federal Insecticide, Fungicide, Rodenticide) Act requires extensive testing of new pesticides before approval for registration and use. Perturbations to the health of aquatic ecosystems—such as microorganisms, invertebrates, plants, and fish—are often the first marker of a chemical’s overuse in the environment (*19*). This is especially relevant for agrochemicals, which leach into surface waters as runoff after use (*19*).

**Fig. 1.**
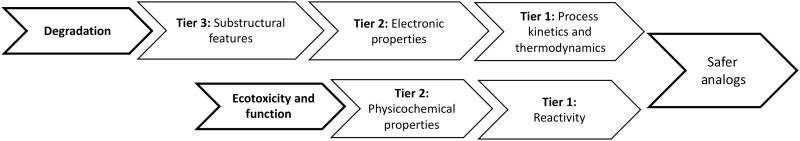
Design framework. Structure-to-process framework for the design of safer pesticides based on computed photodegradation (*17*) and ecotoxicity rules (*21*).

Here, we outline a comprehensive in silico strategy for screening existing and designing new, safer pesticides, which represents a nexus of our past efforts in computational chemistry, green chemistry, and toxicology. We rely on acute aquatic toxicity as the primary end point for ecotoxicity assessments, recognizing its applicability in informing chronic toxicity (*20*–*22*) and in predicting toxic effects in other species by encompassing a wide range of modes and mechanisms of action (*21*, *23*–*25*). In our model, we leverage previously developed and validated design guidelines (*21*, *22*, *26*), which balance mechanistic relevance with nonspecific reactivity, thus serving as a useful proxy for a general toxic potential and a descriptor of pesticides’ intended (biological) function. Photodegradation is considered using models for pesticide-^3^CDOM^*^ interactions (*17*), which were augmented by computational analyses that link pesticide structures to process metrics (i.e., degradation, toxicity, and function) (*17*). The proposed framework was applied to pesticides on the EPA’s registry with the goal to formulate a blueprint for robust molecular design. Figure 1 illustrates the integrated design tiers, which guide the end user from substructural features to structural properties and, lastly, to process metrics, focusing on molecular perturbations that optimize the trade-offs in designing novel analogs. We envision this framework to support upstream decision-making in new product development and to translate to other chemical classes and industry sectors in the pursuit of green chemistry principles and a more sustainable chemical design.

## RESULTS

### Integrated photodegradation-ecotoxicity analysis

**Fig. 2.**
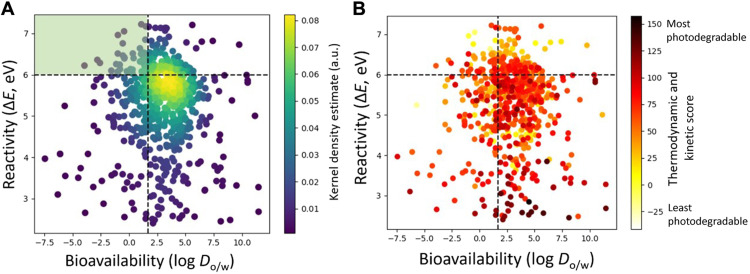
Density scatterplots. (**A**) Left: Density scatterplot of octanol-water distribution coefficient (log *D*_o/w_) versus energy difference between the highest occupied and lowest unoccupied molecular orbitals (Δ*E*). Safer chemical space defined by the current method (mPW1PW91/MIDIX+) is highlighted in the upper left-hand quadrant (log *D*_o/w_ < 1.7 and Δ*E* > 6 eV). The average ecotoxicity point for all 700 compounds was found to be a log *D*_o/w_ of 2.87 and a Δ*E* of 5.29 eV, with SDs of 2.56 and 0.99, respectively. (**B**) Right: Indirect photodegradation potential is represented by the combined thermodynamic and kinetic performance, where the darker the point, the more likely the molecule is to photodegrade. The average Δ*G*_et_^0^ and log *k* values for all 700 compounds were found to be 10.68 kcal/mol and 8.65, with SDs of 10.83 and 0.95, respectively. a.u., arbitrary units.

In our previous work, we showed that cutoff values in the octanol-water distribution coefficient (log *D*_o/w_) and the energy difference between the highest occupied and the lowest unoccupied molecular orbitals (Δ*E*) can be used to identify compounds with high probability of minimal acute and chronic ecotoxicity (*21*, *22*, *26*). Here, we performed this analysis across 700 pesticides with PPRI-oxidizable cores, obtained from the EPA’s CompTox Chemical Dashboard (see Materials and Methods and table S1, columns 2, 3, and 8). We noted that only 52 compounds analyzed (ca. 7% of the dataset) fulfilled the criteria of the “safer chemical space” (log *D*_o/w_ < 1.7 and Δ*E* > 6 eV in Fig. 2, shaded in green). A full list of pesticides that did meet our criteria for safety can be found in table S2. From Fig. 2A, the highest density of EPA pesticide data is just above the threshold for safer log *D*_o/w_ values (>1.7) and fluctuates around the cutoff for Δ*E* (6 eV). These results suggest that while most pesticides are likely not safe, they could be “made safe(r)” by perturbations of their molecular structure. Ideally, such a feat is accomplished by changes that satisfy both Δ*E* and log *D*_o/w_ cutoff values. However, bioavailability (log *D*_o/w_), which is MOA independent, is expected to play a more substantial role simply because if a compound is not bioavailable, then its reactivity is less important. We cannot state the same about Δ*E*, as nonreactive chemicals can be metabolized into potent toxicants.

We should note here that falling outside the safer space does not directly imply toxicity but rather that the probability of safety has decreased. For example, our analysis of fathead minnow acute toxicity data showed that ca. 50% of low-concern and 10% of no-concern chemicals were found outside the “safe space” (*21*). Nonetheless, the results here are troubling, yet consistent with past reports (*27*), in that most pesticides pose nontrivial hazard to environmental health. Fortunately, hazards can be, in part, alleviated by depletion, where compounds that easily degrade (into benign by-products) may be of lesser concern due to their lower bioaccumulation. To probe for a potential relationship between depletion and safety (both rely on chemical reactivity), we augmented the above analysis with pesticides’ average photodegradation potential across representative ^3^CDOM^*^, mimicking mixture-like effects in nature (*17*). In Fig. 2B, photodegradation propensity was expressed using a composite thermodynamic and kinetic score, generated by combining percentage values of predicted rate constants (log *k*’s) and free energies of pesticide-to-^3^CDOM^*^ electron transfer (Δ*G*_et_^0^’s) for each compound (viz. Materials and Methods). Since drivers of depletion and toxicity are not entirely independent, while a majority of compounds analyzed here (649) falls outside the safe space, 51% of those compounds also have photodegradation rates above the 50th percentile, with 100 compounds falling between the 75th and 89th percentile and 69 compounds above the 90th percentile (Fig. 2B).

**Fig. 3.**
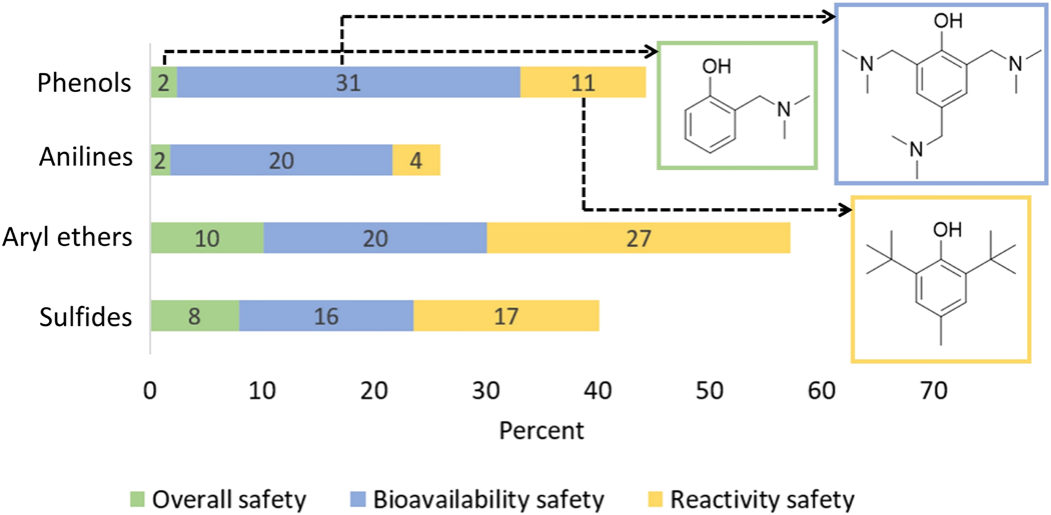
Safety assessment by pesticide class. Percent (%) breakdown of pesticides by safety criteria and by pesticide chemical class. Green, blue, and yellow bars represent % of compounds that met the overall safety cutoffs (log *D*_o/w_ < 1.7 and Δ*E* > 6 eV), predicted the bioavailability safety (log *D*_o/w_ < 1.7), and predicted the reactivity safety (Δ*E* > 6 eV), respectively.

From a design perspective, it is important to understand the relative contributions of the various pesticides’ classes to outcomes presented in Fig. 2. Focusing on phenols and anilines (fig. S2), we found that both classes are more reactive than the rest of the dataset (Δ*E* = 4.81 eV and SD = 1.09 eV for phenols and Δ*E* = 4.80 eV and SD = 1.13 eV for anilines). While they are not markedly different in terms of bioavailability, the spread in log *D*_o/w_ values for phenols was greater than any other class (fig. S2), presenting an intriguing opportunity for design, as greater log *D*_o/w_ range does not appear to inhibit function. In noting specific examples, 2-dimethylaminomethyl phenol and asulam (fig. S3) demonstrated the best-optimized trade-offs across all three parameters (photodegradation, log *D*_o/w_, and Δ*E*) for their respective chemical class. We posit that the electron-donating amine group on 2-dimethylaminomethyl phenol contributes to favorable photodegradation and, as a hydrogen-bond acceptor, decreases log *D*_o/w_. Similarly, in elucidating structure-activity relationship for asulam, hydrophilic sulfonamide and ester groups decrease log *D*_o/w_, but the competing electronic nature of sulfonamide (electron-withdrawing) and amine (electron-donating by resonance) leads to lower photodegradation rates. In general, anilines were found to be less likely to perform well across all parameters, as photodegradation is tied to Δ*E* more so than for phenols (fig. S3), suggesting that redesigning compounds may be easier for phenols versus anilines. Exploring this further, we examined the percent breakdown of safety criteria met, per pesticide chemical class, across all 700 compounds (Fig. 3), which confirmed that anilines are the least likely to meet our safety criteria, while aryl ethers are the most likely. Both anilines and phenols are reactive nucleophiles (a trait that makes them photodegradable); however, as nitrogen is more likely to give up electrons than oxygen, anilines edge phenols in reactivity, which, in turn, makes them least likely to meet the Δ*E* cutoff. Since most toxicants are electrophiles (*28*–*30*), we also compared the lowest unoccupied molecular orbital (LUMO) energies across the dataset and found that anilines had the lowest average (−1.92 eV) as compared to phenols (−1.89 eV), aryl ethers (−1.44 eV), and sulfides (−1.21 eV).

**Fig. 4.**
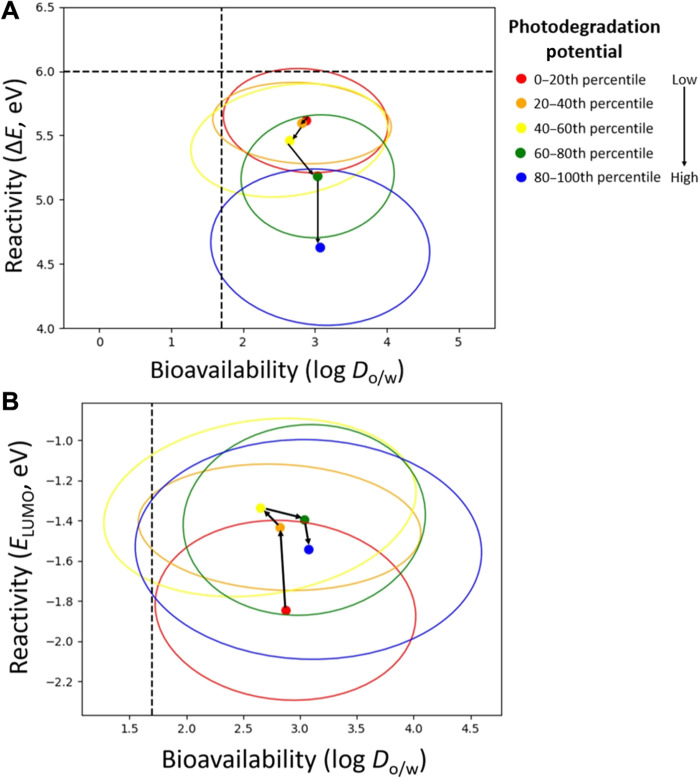
Coupling of ecotoxicity and photodegradation. (**A**) Top: Scatterplots of ecotoxicity averages (octanol-water distribution coefficient, log *D*_o/w_ versus energy difference between the highest occupied and lowest unoccupied molecular orbitals, Δ*E*) and spread (ellipse radii based on half SD in the *x* and *y* directions) for each percentile bracket of photodegradation potential (denoted in the legend on the right). Black arrows represent vectors between adjacent percentile averages. (**B**) Bottom: Scatterplot of electrophilic-specific ecotoxicity averages (octanol-water distribution coefficient, log *D*_o/w_ versus energy of the LUMO, *E*_LUMO_).

Trade-offs are intrinsic in chemical design, and while they cannot be avoided, they should be optimized. Above, we noted the “cross-talk” between photodegradation and ecotoxicity metrics, which both rely on FMO (frontier molecular orbital) parameters (viz. Materials and Methods). To probe this relationship, we constructed density scatterplots, which “track” key percentile ranges of photodegradation propensity across the ecotoxicity-defined chemical space (Fig. 4). From Fig. 4A, there is a correlation between increasing photodegradation potential (Δ*E*_HOMO-SOMO_) and average reactivity in aquatic species (Δ*E*_HOMO-LUMO_), as indicated by vertical shift along the Δ*E* axis. This trend stems from the highest occupied molecular orbital (HOMO) energy and is the most evident at the highest level of photodegradation, i.e., in the shift from 60th to 80th percentile to 80th to 100th percentile category. While faster degradation may partly alleviate toxicity concerns, the goal, in concordance with green chemistry principles, is to reduce hazard and optimize depletion. To that end, the trend between photodegradation and ecotoxicity can be “decoupled” as HOMO energy is indicative of nucleophilic reactivity and thus the main driver of pesticide oxidation, while LUMO energy drives safety, as most toxicants are electrophiles (viz. fig. S4) (*28*–*30*). Substituting *E*_LUMO_ for Δ*E* in Fig. 4B (*26*) does just that, creating a design space for pesticides that perform well across both categories of degradation and ecotoxicity. This analysis was replicated for individual pesticide classes (phenols and anilines in fig. S5) with similar results, attenuated by changes in bioavailability (Δlog *D*_o/w_).

### Substructural analysis

**Fig. 5.**
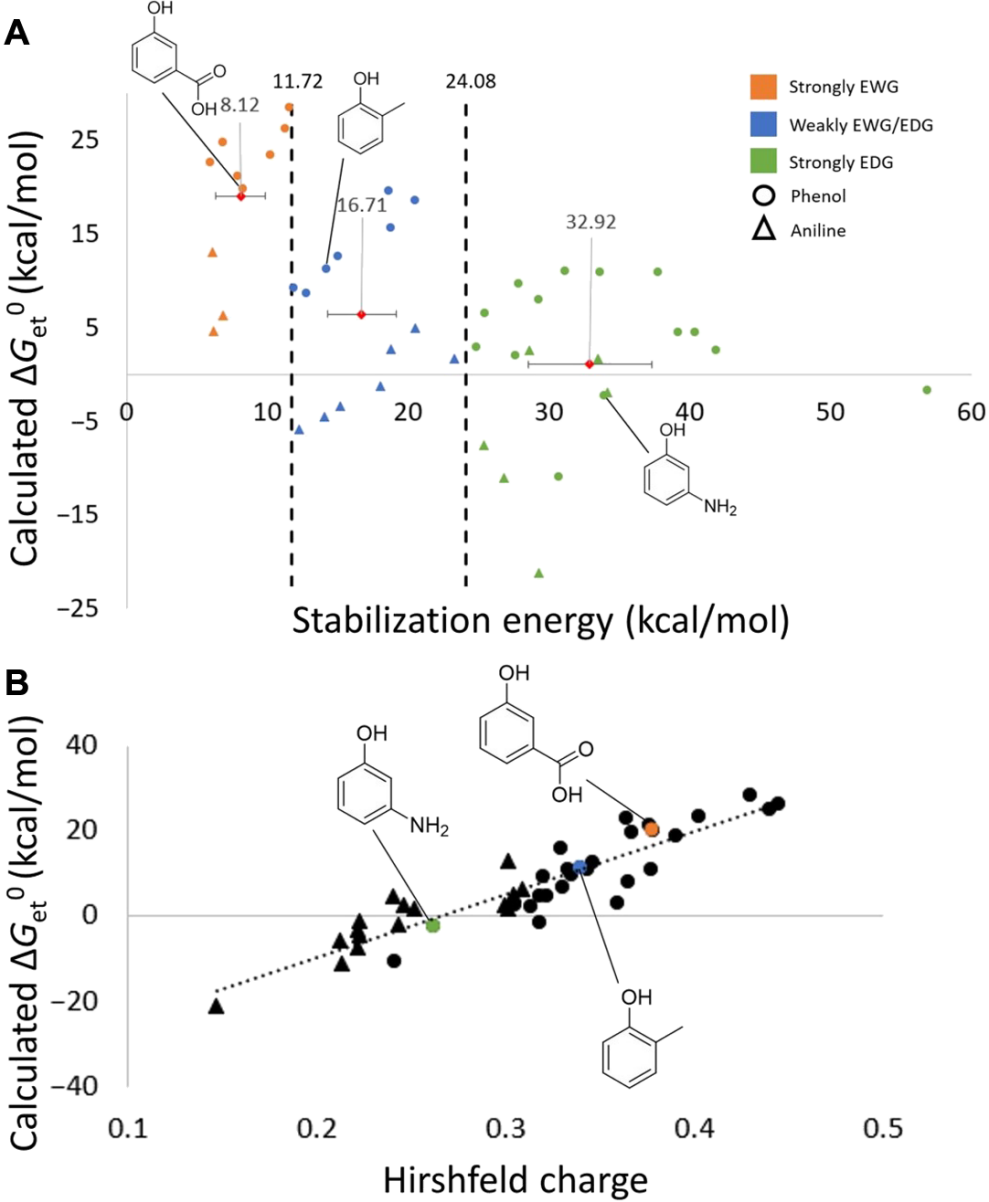
Substructural-tier results. (**A**) Top: Free energy of electron transfer with 3-methoxyacetophenone (Δ*G*_et_^0^) (*17*), plotted as a function of stabilization energy, *E*(2). Cutoff values were identified as follows: (i) strongly electron withdrawing (orange), (ii) weakly electron donating or withdrawing (blue), and (iii) strongly electron donating (green). Averages for each group are designated by a red diamond with *E*(2) value labeled (in kcal/mol). Horizontal bars represent a 99% confidence interval for each group. Vertical dashed lines signify cutoff values that separate groups 1 to 3. Phenols are marked as circular data points, and anilines are denoted by triangles. EWG, electron-withdrawing group; EDG, electron-donating group. (**B**) Bottom: A univariate correlation between a total Hirshfeld charge on the aromatic ring (∑*_r_X_C_*) and Δ*G*_et_^0^. *R*^2^ = 0.83, Δ*G*_et_^0^ = 146.62 × ∑*_r_X_C_* − 38.89, *P* = 2.68 × 10^−17^, root mean square error = 4.50, and *n* = 43. Phenols are marked as circular data points, and anilines are denoted by triangles.

While pesticides’ electronic structure can support design based on established principles and chemist’s own intuition, analysis of substructural properties can directly guide molecular perturbations necessary to achieve desired outcomes. In photodegradation, the rate-determining step, i.e., the electron transfer from pesticide to ^3^CDOM^*^, is driven by the stability of the resulting radical cation intermediate. In phenols and anilines, the electron hole is stabilized by inductive and resonance effects involving ring atoms and substituents, which can withdraw electron density (= destabilizing) or donate it (= stabilizing). We computed these effects using second-order perturbation theory in the natural bond orbital (NBO) basis and by Hirschfeld population analysis (HPA) to support quantitative trade-off assessment in the design process (Fig. 5).

From Fig. 5A, pesticides were grouped on the basis of substituents’ ability to stabilize the electron hole, leading to a statistically significant relationship between stabilization energy, *E*(2), and Δ*G*_et_^0^. A 99% confidence interval for *E*(2) values was created for each group, 6.4 to 9.9 kcal/mol (strongly electron-withdrawing), 14.3 to 19.2 kcal/mol (weakly withdrawing/donating), and 28.5 to 37.3 kcal/mol (strongly electron-donating). We identified a cutoff value, where stabilization energies greater than 24 kcal/mol corresponded to pesticides with good propensity to photodegrade. Alternatively, one can leverage computed electron density on the pesticide core (in the form of partial atomic charges) as both an accessible and a notably predictive approach for gauging (sub)structure-activity relationships (Fig. 5B). From Fig. 5B, we observed a strong linear correlation [*R*^2^ (coefficient of determination) = 0.83] with Δ*G*_et_^0^ values, indicating that more electron density on the pesticide core corresponds to more facile photodegradation. In designing new pesticides, table S1 can guide relevant substituent read-across, and our method serves as a quick tool to assess additional structures.

## DISCUSSION

### Screening pesticides for desirable properties

**Fig. 6.**
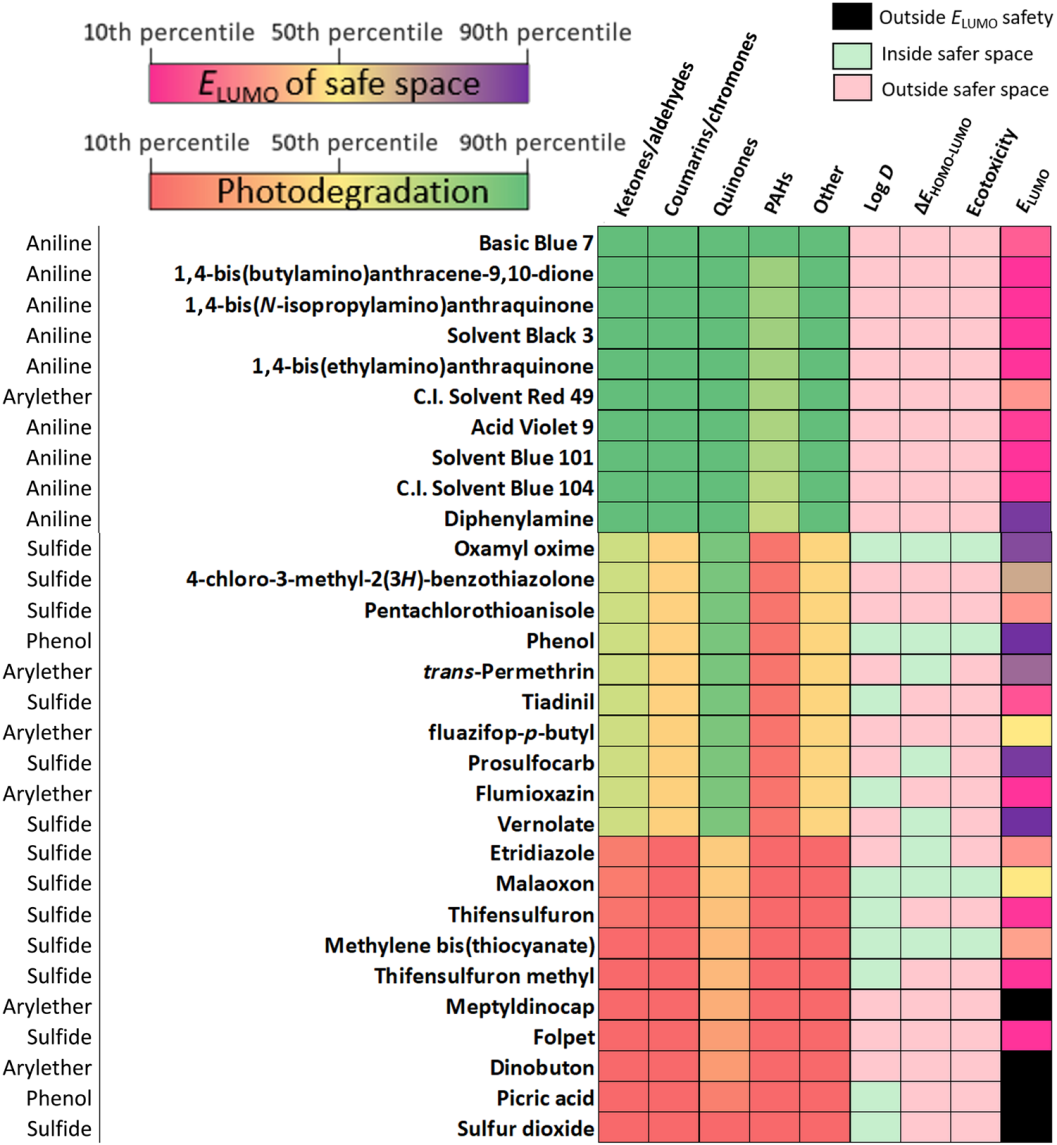
Environmental performance of pesticides. Combined photodegradation and ecotoxicity analysis for a subset of 30 pesticides representing the top, middle, and bottom 10 performers in depletion. Photodegradation: Red indicates higher Δ*G*_et_^0^ values and slower reaction rates, and green indicates lower Δ*G*_et_^0^ values and faster reaction rates. Ecotoxicity: Light green indicates within safer chemical space, and light red indicates outside safer chemical space. *E*_LUMO_ (percentile of *E*_LUMO_ distribution of no- to low-concern chemicals); purple indicates higher *E*_LUMO_ values and increased safety, pink indicates lower *E*_LUMO_ values, and black indicates outside *E*_LUMO_ safety.

In the simplest sense, our models can be used either to select suitable pesticides that balance photodegradation and ecotoxicity outcomes or to perform read-across analysis for pesticides not in our dataset. To that end, we constructed heatmaps that offer “semaphore” coding for each design criterion based on a combined analysis of all 16,100 pesticide interactions with a ^3^CDOM* mixture (viz. fig. S6, with representative subsets of top, middle, and bottom 10 performers in Fig. 6). Because of the coupling of Δ*E*_HOMO-SOMO_ and Δ*E*_HOMO-LUMO_, we encourage the application of *E*_LUMO_ as the more useful driver of ecotoxicity in this analysis.

### Guiding design of safer pesticides

The use of in silico design tools outside drug discovery is still rare due to the challenge of robustly relating structural attributes of chemicals to mechanistic events, especially with limited experimental data (*8*). Modern statistical solutions and adaptive-learning algorithms require big data and means of generating useful analogs to propose new chemicals with desirable properties. Here, and in our previous reports (*17*, *31*), we have taken a different approach, which rests on the premise that design of safer chemicals should be transparent and rational, i.e., rooted in fundamental axioms that yield predictable outcomes. It is for the sake of transparency and practical utility that we developed our structure-to-process design protocol as a tiered computational framework (Fig. 1). In Lewer *et al.* (*17*), we showed that computed process energetics can yield robust models predictive of indirect photodegradation (tier 1) while facilitating expansion of the training set, which can subsequently support structure-based models that rely on pesticides’ electronic properties (tier 2). To enable the design of novel compounds, we incorporated tier 3 into our framework, which relates basic properties of pesticide substructures to tier 2 and tier 1. These moieties can be systematically altered by the chemist to guide a compound toward more favorable process metrics.

**Fig. 7.**
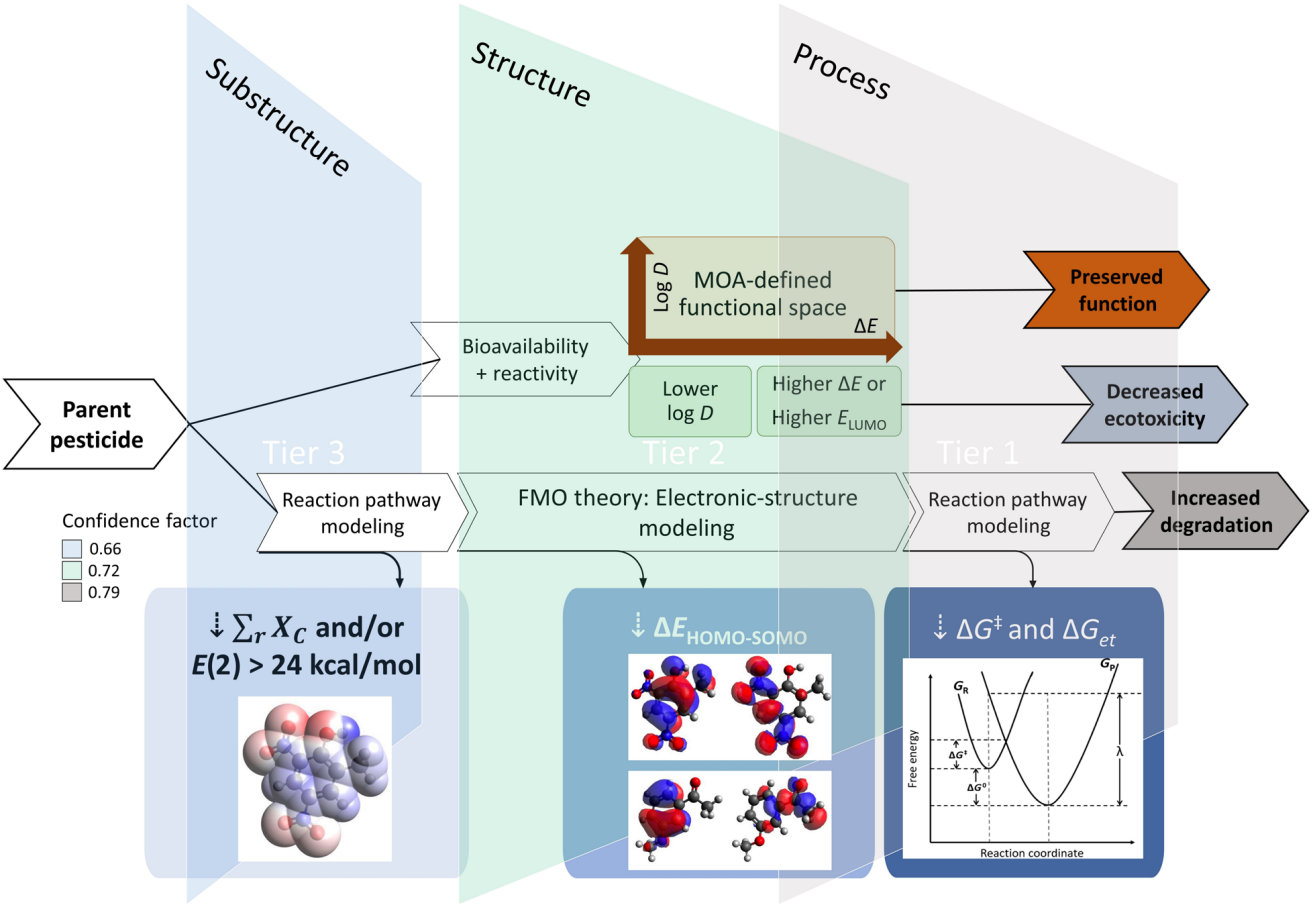
Rational design strategy. Design framework for safer pesticides with controlled degradation.

Figure 7 outlines our proposed blueprint for structure-to-process design of novel pesticides. Here, we offer three “design exits,” which users can take based on their level of expertise and desired level of accuracy given the type of models developed for tiers 1 to 3 (viz. Materials and Methods). Relative confidence scores are provided on the basis of model performance to help assess trade-offs in choosing a specific approach. For compounds with aromatic cores, the user starts by analyzing the stability of the oxidized pesticide, making synthetically feasible substitutions and assessing their impact via substructural models (i.e., HPA and NBO analyses). Promising candidates can be validated in structural models, where photodegradation thermodynamics and kinetics are accurately predicted from FMO energies; relative and absolute (eco)toxicity are gauged from log *D*_o/w_, Δ*E*, and/or *E*_LUMO_ distribution of low- to no-concern chemicals; and performance requirements are tested by defining chemical and functional class boundaries as well as cutoff values for properties correlative to the toxicity thresholds of the pesticide’s MOA. Here, performance limits were identified using the current dataset and commercial chemicals modeled for acute and chronic aquatic toxicity in terms of log *D*_o/w_ and Δ*E* in our previous studies (*21*, *22*, *26*), where both parameters showed significant correlations with toxicity thresholds across all MOAs (Table 1). Following structural tiers, the user can proceed to further validate outcomes by reaction pathway modeling, computing Δ*G*_et_^0^ and *k*_et_ values according to developed models (*17*). In its entirety, the above protocol offers a robust means for analyzing and scoring pesticide analogs for their safety, function, and persistence, resulting in the design of a new compound with a preferable profile.

**Table 1.** Boundary values. Performance boundary conditions in terms of log *D*_o/w_ and Δ*E* across pesticides’ functional class and mode of action (MOA). Functional class cutoffs were determined using pesticide active ingredients. For MOA-based definitions, only commercial chemicals active in ecotoxicity assays were used. UOP, uncoupling of oxidative phosphorylation; ACE, acetylcholinesterase; N, narcosis; CNS, central nervous system seizure or stimulant; EP, electrophile or pro-electrophile reactivity; NDPs, neurodepressants; PN, polar narcosis.

	**Category**	**Log *D*_o/w_** **bounds**	**Δ*E* bounds** **(eV)**
Functional class	Acaricide	−0.55–8.1	3.75–6.76
	Fungicide	−6.1–6.34	2.51–7.13
	Herbicide	−1.53–6.61	3.43–7.14
	Insecticide	−7.44–7.19	3.97–6.96
	Undefined	−5.69–11.47	2.40–7.17
Mode of action	UOP	−0.37–4.22	3.96–5.43
	ACE	0.32–4.89	4.35–6.51
	N	−3.42–5.74	3.71–10.08
	CNS	−0.94–6.77	5.39–6.61
	EP	−0.88–5.18	3.62–9.41
	NDP	−2.12–2.1	4.54–6.43
	PN	−5.44–5.74	3.45–7.71

### Examples of pesticide (re)design

While the above protocol can be applied to any pesticide, the specific strategy of redesigning an existing structure is informed by the attribute (ecotoxicity or persistence) that needs improving. From substructural-tier models, we know that adding electron-donating groups stabilizes the oxidized pesticide, promoting electron transfer to PPRIs and degradation. To drive ecotoxicity metrics toward greater probability of safety, we can alter electronic distribution in a molecule by strategically placing certain substituents on oxidizable cores, affecting Δ*E* and *E*_LUMO_ values. In addition, structures can be made less lipophilic (by decreasing log *D*_o/w_) to lower bioavailability. We should note that in designing new pesticides (versus commercial chemicals without selective toxicity), the balance between hydrophilicity and lipophilicity is important. Agrochemicals may be sprayed as an aqueous solution (and so must be hydrophilic), but to limit leaching into waterways and to ensure uptake by a plant through soil/foliage (or ingestion by pest), they must also be sufficiently lipophilic. The latter is imperative to retain efficacy at low application rates. To that end, the “Briggs Rule of 3,” i.e., log *D*_o/w_ < 3, is often used for pesticides’ active ingredients to strike balance between efficacy and safety (*32*). Thus, in addition to our MOA-defined performance limits, we explored both log *D*_o/w_ cutoffs (1.7 and 3) below.

**Fig. 8.**
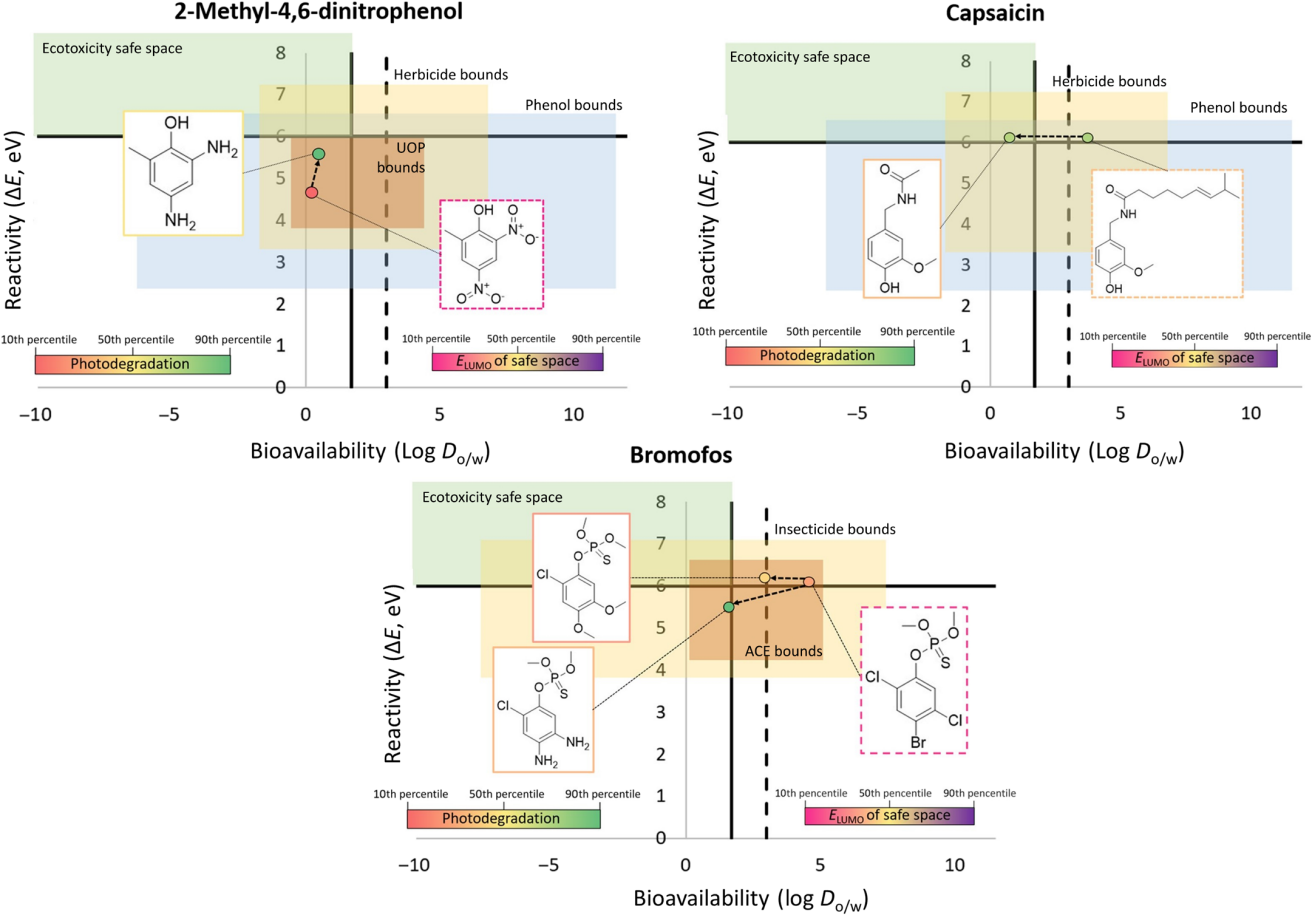
Redesigned pesticides. Design charts based on protocol outlined in Figure 7 for three exemplary pesticides: DNP (top left), capsaicin (top right), and bromofos (bottom). According to the provided scales, the color of the structures’ outline marks the percentile of *E*_LUMO_ distribution of no- to low-concern chemicals; the color of the dot represents percentile of photodegradation (green = highly photodegradable; red = nondegradable), and the position of the dot represents relative safety in terms of log *D*_o/w_ and Δ*E* and the fit in the class- and MOA-defined functional space. Dashed vertical line represents the log *D*_o/w_ < 3 limit, i.e., Briggs Rule of 3.

In our examples, we demonstrate how these generalized rules can be applied in practice to redesign 2-methyl-4,6-dinitrophenol (DNP), capsaicin, and bromofos (Fig. 8), which were selected on the basis of their strong performance in one category (photodegradation or ecotoxicity) and weak performance in the other while showcasing a variety of MOAs. From Fig. 8, DNP is an herbicide that acts by uncoupling oxidative phosphorylation (UOP). While DNP satisfies our log *D*_o/w_ cutoff (as well as Briggs Rule of 3), Δ*E* is below 6 eV and photodegradation performance is in the bottom 10th percentile. Replacing nitro groups with electron-donating amines increased photodegradation potential to the 90th percentile (Fig. 8) while raising Δ*E* by 0.9 eV and increasing *E*_LUMO_ from <1st to the 50th percentile of low- to no-concern chemicals. At the same time, the new analog remains within performance bounds for herbicides (functional class), phenols (chemical class), and active UOP (MOA class) chemicals.

In a reverse scenario, capsaicin is readily photodegradable but is of concern for ecotoxicity due to log *D*_o/w_ > 1.7. Capsaicin is an animal repellent, often used against insects and mites. It has a target-specific mode of action, binding a transient receptor potential cation channel subfamily V member 1 (TRPV1), a nonselective cation channel (*33*), which leads to depolarization of nociceptive neurons (*34*). To decrease the bioavailability of capsaicin, the hydrocarbon chain was truncated, resulting in a decrease in log *D*_o/w_ from 3.75 to 0.75, shifting the redesigned compound into the safer space. Alternatively, a subtler structural change is possible to satisfy log *D*_o/w_ < 3 to ensure better crossing of membranes in pests (viz. Briggs Rule of 3). In terms of performance, capsaicin must also retain its ability to bind TRPV1, which is dominated by a hydrogen bond between amide nitrogen of capsaicin and a tyrosine hydroxyl group in the vanillyl pocket (*33*). Since this part of the molecule was left unperturbed, it is reasonable to propose that desired activity was affected minimally; however, further validation can be carried out using target-specific modeling tools (*5*).

Bromofos is an insecticide acetylcholinesterase inhibitor, which was found to perform poorly in terms of both photodegradation and ecotoxicity. In our redesign effort, we substituted (persistent) halogens with electron-donating and hydrophilic amine and methoxy groups to both increase photodegradation and decrease bioavailability. Substitutions with methoxy groups improved photodegradation from the 25th percentile to above the 40th while also decreasing log *D*_o/w_ from 4.64 to 2.95. These changes satisfy Briggs Rule of 3. Replacing halogens with amine groups led to further gains in both photodegradation (89th percentile) and log *D*_o/w_ (1.61). While these changes resulted in Δ*E* decrease from 6.06 to 5.46, *E*_LUMO_ increased from the 15th to above the 30th percentile of compounds with minimal hazard to aquatic species.

### Outlook

Understanding factors that contribute to safety-performance matrices in pesticide design is crucial to developing next-generation analogs that meet increasing global demand for agricultural products. Rational design of safer chemicals that do not persist in the environment is the cornerstone of green chemistry that rests our interpretation of the underlying structure-property relationships. Here, we showcased a multifaceted approach for developing new pesticides using computationally derived markers of (eco)toxicity, depletion, and function. Our analysis of 700 pesticides provides a wealth of knowledge to chemists and risk assessors alike on the structure-property relationships that drive each design criterion. In a tiered approach, we linked substructural features of pesticides with electronic properties and process metrics to equip the green chemistry community with a pragmatic means of either screening existing pesticides or designing all-around safer and high-performing new molecules.

Our approach represents a fundamental shift toward system-based computational models and the transformation of purely predictive toxicology methods into design tools. It is critical to recognize here that no tool can be truly all encompassing, and safety can never be guaranteed but only maximized in its probability. In that sense, ours is a design-vectoring approach that moves the dial enough to considerably improve safety and depletion profiles of pesticides. However, to progress further, the present model should be integrated with more refined, target-specific methods, such as those described by Clymer *et al.* (*5*). These approaches can elucidate specificity and targeted selectivity of biologically active molecules, addressing a common shortcoming of crop protection agents. In addition, we envision incorporation of tools that assess green process metrics (how we make pesticides), economic cost of production, and, in related terms, synthetic feasibility of proposed analogs. The value proposition here is that an integrated model development paradigm, which bridges advances in predictive toxicology with green chemistry principles, can be adopted across industry sectors to advance our sustainability goals in chemical design. DuPont’s 1935 slogan, paraphrased as “Better Living Through Chemistry,” still rings true in that we have entangled ourselves in needing chemistry to survive; yet, chemistry can also do demonstrable and irreparable harm to us. Humorously, the way forward appears through amending the motto to “Living Through Better Chemistry,” which can only be accomplished by designing “better” chemicals.

## MATERIALS AND METHODS

### Dataset

The current dataset was developed by mining the CompTox Chemicals Dashboard (*35*) for pesticides containing PPRI-oxidizable cores (phenols, anilines, aryl ethers, sulfides, and thiols), which were identified using SMARTS (SMILES arbitrary target specification) patterns (table S3). After removal of duplicate compounds, our search yielded 700 chemicals that matched either a single or multiple oxidizable cores (Table 2). Photodegradation reactions were analyzed for each pesticide with 23 representative CDOM molecules (*17*), spanning six different functional classes (table S4) and 16,100 unique pairwise interactions.

**Table 2.** Pesticides by chemical class. PPRI-oxidizable compounds from the EPA’s pesticide registry (*35*), partitioned by chemical class.

**Functional class**	**Number of matches**
Phenol	93
Aniline	115
Aryl ether	222
Sulfide	183
Thiol	3
Phenol and aniline	11
Phenol and aryl ether	14
Phenol, aniline, and aryl ether	5
Phenol and sulfide	1
Aniline and aryl ether	31
Aniline and sulfide	4
Aryl ether and sulfide	17
Sulfide and thiol	1

### Photodegradation model

We previously reported a tiered computational approach to probe photodegradation kinetics and thermodynamics of pesticides with ^3^CDOM^*^ (*17*). The tiered approach was developed to both increase the efficiency of computational screening for pesticide depletion as well as to fill experimental data gaps and broaden the model’s training set. In tier 1, our method relied on computed free energies of the pesticide-to-^3^CDOM^*^ electron transfer as the rate-determining step of the degradation process, which were fitted to experimental cell potentials (*E*^0^_cell_) and second-order rate constants (log *k*). Calculations at the SMD-M06-2X/6-31 + G(*d*,*p*) level of theory showed that free energies, Δ*G*_et_^0^’s, and barriers, Δ*G*^⧧^’s (the latter estimated from the Marcus theory), correlated well with experiment across a diverse set of 23 CDOMs and 63 pesticides (*R*^2^ > 0.7) and performed even better in class-specific models (*R*^2^ > 0.8) (*17*).

In tier 2, the method leveraged computationally economical electronic parameters based on FMO theory to predict reaction pathway energetics, thus relating electronic properties of pesticides/^3^CDOM^*^ to observed outcomes. Specifically, the gap between HOMO of the pesticide and SOMO (singly occupied molecular orbital) of ^3^CDOM^*^, computed at the mPW1PW91/MIDIX+ level of theory, was successfully correlated to both Δ*G*_et_^0^ and Δ*G*^⧧^ values in univariate linear and nonlinear models (*R*^2^ ~ 0.9, based on ca. 1500 pairwise interactions between ^3^CDOM^*^ and pesticides) (*17*).

In this study, we built on our previously validated approach and extended the predictions of indirect photodegradation to 700 pesticides on the EPA’s registry (*35*). Tier 2 calculations were used to estimate Δ*G*_et_^0^ and Δ*G*^⧧^ values and, by extension, cell potentials (*E*^0^_cell_) and second-order rate constants (log *k*), respectively, from molecular orbital energies as detailed by Lewer *et al.* (*17*). All electronic-structure calculations were carried out using the Gaussian 16 program (*36*).

### Ecotoxicity and performance assessment

Ecotoxicity assessments were carried out using boundary values of key properties related to bioavailability (octanol-water distribution coefficient, log *D*_o/w_, at physiological pH of 7.4) and reactivity (the HOMO-LUMO gap, Δ*E*) (*19*). We have previously reported (*21*, *22*, *26*) that adverse effects to aquatic species are minimized when chemicals have large bandgaps (Δ*E* > 6 eV at the mPW1PW91/MIDIX+ level of theory) and are nonlipophilic (log *D*_o/w_ < 1.7). In general, compounds with smaller bandgaps are softer, indicating greater covalent reactivity (most of the metabolic processes), while larger bandgaps suggest hard-hard (i.e., ionic) interactions. This approach was found robust across all modes of action (MOAs), supported by significant univariate correlations between Δ*E* and toxicity thresholds (*R*^2^ ca. 0.6 to 0.9, depending on the mechanism). Furthermore, this “rule of 2” has been validated on over 1600 chemicals and against standard test species of fish, crustaceans, and green algae (*21*, *22*, *26*). On the basis of these guidelines and depending on the species, ca. 75 to 92% of chemicals studied that were of no or low concern in acute and chronic aquatic toxicity tests fit into this safer chemical space defined by Δ*E* and log *D*_o/w_. Here, log *D*_o/w_ values were estimated using ChemAxon’s cxcalc plugin (Marvin v.6.0, 2013; ChemAxon), and Δ*E* values were calculated with the mPW1PW91/MIDIX+ method using Gaussian 16 software (*36*).

For pesticide active ingredients, we leveraged MOAs’ underlying molecular mechanisms as drivers of both ecotoxicity and performance. This notion is consistent with previous reports, and was made possible by strong correlations identified between Δ*E*/log *D*_o/w_ and toxicity thresholds across all MOAs relevant to pesticide function (fig. S7) (*21*). To that end, molecular perturbations that keep the structures within the bounds of the relevant functional space, and concurrently shift the structures into or toward the safe space (as defined by Δ*E* and log *D*_o/w_ cutoff values), are likely to yield better analogs. This line of reasoning is further supported by our previous work, which showed compounds with increasing Δ*E*, and decreasing log *D*_o/w_ values correspond to proportionally lower level of concern to aquatic species across the entire chemical space tested (*21*).

### Tier 3: Substructural features and design drivers

To incorporate substructure-based design guidelines, we added a third tier into our computational framework. Specifically, we carried out NBO calculations and HPAs on a representative subset of interactions between 44 pesticides (28 phenols and 16 anilines) and 3-methoxyacetophenone (table S5) (*17*). The NBO analysis was used to probe electronic configurations of oxidized (open-shell) forms of pesticides to derive structure-activity relationships based on second-order perturbation theory. NBOs are localized few-center orbitals that describe the Lewis-like molecular bonding pattern of electron pairs or of individual electrons (in the open-shell case of SOMO). The remaining non–Lewis-type NBOs complete the span of the basis and describe delocalization effects, i.e., departure from a single localized Lewis structure (*37*). These orbitals contribute to resonance stabilization, hydrogen bonding, and other forms of donor-acceptor aggregation (*38*) and are, thus, relevant to describing the degree of electron-hole stabilization that occurs in the oxidized pesticide after reacting with ^3^CDOM^*^. Here, we assessed the electron-withdrawing versus electron-donating nature of ring substituents in phenols and anilines by computing the stabilization energy, *E*(2), in donor-acceptor orbital mixing. Pairwise *E*(2) values for interactions between substituent and ring atoms were summed to obtain the total stabilization energy acting on the electron hole, i.e., *E*_stab_ = ∑_subst_ *E*(2)_subst_.

HPA (*39*) was performed across the same subset of pesticide cores to probe the relationship between electron density in the ring and the ability to stabilize the electron hole of the oxidized pesticide. To that end, HPA was selected over the standard Mulliken and Lӧwdin schemes because it provides a clear partitioning of electron density (viz. the Supplementary Materials) (*40*) and is insensitive to basis set size (*41*). Crucially, HPA produces nonnegative and, thus more physically realistic, condensed Fukui function values. Fukui functions reflect the ability of a molecule (or its part) to accept or donate electron density, which is important in capturing the interactions between substituents and the electron-deficient (oxidized) pesticide core (*42*–*48*).

Combining the two analyses, NBO and HPA, affords greater structural understanding of substituents’ effects on photodegradation rates, thereby providing a trajectory for the design of new analogs based on existing pesticide cores. All NBO and HPA calculations were carried out using the Gaussian 16 program (*36*).

## Supplementary Materials

### This PDF file includes:

Supplementary Text Figs. S1 to S7 Tables S1 to S5 References
