# Supplementary material for: Structure-to-process design framework for developing safer pesticides
Source: Sci Adv. 2022 Mar 30;8(13):eabn2058. doi: 10.1126/sciadv.abn2058 (PMC8967227; doi:10.1126/sciadv.abn2058)
Supplement: Supplementary file 1 — Supplementary Text Figs. S1 to S7 Tables S1 to S5 References [file sciadv.abn2058_sm.pdf]

Science Advances
AAAS

Supplementary Materials for

Structure-to-process design framework for developing safer pesticides

Jessica M. Lewer, Zachary R. Stickelman, Jessica H. Huang, John F. Peloquin, Jakub Kostal*

*Corresponding author. Email: jkostal@gwu.edu

Published 30 March 2022, *Sci. Adv.* **8**, eabn2058 (2022)
DOI: 10.1126/sciadv.abn2058

This PDF file includes:

Supplementary Text
Figs. S1 to S7
Tables S1 to S5
References

Supplementary Text

Additional Methods Description:

In Hirschfeld population analysis (HPA), the partitioned charges are defined relative to the "deformation density", $\rho_d(\bar{r})$, which is the difference between the molecular and the pro-molecular density (49, 50) as expressed by,

$$\rho_d(\bar{r}) = \rho^{mol}(\bar{r}) - \rho^{pro}(\bar{r}) = \rho^{mol}(\bar{r}) - \sum_{\alpha} \rho_\alpha(\bar{r} - \bar{R}_\alpha)$$

where $\rho^{mol}(\bar{r})$ is the molecular charge density at a site $\bar{r}$, $\rho^{pro}(\bar{r})$ is the pro-molecular charge density, and $\rho_\alpha(\bar{r} - \bar{R}_\alpha)$ is the spherically averaged ground state charge density of the free atom, α, positioned at $\bar{R}_\alpha$. From here, the effective atomic charge, q_α, is given by,

$$q_\alpha = - \int \rho_d(\bar{r}) w_\alpha(\bar{r}) d^3\bar{r}$$

where $w_\alpha(\bar{r})$ is the "sharing function" (a measure of the share of the atom in the pro-molecular density at position $\bar{r}$.) This sharing function can be expressed as

$$w_\alpha(\bar{r}) = \rho_\alpha(\bar{r} - \bar{R}_\alpha) \left[\sum_{\beta} \rho_\beta(\bar{r} - \bar{R}_\beta) \right]^{-1}$$

with summing over all atoms, β, of the molecule. In HPA, the charge density at each location is divided among the molecule's atoms in proportion to their respective contributions to the pro-molecular density at that point (50).

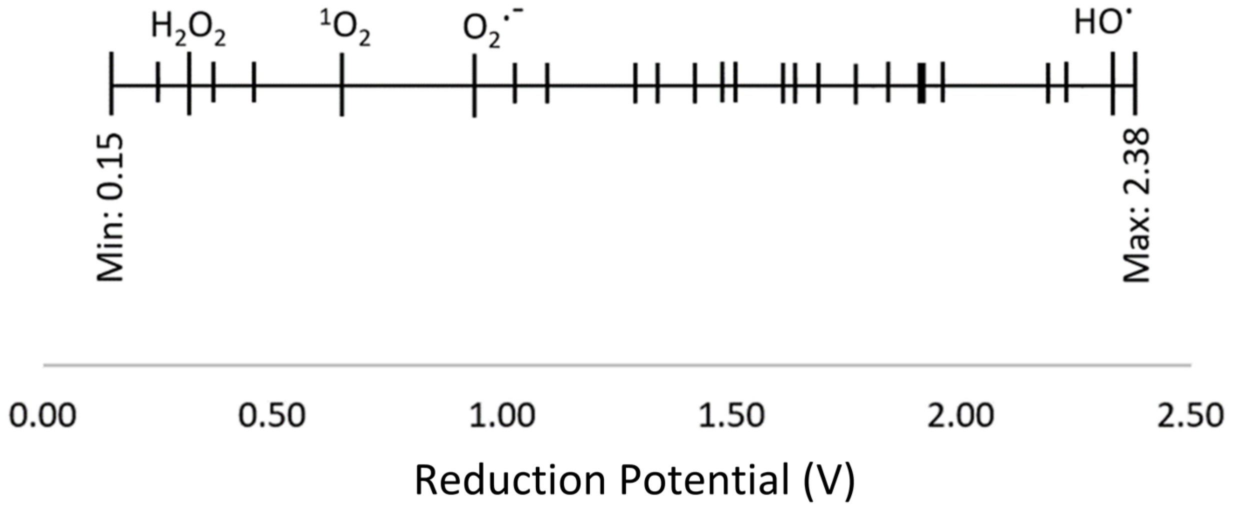

Fig. S1. Reduction potentials of PPRIs. Range of reduction potentials for 23 representative $^3CDOM^*$ molecules used in this study, with reduction potentials of four other prominent PPRIs, hydrogen peroxide (H_2O_2), singlet oxygen ($^1O_2$), superoxide ($O_2\bullet^-$), and hydroxyl radical (HO•), plotted.

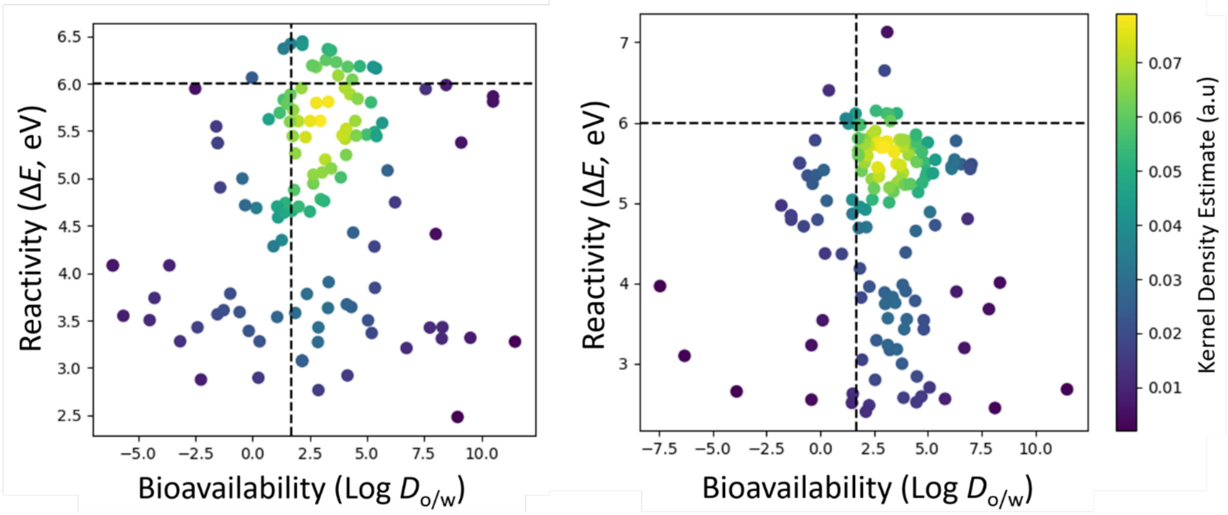

Fig. S2. Density scatter plots of predicted ecotoxicity for phenols and anilines. Octanol-water distribution coefficient (log $D_{o/w}$) vs. energy difference between highest occupied and lowest unoccupied molecular orbitals (ΔE) for all phenol (left) and aniline (right) pesticides. Safer chemical space is defined by the upper left-hand quadrant (log $D_{o/w} < 1.7$, $\Delta E > 6$ eV).

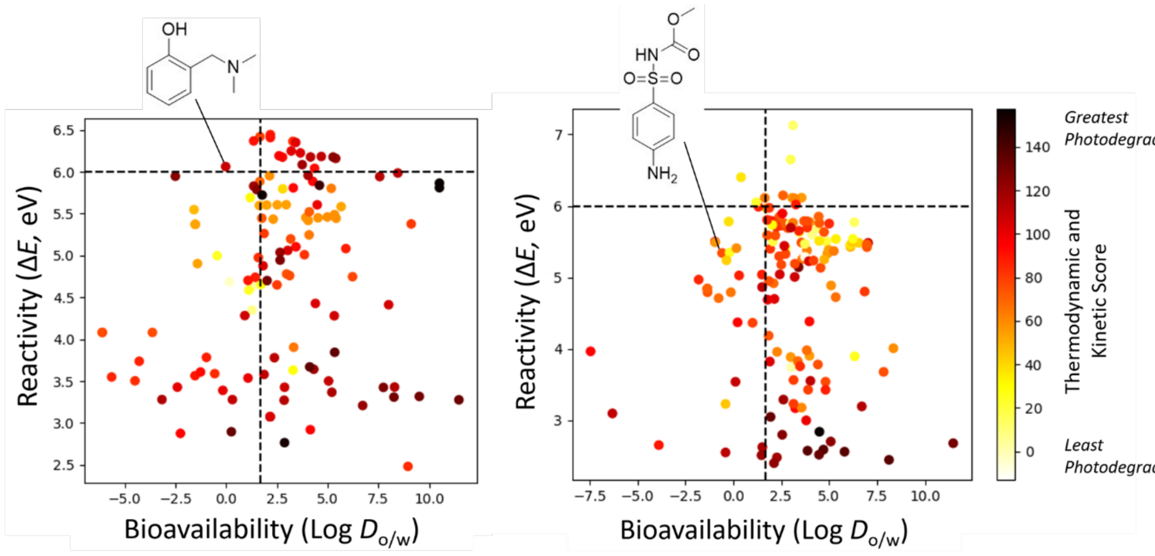

Fig. S3. Scatter plots of predicted ecotoxicity and photodegradation for phenols and anilines. Octanol-water distribution coefficient (log $D_{o/w}$) vs. energy difference between the highest occupied and lowest unoccupied molecular orbitals (ΔE) for phenols (left) and anilines (right). Safer chemical space is defined by the upper left-hand quadrant (log $D_{o/w} < 1.7$, $\Delta E > 6$ eV). Indirect Photodegradation potential is represented by the energy gap between the HOMO of the pesticides and the average SOMO across 23 $^{3}CDOM^{*}$ molecules, where the darker the point, the more likely the molecule is to photodegrade. Structures of two pesticides, 2-dimethylaminomethyl phenol (left) and Asulam (right), which showed the best optimization of degradation and ecotoxicity trade-offs in the entire phenol and aniline datasets, respectively, are displayed.

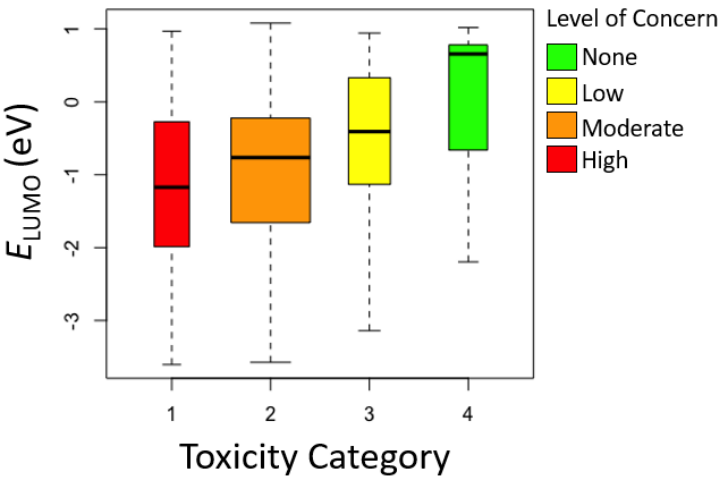

Fig. S4. Ecotoxicity box plot showing distribution of energy of the lowest occupied molecular orbital (E_{LUMO}) by toxicity-concern category. Analysis of 555 compounds studied in the fathead minnow (FHM) acute aquatic toxicity dataset (21).

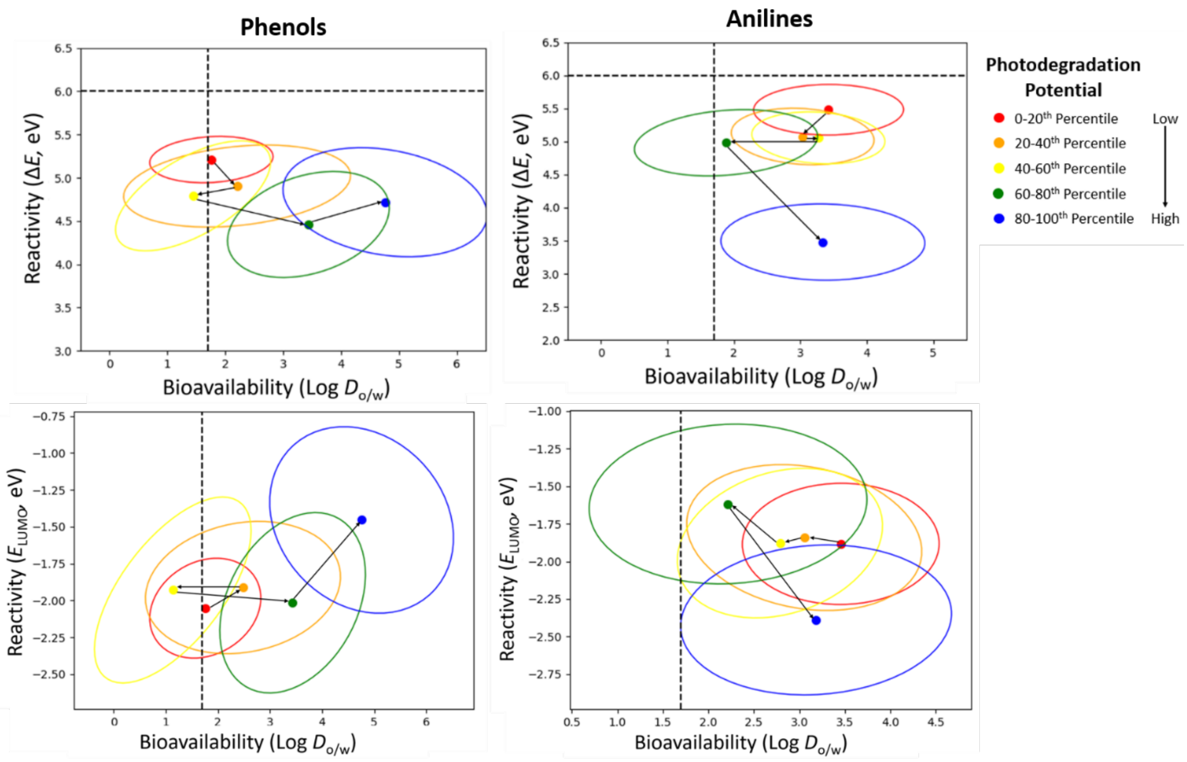

Fig. S5. Coupling of ecotoxicity and photodegradation for phenols and anilines. (A) (top) Scatter plots of ecotoxicity averages (octanol-water distribution coefficient, log $D_{o/w}$ vs. energy difference between the highest occupied and lowest unoccupied molecular orbitals, ΔE) and spread (ellipse radii based on half standard deviation in the x and y direction) for each percentile bracket of photodegradation potential for phenols (right) and anilines (left). Black arrows represent vectors between adjacent percentile averages. **(B) (bottom)** Scatter plots of ecotoxicity averages (octanol-water distribution coefficient, log $D_{o/w}$ vs. energy of the lowest unoccupied molecular orbital, E_{LUMO}).

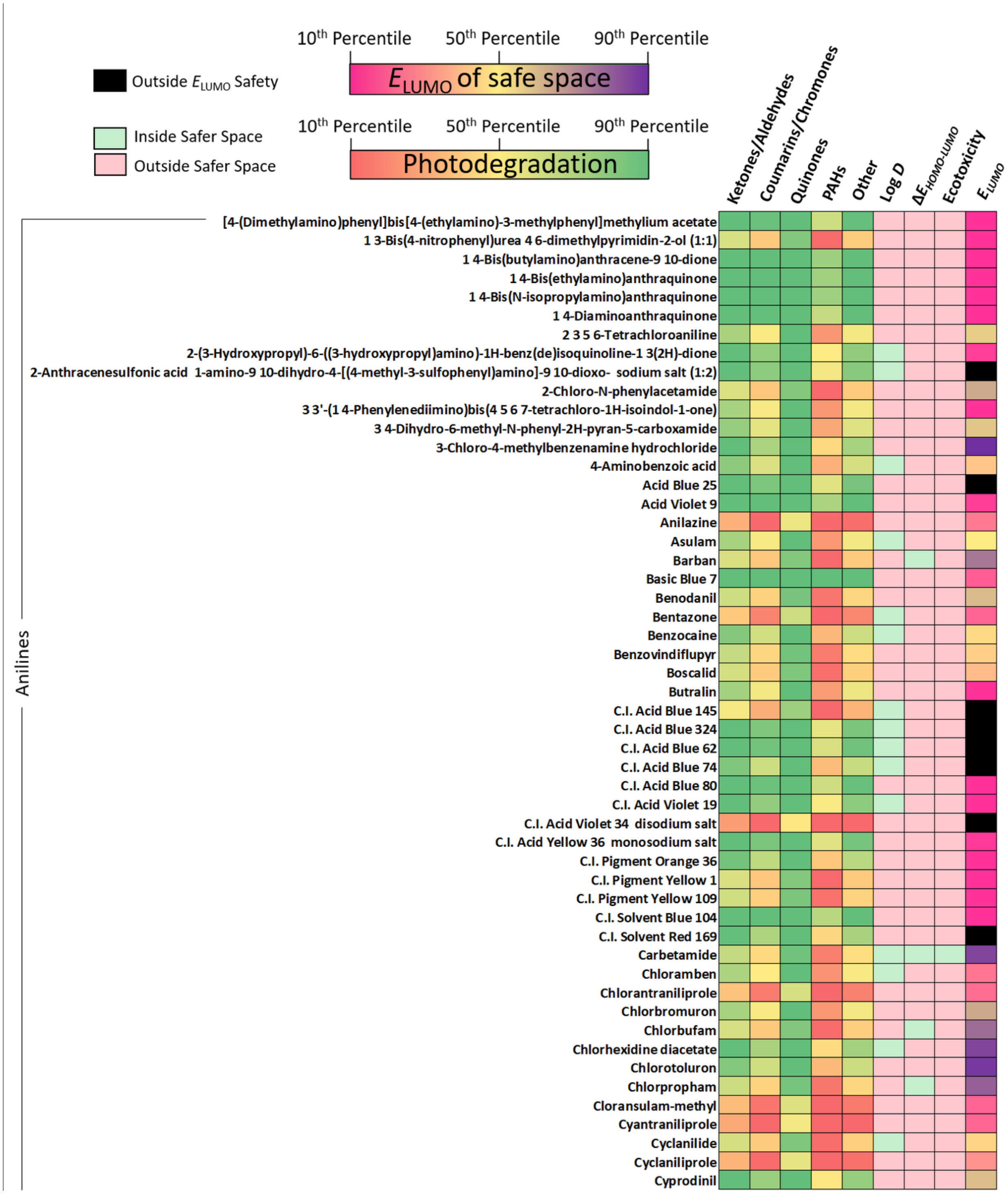

Outside E_{LUMO} Safety
Inside Safer Space
Outside Safer Space
10th Percentile
50th Percentile
90th Percentile
E_{LUMO} of safe space
10th Percentile
50th Percentile
90th Percentile
Photodegradation
Ketones/Aldehydes
Coumarins/Chromones
Quinones
PAHs
Other
Log D
$\Delta E_{HOMO-LUMO}$
Ecotoxicity
E_{LUMO}
Anilines
[4-(Dimethylamino)phenyl]bis[4-(ethylamino)-3-methylphenyl]methylium acetate
1 3-Bis(4-nitrophenyl)urea 4 6-dimethylpyrimidin-2-ol (1:1)
1 4-Bis(butylamino)anthracene-9 10-dione
1 4-Bis(ethylamino)anthraquinone
1 4-Bis(N-isopropylamino)anthraquinone
1 4-Diaminoanthraquinone
2 3 5 6-Tetrachloroaniline
2-(3-Hydroxypropyl)-6-((3-hydroxypropyl)amino)-1H-benz(de)isoquinoline-1 3(2H)-dione
2-Anthracenesulfonic acid 1-amino-9 10-dihydro-4-[(4-methyl-3-sulfophenyl)amino]-9 10-dioxo- sodium salt (1:2)
2-Chloro-N-phenylacetamide
3 3'-(1 4-Phenylenediimino)bis(4 5 6 7-tetrachloro-1H-isoindol-1-one)
3 4-Dihydro-6-methyl-N-phenyl-2H-pyran-5-carboxamide
3-Chloro-4-methylbenzenamine hydrochloride
4-Aminobenzoic acid
Acid Blue 25
Acid Violet 9
Anilazine
Asulam
Barban
Basic Blue 7
Benodanil
Bentazone
Benzocaine
Benzovindiflupyr
Boscalid
Butralin
C.I. Acid Blue 145
C.I. Acid Blue 324
C.I. Acid Blue 62
C.I. Acid Blue 74
C.I. Acid Blue 80
C.I. Acid Violet 19
C.I. Acid Violet 34 disodium salt
C.I. Acid Yellow 36 monosodium salt
C.I. Pigment Orange 36
C.I. Pigment Yellow 1
C.I. Pigment Yellow 109
C.I. Solvent Blue 104
C.I. Solvent Red 169
Carbetamide
Chloramben
Chlorantraniliprole
Chlorbromuron
Chlorbufam
Chlorhexidine diacetate
Chlorotoluron
Chlorpropham
Cloransulam-methyl
Cyantraniliprole
Cyclanilide
Cyclaniliprole
Cyprodinil

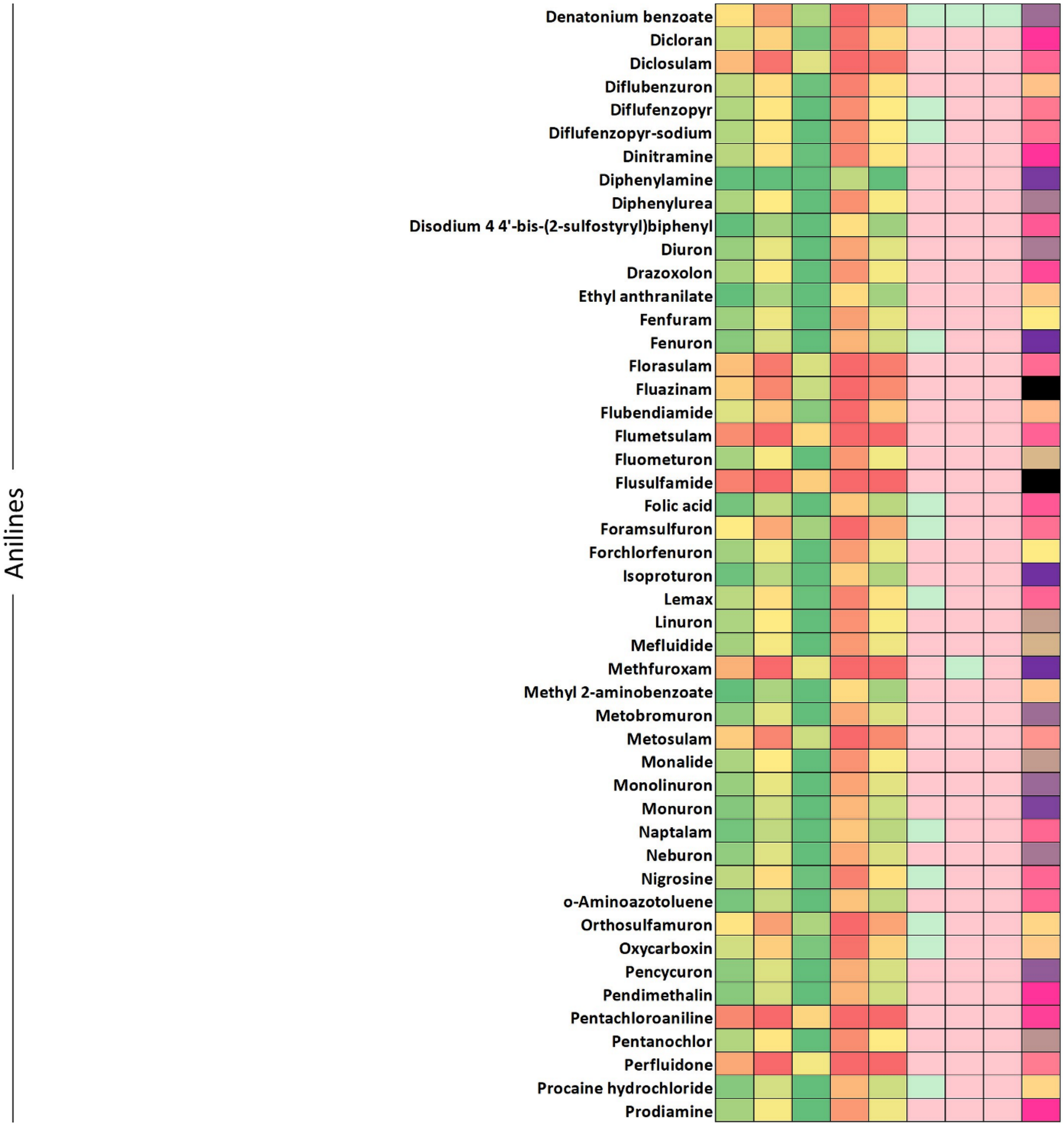
Anilines
Denatonium benzoate
Dicloran
Diclosulam
Diflubenzuron
Diflufenzopyr
Diflufenzopyr-sodium
Dinitramine
Diphenylamine
Diphenylurea
Disodium 4 4'-bis-(2-sulfostyryl)biphenyl
Diuron
Drazoxolon
Ethyl anthranilate
Fenfuram
Fenuron
Florasulam
Fluazinam
Flubendiamide
Flumetsulam
Fluometuron
Flusulfamide
Folic acid
Foramsulfuron
Forchlorfenuron
Isoproturon
Lemax
Linuron
Mefluidide
Methfuroxam
Methyl 2-aminobenzoate
Metobromuron
Metosulam
Monalide
Monolinuron
Monuron
Naptalam
Neburon
Nigrosine
o-Aminoazotoluene
Orthosulfamuron
Oxycarboxin
Pencycuron
Pendimethalin
Pentachloroaniline
Pentanochlor
Perfluidone
Procaine hydrochloride
Prodiamine

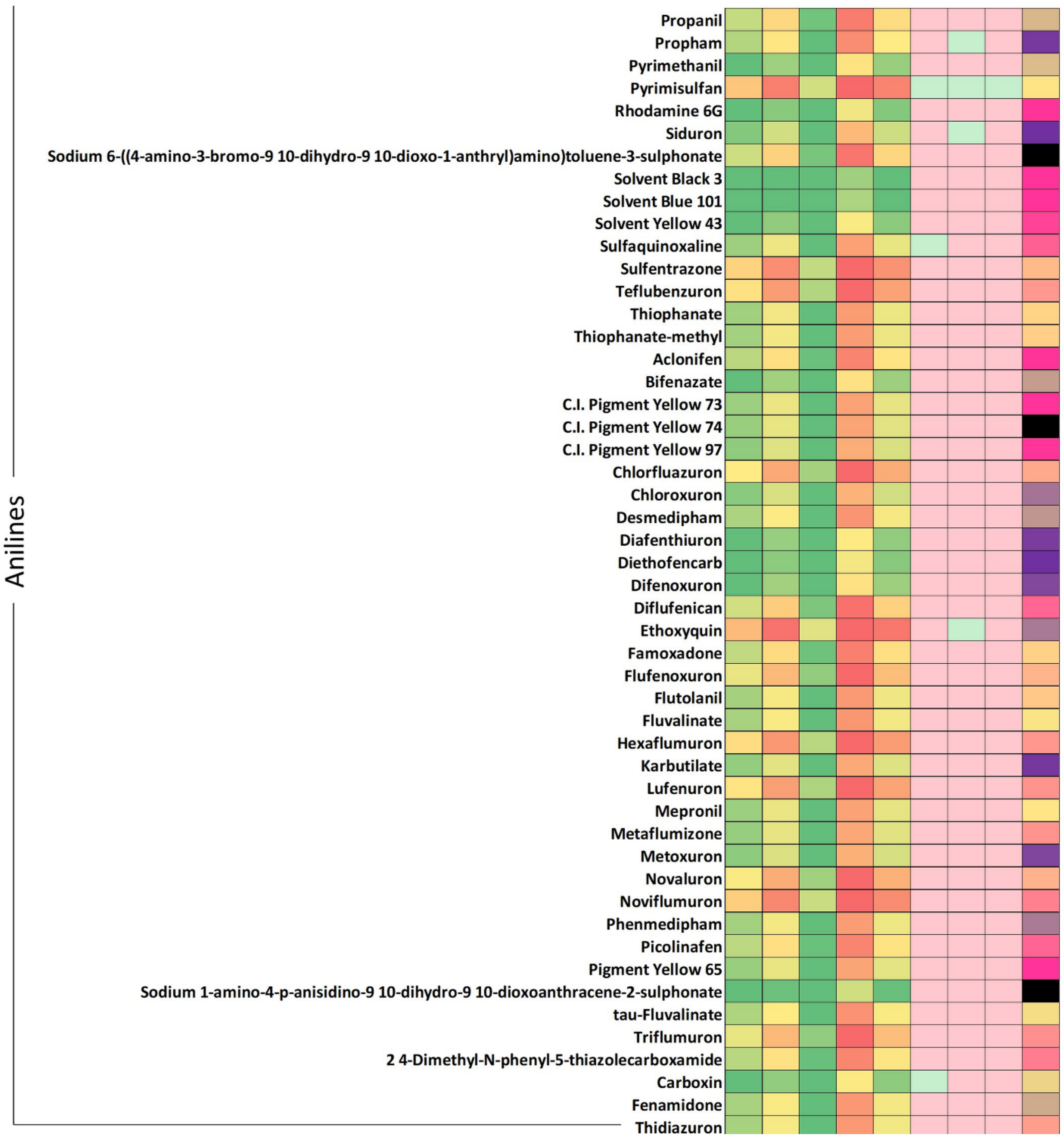
Anilines
Propanil
Propham
Pyrimethanil
Pyrimisulfan
Rhodamine 6G
Siduron
Sodium 6-((4-amino-3-bromo-9 10-dihydro-9 10-dioxo-1-anthryl)amino)toluene-3-sulphonate
Solvent Black 3
Solvent Blue 101
Solvent Yellow 43
Sulfaquinoxaline
Sulfentrazone
Teflubenzuron
Thiophanate
Thiophanate-methyl
Aclonifen
Bifenazate
C.I. Pigment Yellow 73
C.I. Pigment Yellow 74
C.I. Pigment Yellow 97
Chlorfluazuron
Chloroxuron
Desmedipham
Diafenthiuron
Diethofencarb
Difenoxuron
Diflufenican
Ethoxyquin
Famoxadone
Flufenoxuron
Flutolanil
Fluvalinate
Hexaflumuron
Karbutilate
Lufenuron
Mepronil
Metaflumizone
Metoxuron
Novaluron
Noviflumuron
Phenmedipham
Picolinafen
Pigment Yellow 65
Sodium 1-amino-4-p-anisidino-9 10-dihydro-9 10-dioxoanthracene-2-sulphonate
tau-Fluvalinate
Triflumuron
2 4-Dimethyl-N-phenyl-5-thiazolecarboxamide
Carboxin
Fenamidone
Thidiazuron

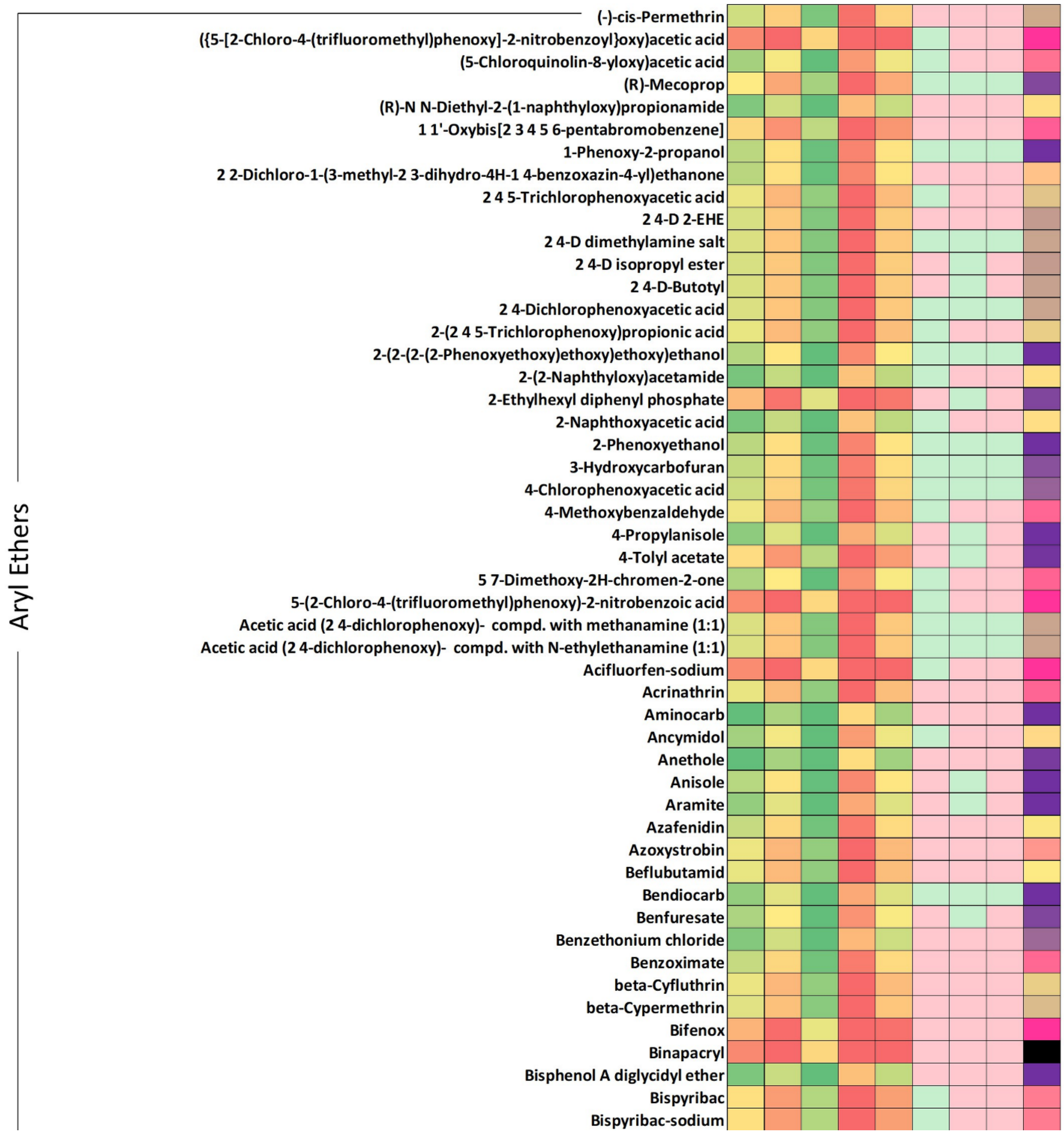
Aryl Ethers
(-)-cis-Permethrin
({5-[2-Chloro-4-(trifluoromethyl)phenoxy]-2-nitrobenzoyl}oxy)acetic acid
(5-Chloroquinolin-8-yloxy)acetic acid
(R)-Mecoprop
(R)-N N-Diethyl-2-(1-naphthyloxy)propionamide
1 1'-Oxybis[2 3 4 5 6-pentabromobenzene]
1-Phenoxy-2-propanol
2 2-Dichloro-1-(3-methyl-2 3-dihydro-4H-1 4-benzoxazin-4-yl)ethanone
2 4 5-Trichlorophenoxyacetic acid
2 4-D 2-EHE
2 4-D dimethylamine salt
2 4-D isopropyl ester
2 4-D-Butotyl
2 4-Dichlorophenoxyacetic acid
2-(2 4 5-Trichlorophenoxy)propionic acid
2-(2-(2-(2-Phenoxyethoxy)ethoxy)ethoxy)ethanol
2-(2-Naphthyloxy)acetamide
2-Ethylhexyl diphenyl phosphate
2-Naphthoxyacetic acid
2-Phenoxyethanol
3-Hydroxycarbofuran
4-Chlorophenoxyacetic acid
4-Methoxybenzaldehyde
4-Propylanisole
4-Tolyl acetate
5 7-Dimethoxy-2H-chromen-2-one
5-(2-Chloro-4-(trifluoromethyl)phenoxy)-2-nitrobenzoic acid
Acetic acid (2 4-dichlorophenoxy)- compd. with methanamine (1:1)
Acetic acid (2 4-dichlorophenoxy)- compd. with N-ethylethanamine (1:1)
Acifluorfen-sodium
Acrinathrin
Aminocarb
Ancymidol
Anethole
Anisole
Aramite
Azafenidin
Azoxystrobin
Beflubutamid
Bendiocarb
Benfuresate
Benzethonium chloride
Benzoximate
beta-Cyfluthrin
beta-Cypermethrin
Bifenox
Binapacryl
Bisphenol A diglycidyl ether
Bispyribac
Bispyribac-sodium

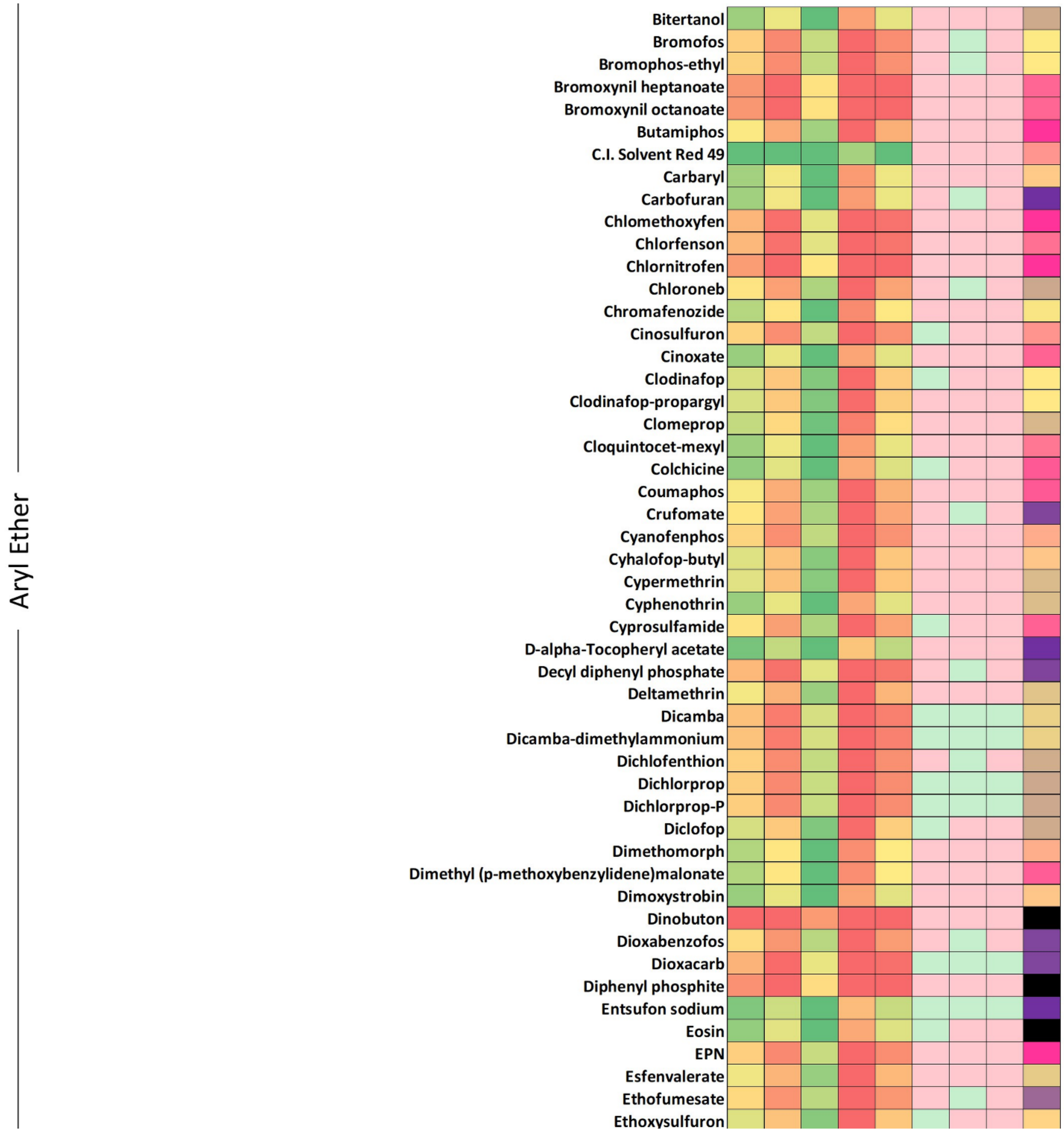
Aryl Ether
Bitertanol
Bromofos
Bromophos-ethyl
Bromoxynil heptanoate
Bromoxynil octanoate
Butamiphos
C.I. Solvent Red 49
Carbaryl
Carbofuran
Chlomethoxyfen
Chlorfenson
Chlornitrofen
Chloroneb
Chromafenozide
Cinosulfuron
Cinoxate
Clodinafop
Clodinafop-propargyl
Clomeprop
Cloquintocet-mexyl
Colchicine
Coumaphos
Crufomate
Cyanofenphos
Cyhalofop-butyl
Cypermethrin
Cyphenothrin
Cyprosulfamide
D-alpha-Tocopheryl acetate
Decyl diphenyl phosphate
Deltamethrin
Dicamba
Dicamba-dimethylammonium
Dichlofenthion
Dichlorprop
Dichlorprop-P
Diclofop
Dimethomorph
Dimethyl (p-methoxybenzylidene)malonate
Dimoxystrobin
Dinobuton
Dioxabenzofos
Dioxacarb
Diphenyl phosphite
Entsufon sodium
Eosin
EPN
Esfenvalerate
Ethofumesate
Ethoxysulfuron

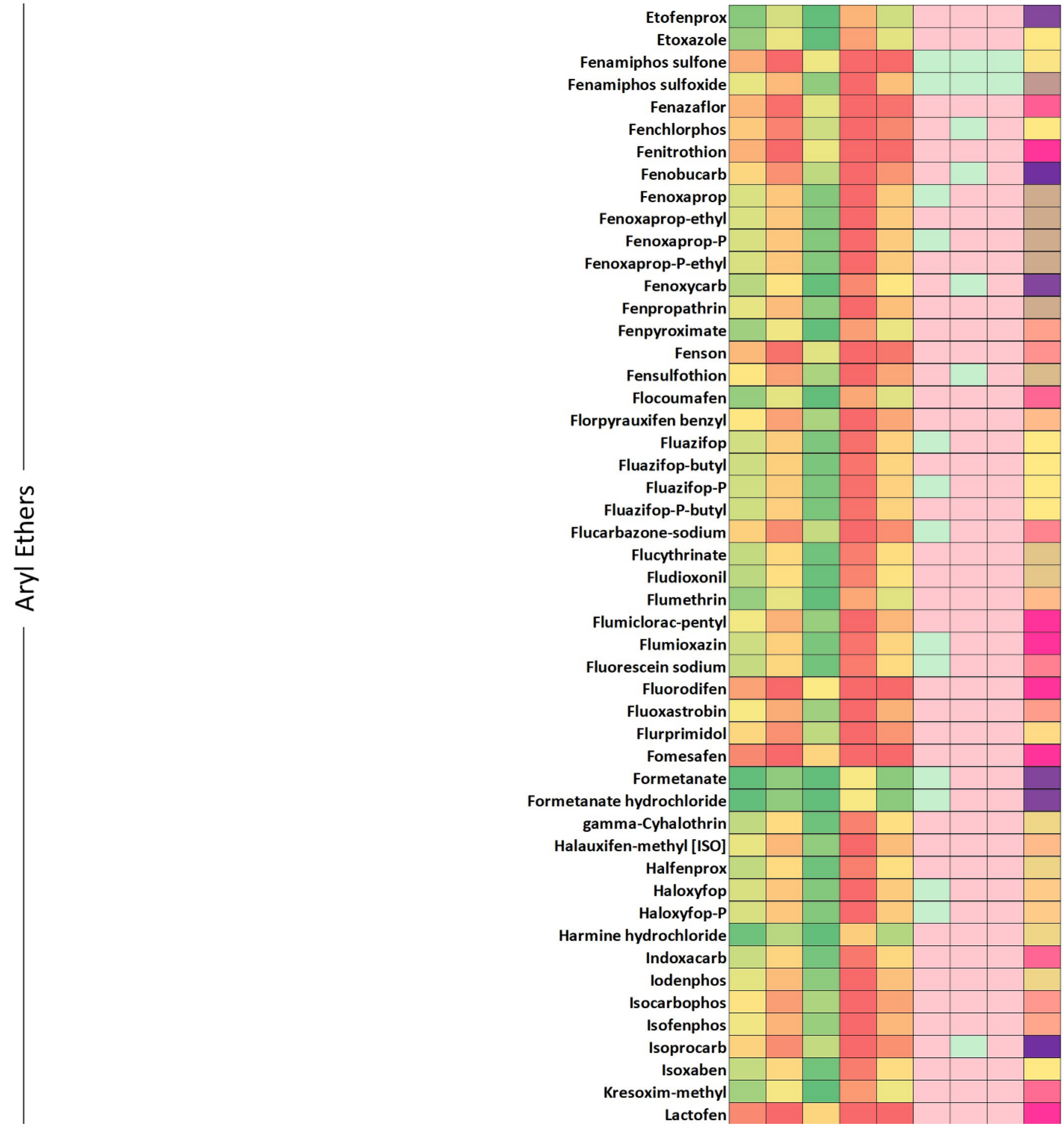
Aryl Ethers
Etofenprox
Etoxazole
Fenamiphos sulfone
Fenamiphos sulfoxide
Fenazaflor
Fenchlorphos
Fenitrothion
Fenobucarb
Fenoxaprop
Fenoxaprop-ethyl
Fenoxaprop-P
Fenoxaprop-P-ethyl
Fenoxycarb
Fenpropathrin
Fenpyroximate
Fenson
Fensulfothion
Flocoumafen
Florpyrauxifen benzyl
Fluazifop
Fluazifop-butyl
Fluazifop-P
Fluazifop-P-butyl
Flucarbazone-sodium
Flucythrinate
Fludioxonil
Flumethrin
Flumiclorac-pentyl
Flumioxazin
Fluorescein sodium
Fluorodifen
Fluoxastrobin
Flurprimidol
Fomesafen
Formetanate
Formetanate hydrochloride
gamma-Cyhalothrin
Halauxifen-methyl [ISO]
Halfenprox
Haloxyfop
Haloxyfop-P
Harmine hydrochloride
Indoxacarb
Iodenphos
Isocarbophos
Isofenphos
Isoprocarb
Isoxaben
Kresoxim-methyl
Lactofen

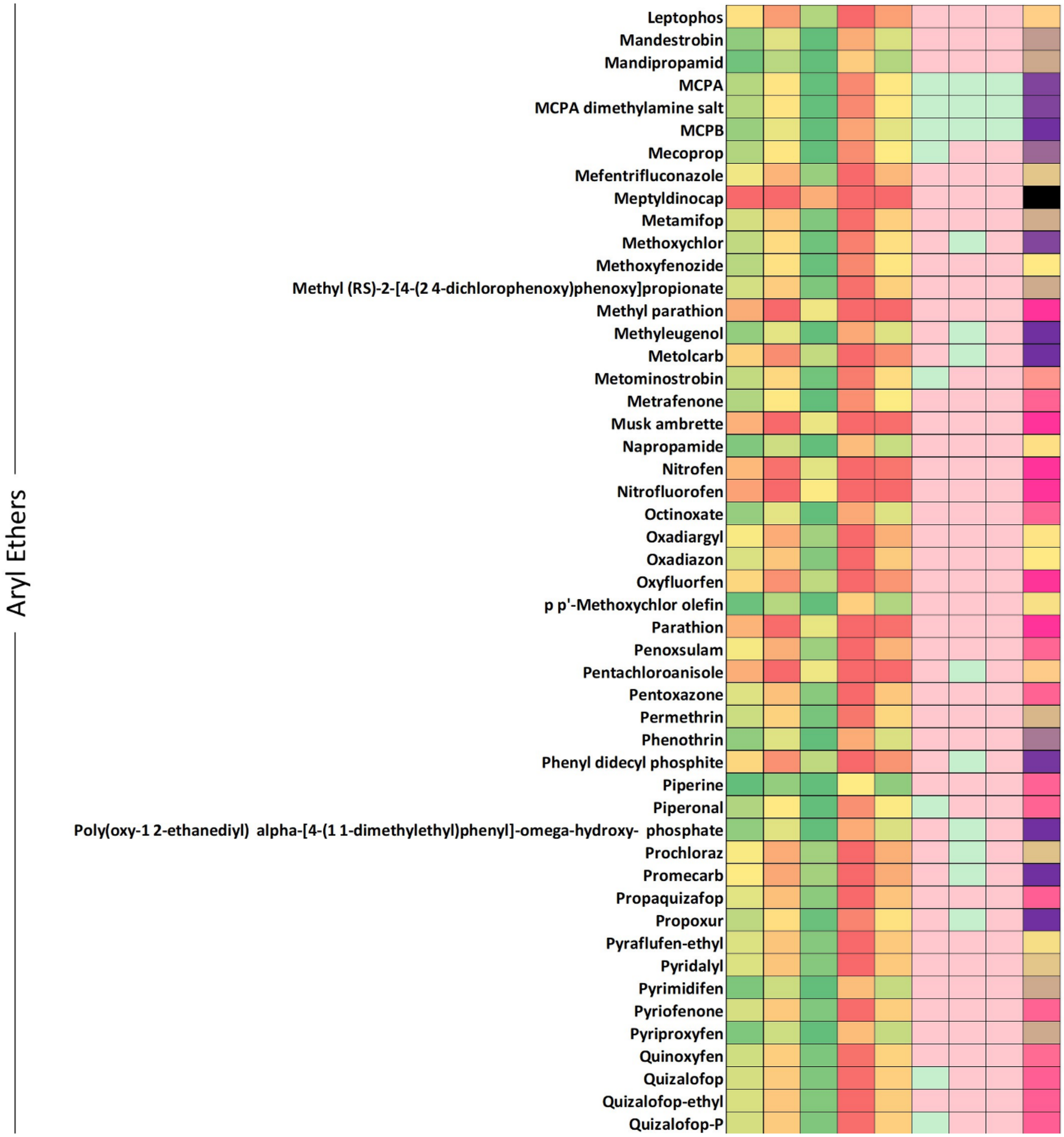
Aryl Ethers
Leptophos
Mandestrobin
Mandipropamid
MCPA
MCPA dimethylamine salt
MCPB
Mecoprop
Mefentrifluconazole
Meptyldinocap
Metamifop
Methoxychlor
Methoxyfenozide
Methyl (RS)-2-[4-(2 4-dichlorophenoxy)phenoxy]propionate
Methyl parathion
Methyleugenol
Metolcarb
Metominostrobin
Metrafenone
Musk ambrette
Napropamide
Nitrofen
Nitrofluorofen
Octinoxate
Oxadiargyl
Oxadiazon
Oxyfluorfen
p p'-Methoxychlor olefin
Parathion
Penoxsulam
Pentachloroanisole
Pentoxazone
Permethrin
Phenothrin
Phenyl didecyl phosphite
Piperine
Piperonal
Poly(oxy-1 2-ethanediyl) alpha-[4-(1 1-dimethylethyl)phenyl]-omega-hydroxy- phosphate
Prochloraz
Promecarb
Propaquizafop
Propoxur
Pyraflufen-ethyl
Pyridalyl
Pyrimidifen
Pyriofenone
Pyriproxyfen
Quinoxyfen
Quizalofop
Quizalofop-ethyl
Quizalofop-P

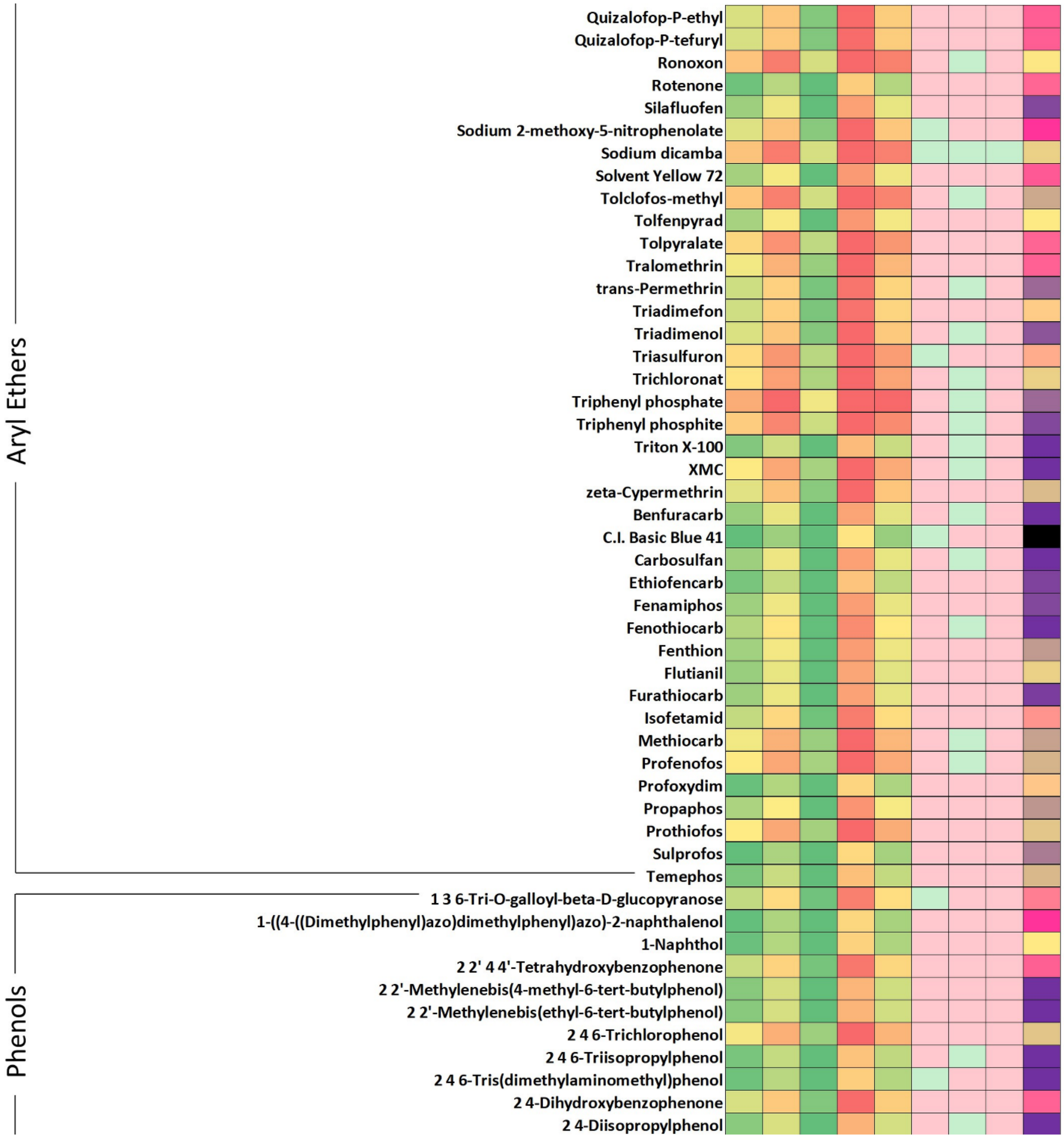
Aryl Ethers
Quizalofop-P-ethyl
Quizalofop-P-tefuryl
Ronoxon
Rotenone
Silafluofen
Sodium 2-methoxy-5-nitrophenolate
Sodium dicamba
Solvent Yellow 72
Tolclofos-methyl
Tolfenpyrad
Tolpyralate
Tralomethrin
trans-Permethrin
Triadimefon
Triadimenol
Triasulfuron
Trichloronat
Triphenyl phosphate
Triphenyl phosphite
Triton X-100
XMC
zeta-Cypermethrin
Benfuracarb
C.I. Basic Blue 41
Carbosulfan
Ethiofencarb
Fenamiphos
Fenothiocarb
Fenthion
Flutianil
Furathiocarb
Isofetamid
Methiocarb
Profenofos
Profoxydim
Propaphos
Prothiofos
Sulprofos
Temephos
Phenols
1 3 6-Tri-O-galloyl-beta-D-glucopyranose
1-((4-((Dimethylphenyl)azo)dimethylphenyl)azo)-2-naphthalenol
1-Naphthol
2 2' 4 4'-Tetrahydroxybenzophenone
2 2'-Methylenebis(4-methyl-6-tert-butylphenol)
2 2'-Methylenebis(ethyl-6-tert-butylphenol)
2 4 6-Trichlorophenol
2 4 6-Triisopropylphenol
2 4 6-Tris(dimethylaminomethyl)phenol
2 4-Dihydroxybenzophenone
2 4-Diisopropylphenol

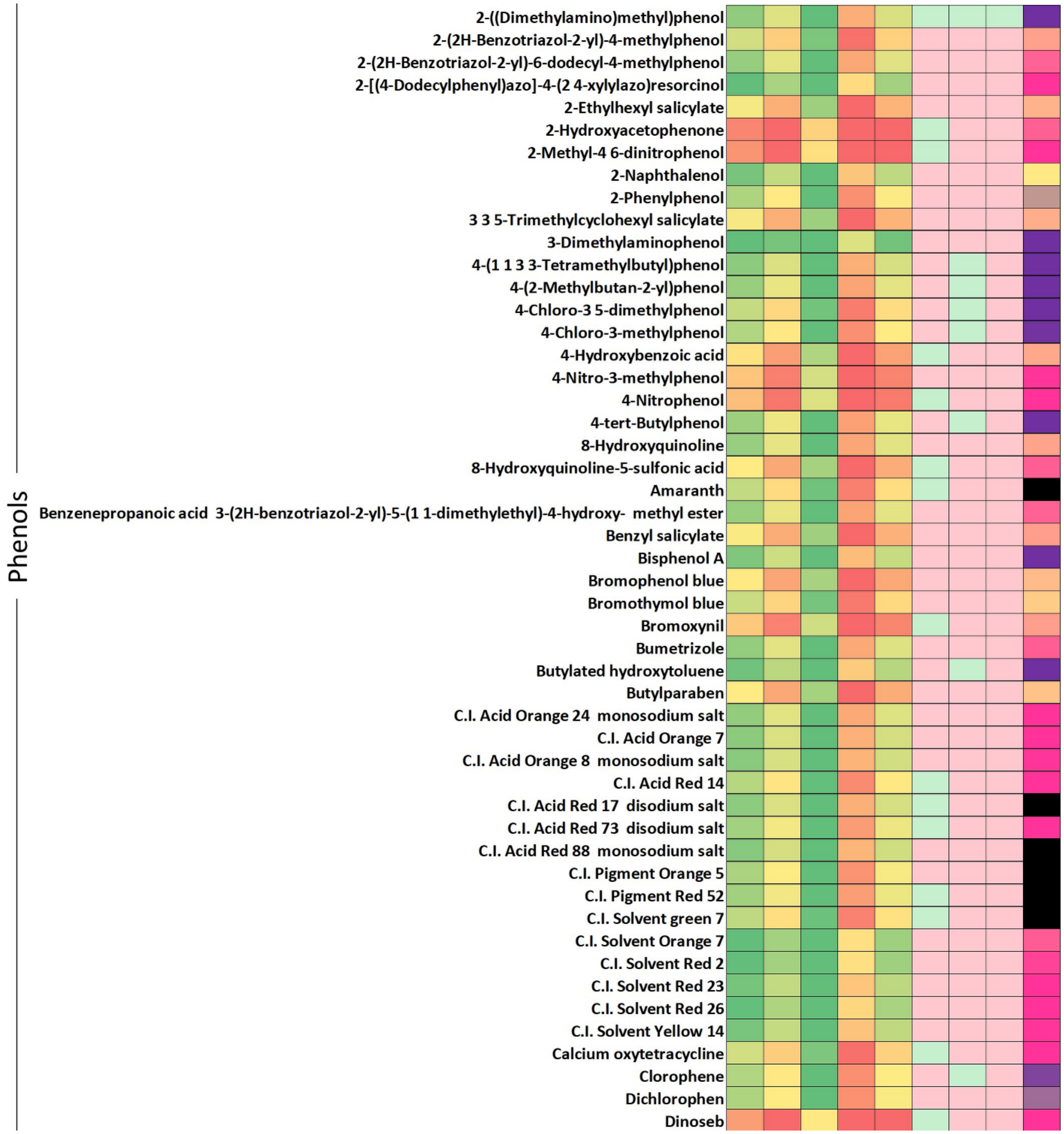
Phenols
2-((Dimethylamino)methyl)phenol
2-(2H-Benzotriazol-2-yl)-4-methylphenol
2-(2H-Benzotriazol-2-yl)-6-dodecyl-4-methylphenol
2-[(4-Dodecylphenyl)azo]-4-(2 4-xylylazo)resorcinol
2-Ethylhexyl salicylate
2-Hydroxyacetophenone
2-Methyl-4 6-dinitrophenol
2-Naphthalenol
2-Phenylphenol
3 3 5-Trimethylcyclohexyl salicylate
3-Dimethylaminophenol
4-(1 1 3 3-Tetramethylbutyl)phenol
4-(2-Methylbutan-2-yl)phenol
4-Chloro-3 5-dimethylphenol
4-Chloro-3-methylphenol
4-Hydroxybenzoic acid
4-Nitro-3-methylphenol
4-Nitrophenol
4-tert-Butylphenol
8-Hydroxyquinoline
8-Hydroxyquinoline-5-sulfonic acid
Amaranth
Benzenepropanoic acid 3-(2H-benzotriazol-2-yl)-5-(1 1-dimethylethyl)-4-hydroxy- methyl ester
Benzyl salicylate
Bisphenol A
Bromophenol blue
Bromothymol blue
Bromoxynil
Bumetrizole
Butylated hydroxytoluene
Butylparaben
C.I. Acid Orange 24 monosodium salt
C.I. Acid Orange 7
C.I. Acid Orange 8 monosodium salt
C.I. Acid Red 14
C.I. Acid Red 17 disodium salt
C.I. Acid Red 73 disodium salt
C.I. Acid Red 88 monosodium salt
C.I. Pigment Orange 5
C.I. Pigment Red 52
C.I. Solvent green 7
C.I. Solvent Orange 7
C.I. Solvent Red 2
C.I. Solvent Red 23
C.I. Solvent Red 26
C.I. Solvent Yellow 14
Calcium oxytetracycline
Clorophene
Dichlorophen
Dinoseb

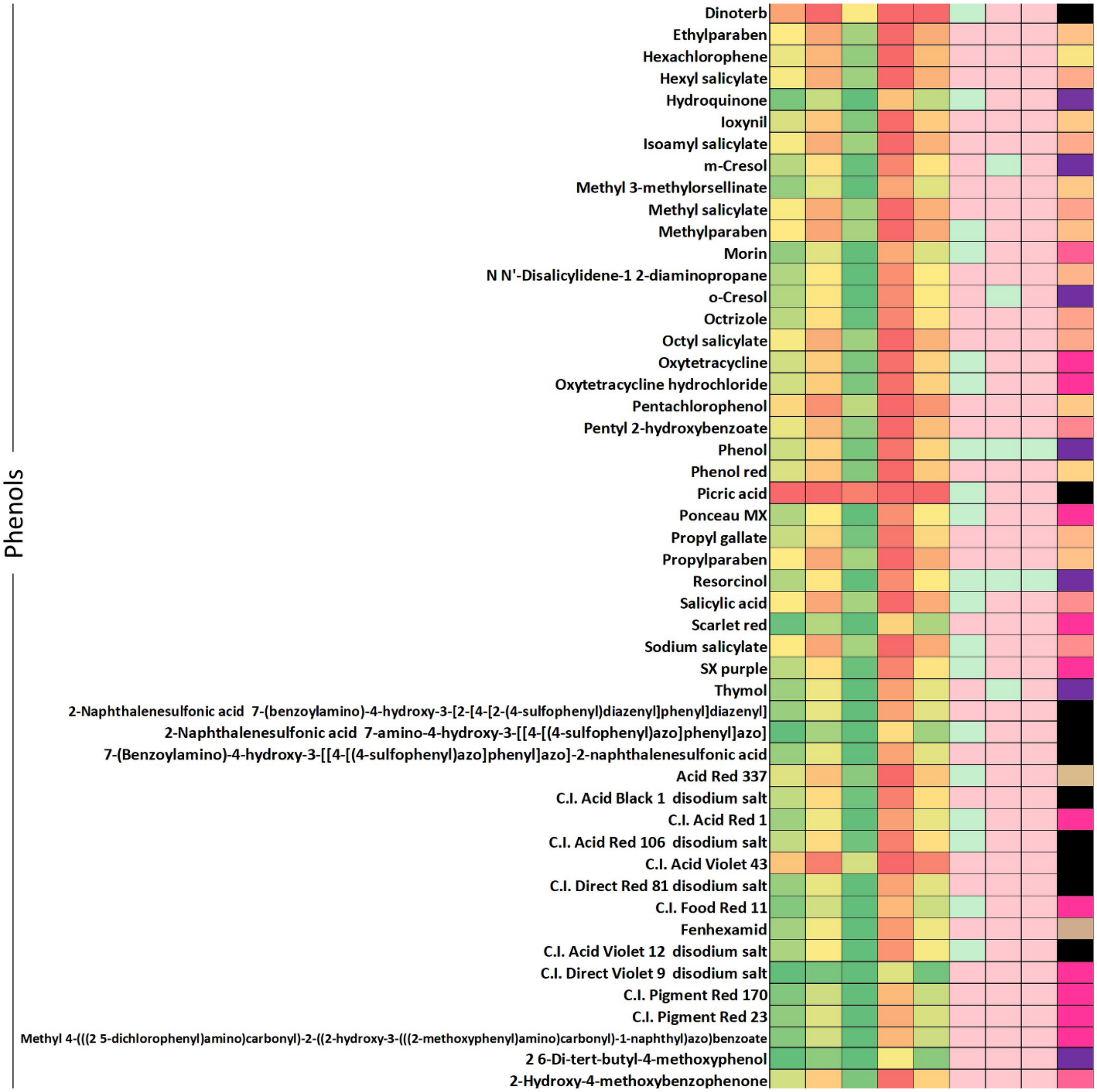
Phenols
Dinoterb
Ethylparaben
Hexachlorophene
Hexyl salicylate
Hydroquinone
Ioxynil
Isoamyl salicylate
m-Cresol
Methyl 3-methylorsellinate
Methyl salicylate
Methylparaben
Morin
N N'-Disalicylidene-1 2-diaminopropane
o-Cresol
Octrizole
Octyl salicylate
Oxytetracycline
Oxytetracycline hydrochloride
Pentachlorophenol
Pentyl 2-hydroxybenzoate
Phenol
Phenol red
Picric acid
Ponceau MX
Propyl gallate
Propylparaben
Resorcinol
Salicylic acid
Scarlet red
Sodium salicylate
SX purple
Thymol
2-Naphthalenesulfonic acid 7-(benzoylamino)-4-hydroxy-3-[2-[4-[2-(4-sulfophenyl)diazenyl]phenyl]diazenyl]
2-Naphthalenesulfonic acid 7-amino-4-hydroxy-3-[[4-[(4-sulfophenyl)azo]phenyl]azo]
7-(Benzoylamino)-4-hydroxy-3-[[4-[(4-sulfophenyl)azo]phenyl]azo]-2-naphthalenesulfonic acid
Acid Red 337
C.I. Acid Black 1 disodium salt
C.I. Acid Red 1
C.I. Acid Red 106 disodium salt
C.I. Acid Violet 43
C.I. Direct Red 81 disodium salt
C.I. Food Red 11
Fenhexamid
C.I. Acid Violet 12 disodium salt
C.I. Direct Violet 9 disodium salt
C.I. Pigment Red 170
C.I. Pigment Red 23
Methyl 4-(((2 5-dichlorophenyl)amino)carbonyl)-2-((2-hydroxy-3-(((2-methoxyphenyl)amino)carbonyl)-1-naphthyl)azo)benzoate
2 6-Di-tert-butyl-4-methoxyphenol
2-Hydroxy-4-methoxybenzophenone

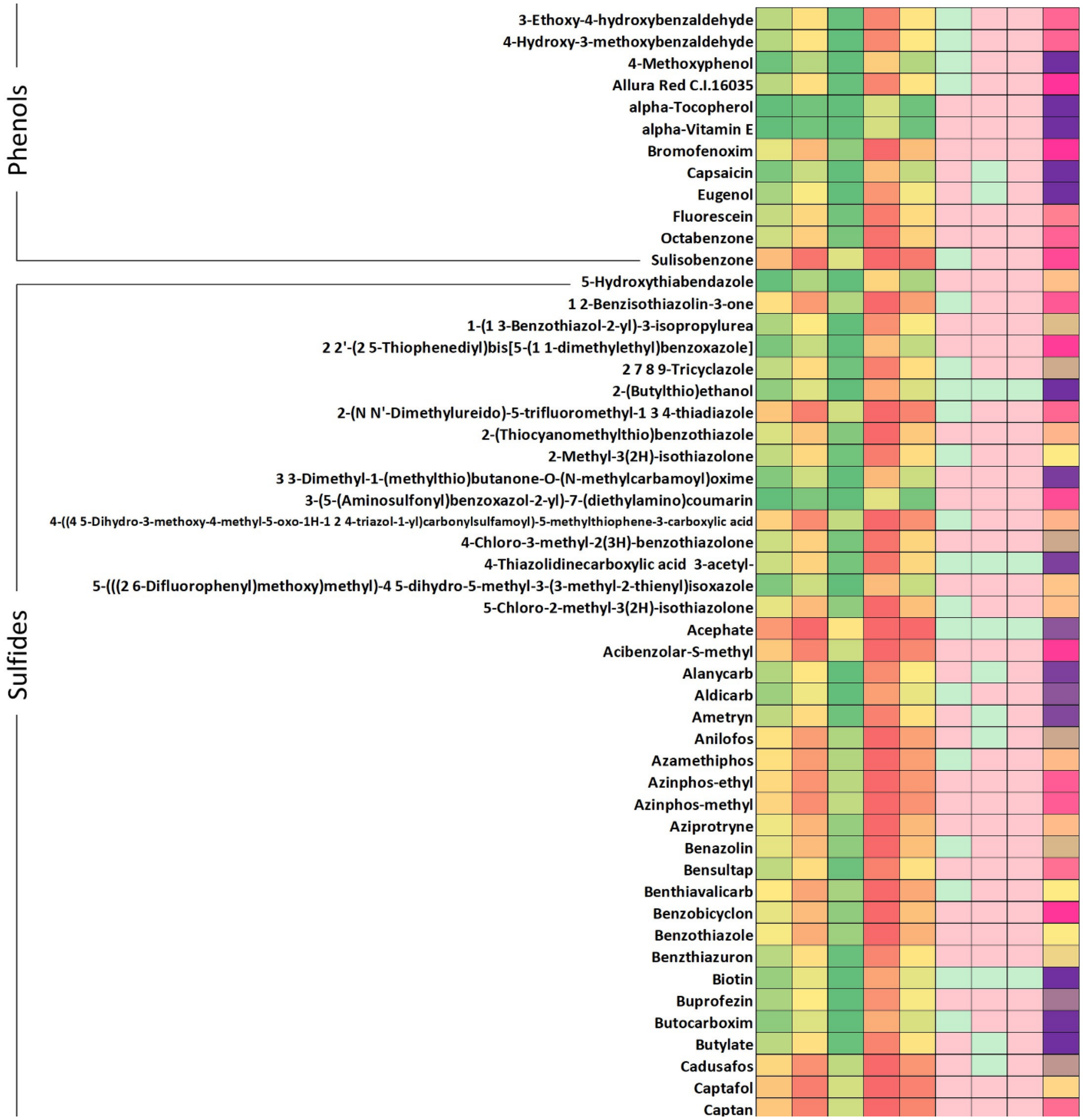
Phenols
3-Ethoxy-4-hydroxybenzaldehyde
4-Hydroxy-3-methoxybenzaldehyde
4-Methoxyphenol
Allura Red C.I.16035
alpha-Tocopherol
alpha-Vitamin E
Bromofenoxim
Capsaicin
Eugenol
Fluorescein
Octabenzone
Sulisobenzone
Sulfides
5-Hydroxythiabendazole
1 2-Benzisothiazolin-3-one
1-(1 3-Benzothiazol-2-yl)-3-isopropylurea
2 2'-(2 5-Thiophenediyl)bis[5-(1 1-dimethylethyl)benzoxazole]
2 7 8 9-Tricyclazole
2-(Butylthio)ethanol
2-(N N'-Dimethylureido)-5-trifluoromethyl-1 3 4-thiadiazole
2-(Thiocyanomethylthio)benzothiazole
2-Methyl-3(2H)-isothiazolone
3 3-Dimethyl-1-(methylthio)butanone-O-(N-methylcarbamoyl)oxime
3-(5-(Aminosulfonyl)benzoxazol-2-yl)-7-(diethylamino)coumarin
4-((4 5-Dihydro-3-methoxy-4-methyl-5-oxo-1H-1 2 4-triazol-1-yl)carbonylsulfamoyl)-5-methylthiophene-3-carboxylic acid
4-Chloro-3-methyl-2(3H)-benzothiazolone
4-Thiazolidinecarboxylic acid 3-acetyl-
5-(((2 6-Difluorophenyl)methoxy)methyl)-4 5-dihydro-5-methyl-3-(3-methyl-2-thienyl)isoxazole
5-Chloro-2-methyl-3(2H)-isothiazolone
Acephate
Acibenzolar-S-methyl
Alanycarb
Aldicarb
Ametryn
Anilofos
Azamethiphos
Azinphos-ethyl
Azinphos-methyl
Aziprotryne
Benazolin
Bensultap
Benthiavalicarb
Benzobicyclon
Benzothiazole
Benzthiazuron
Biotin
Buprofezin
Butocarboxim
Butylate
Cadusafos
Captafol
Captan

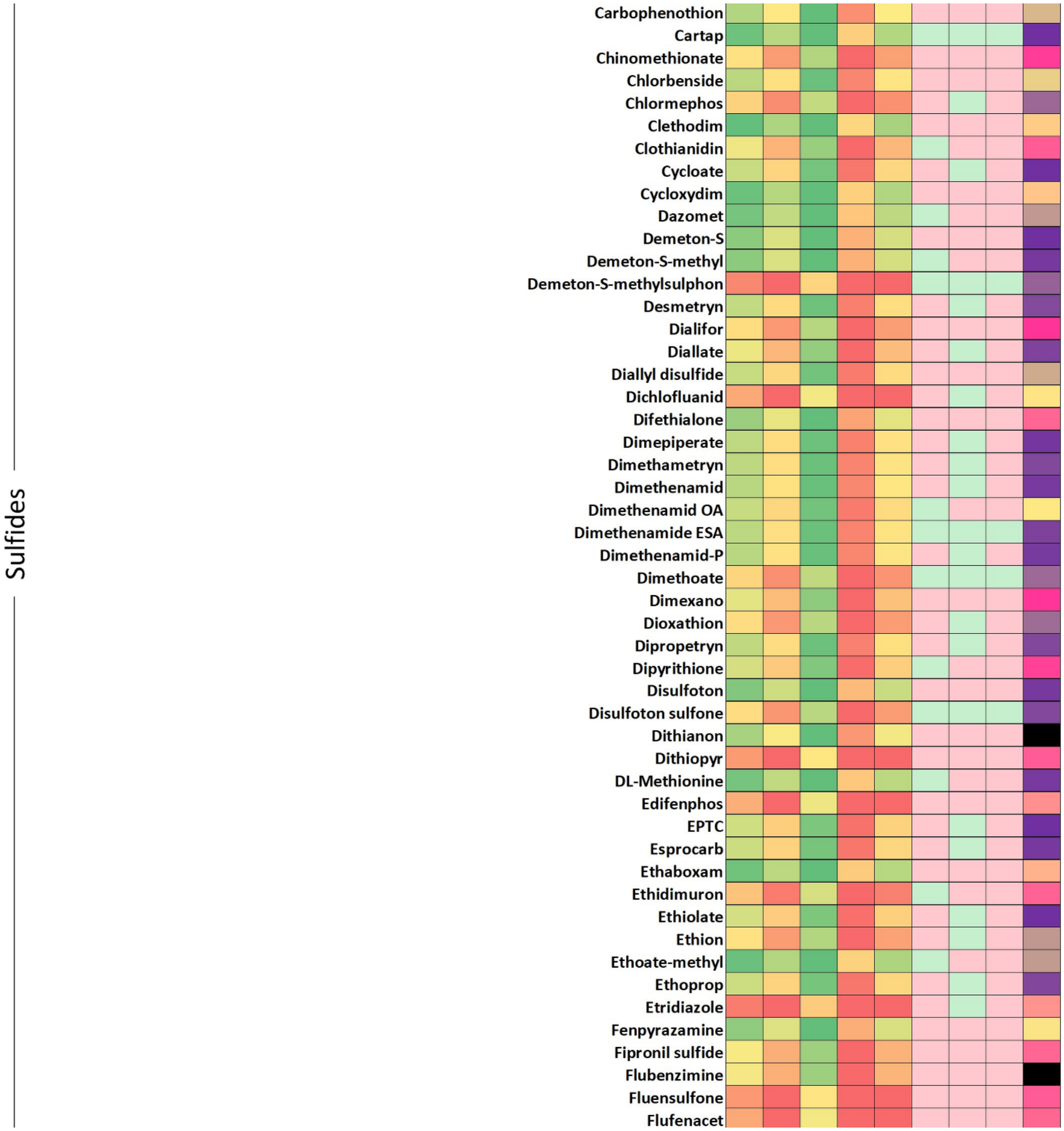
Sulfides
Carbophenothion
Cartap
Chinomethionate
Chlorbenside
Chlormephos
Clethodim
Clothianidin
Cycloate
Cycloxydim
Dazomet
Demeton-S
Demeton-S-methyl
Demeton-S-methylsulphon
Desmetryn
Dialifor
Diallate
Diallyl disulfide
Dichlofluanid
Difethialone
Dimepiperate
Dimethametryn
Dimethenamid
Dimethenamid OA
Dimethenamide ESA
Dimethenamid-P
Dimethoate
Dimexano
Dioxathion
Dipropetryn
Dipyrithione
Disulfoton
Disulfoton sulfone
Dithianon
Dithiopyr
DL-Methionine
Edifenphos
EPTC
Esprocarb
Ethaboxam
Ethidimuron
Ethiolate
Ethion
Ethoate-methyl
Ethoprop
Etridiazole
Fenpyrazamine
Fipronil sulfide
Flubenzimine
Fluensulfone
Flufenacet

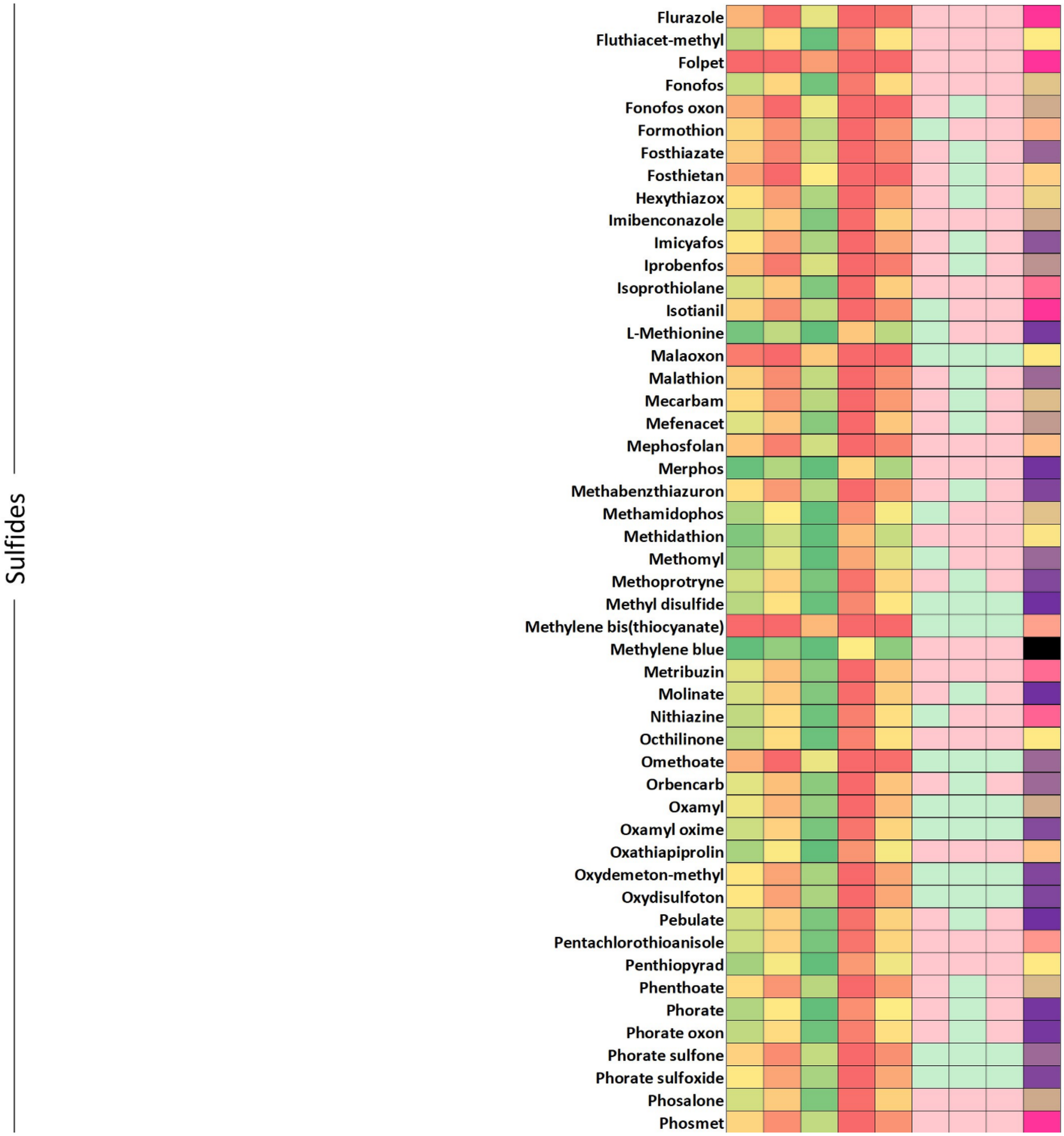
Sulfides
Flurazole
Fluthiacet-methyl
Folpet
Fonofos
Fonofos oxon
Formothion
Fosthiazate
Fosthietan
Hexythiazox
Imibenconazole
Imicyafos
Iprobenfos
Isoprothiolane
Isotianil
L-Methionine
Malaoxon
Malathion
Mecarbam
Mefenacet
Mephosfolan
Merphos
Methabenzthiazuron
Methamidophos
Methidathion
Methomyl
Methoprotryne
Methyl disulfide
Methylene bis(thiocyanate)
Methylene blue
Metribuzin
Molinate
Nithiazine
Octhilinone
Omethoate
Orbencarb
Oxamyl
Oxamyl oxime
Oxathiapiprolin
Oxydemeton-methyl
Oxydisulfoton
Pebulate
Pentachlorothioanisole
Penthiopyrad
Phenthoate
Phorate
Phorate oxon
Phorate sulfone
Phorate sulfoxide
Phosalone
Phosmet

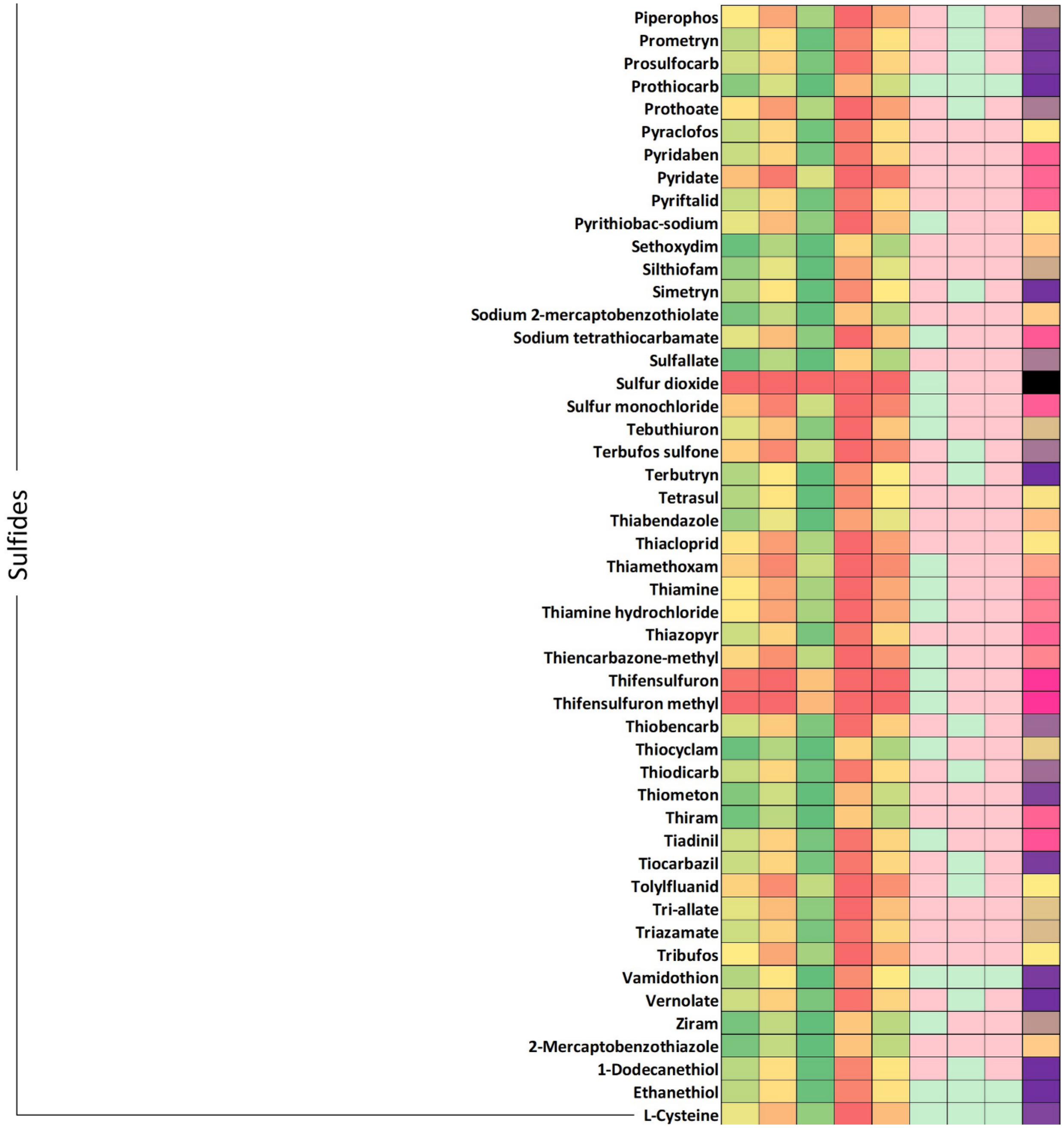

Fig. S6. Environmental performance of pesticides. Photodegradation: red indicates higher ΔG_{et}^0 values and slower reaction rates; green indicates lower ΔG_{et}^0 values and faster reaction rates. Ecotoxicity: light green = within safer-chemical space; light red = outside safer-chemical space. E_{LUMO}: purple indicates higher E_{LUMO} values and increased safety; pink indicates lower E_{LUMO} values; black indicates low E_{LUMO} values outside the safety bounds.

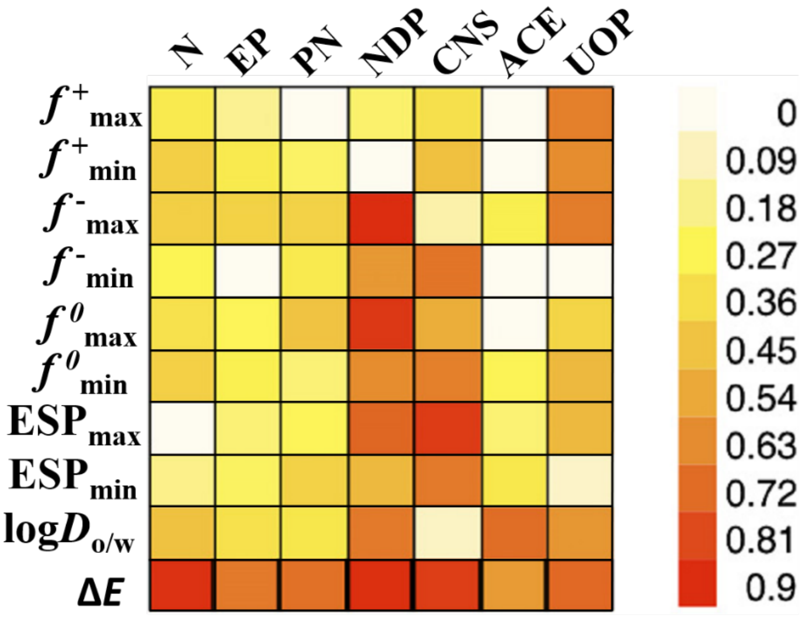

Fig. S7. Heat map of univariate correlations of electronic parameters and physicochemical properties with the log of the mean lethal concentration (log LC_{50}) by Mode of Action (MOA). Analysis of 555 compounds studied in the fathead minnow (FHM) acute aquatic toxicity dataset (21). f, Fukui index; +, −, and 0, nucleophilic, electrophilic, and radical attack, respectively; max and min, local maxima and minima in the Fukui function, respectively; ESP, most positive (max) and negative (min) atomic charge derived from the molecular electrostatic potential; Log $D_{o/w}$, octanol-water distribution coefficient at pH = 7.4; ΔE, band, i.e. HOMO-LUMO, gap. MOA categories: N = narcosis; EP = electrophiles; PN = polar narcosis; NDP = neurodepressant; CNS = central nervous system seizure or stimulant; ACE = acetylcholinesterase inhibition; UOP = uncoupler of oxidative phosphorylation.

Compound	HOMO (eV)[1]	LUMO (eV)[1]	Avg ΔG_{et}^0 (kcal/mol)[2]	Avg $\Delta G^{\ddagger}$ (kcal/mol)[3]	Avg log k[4]	Avg E_{cell}^0 (V)[5]	Log $D_{o/w}$[6]
(-)-cis-Permethrin	-0.25174	-0.03518	11.5281321	13.3948	8.744468	0.354156	5.7
({5-[2-Chloro-4-(trifluoromethyl)phenoxy]-2-nitrobenzoyl}oxy)acetic acid	-0.2854	-0.1124	32.4389447	38.48066	6.48674	-0.27317	0.86
(5-Chloroquinolin-8-yloxy)acetic acid	-0.24252	-0.06839	5.8003338	8.809721	9.157125	0.52599	-1.35
(R)-Mecoprop	-0.26126	-0.02526	17.4423014	19.16079	8.225529	0.176731	-0.41
(R)-N N-Diethyl-2-(1-naphthyloxy)propionamide	-0.23432	-0.04919	0.70619645	5.557949	9.449785	0.678814	3.21
[4-(Dimethylamino)phenyl]bis[4-(ethylamino)-3-methylphenyl]methylium acetate	-0.21196	-0.10296	-13.184646	0.642567	9.892169	1.095539	2.83
1 1'-Oxybis[2 3 4 5 6-pentabromobenzene]	-0.26643	-0.08252	20.6540928	22.73131	7.904182	0.080377	11.16
1 2-Benzisothiazolin-3-one	-0.26423	-0.0847	19.2873731	21.17415	8.044326	0.121379	1.35
1 3 6-Tri-O-galloyl-beta-D-glucopyranose	-0.24882	-0.06598	9.71412225	11.83631	8.884732	0.408576	1.59
1 3-Bis(4-nitrophenyl)urea 4 6-dimethylpyrimidin-2-ol (1:1)	-0.25316	-0.11032	12.4102876	14.18834	8.673049	0.327691	3
1 4-Bis(butylamino)anthracene-9 10-dione	-0.20199	-0.10779	-19.378372	0.314902	9.921659	1.281351	5.81
1 4-Bis(ethylamino)anthraquinone	-0.20274	-0.10803	-18.912445	0.299562	9.923039	1.267373	3.88
1 4-Bis(N-isopropylamino)anthraquinone	-0.20223	-0.10696	-19.229275	0.309285	9.922164	1.276878	4.71
1 4-Diaminoanthraquinone	-0.21064	-0.10774	-14.004678	0.533155	9.902016	1.12014	2.56
1-((4-((Dimethylphenyl)azo)dimethylphenyl)azo)-2-naphthalenol	-0.22747	-0.10556	-3.5492719	3.437713	9.640606	0.806478	9.52
1-(1 3-Benzothiazol-2-yl)-3-isopropylurea	-0.24462	-0.03953	7.10492995	9.767578	9.070918	0.486852	2.7
1-Dodecanethiol	-0.24709	0.01769	8.63938352	10.95948	8.963647	0.440818	5.61
1-Naphthol	-0.22876	-0.04704	-2.7478772	3.79552	9.608403	0.782436	2.66

1-Phenoxy-2-propanol	-0.24731	-0.0141	8.7760555	11.06906	8.953784	0.436718	1.54
2 2' 4 4'-Tetrahydroxybenzophenone	-0.25048	-0.07962	10.7453744	12.71021	8.806081	0.377639	2.51
2 2'-(2 5-Thiophenediyl)bis[5-(1 1-dimethylethyl)benzoxazole]	-0.23291	-0.09371	-0.1697467	5.077163	9.493055	0.705092	7.58
2 2-Dichloro-1-(3-methyl-2 3-dihydro-4H-1 4-benzoxazin-4-yl)ethanone	-0.247	-0.05493	8.58347226	10.91481	8.967667	0.442496	2.44
2 2'-Methylenebis(4-methyl-6-tert-butylphenol)	-0.23584	-0.01746	1.65047557	6.101998	9.40082	0.650486	7.57
2 2'-Methylenebis(ethyl-6-tert-butylphenol)	-0.23607	-0.01613	1.79335991	6.186649	9.393202	0.646199	8.46
2 3 5 6-Tetrachloroaniline	-0.2433	-0.04164	6.28489809	9.159542	9.125641	0.511453	3.56
2 4 5-Trichlorophenoxyacetic acid	-0.25729	-0.04035	14.9759934	16.62886	8.453403	0.25072	-0.4
2 4 6-Trichlorophenol	-0.2592	-0.04047	16.1625547	17.82424	8.345819	0.215123	2.14
2 4 6-Triisopropylphenol	-0.23255	-0.0062	-0.3933917	4.958094	9.503772	0.711802	5.4
2 4 6-Tris(dimethylaminomethyl)phenol	-0.22988	-0.01135	-2.0520925	4.121784	9.579039	0.761563	-2.5
2 4-D 2-EHE	-0.25307	-0.03263	12.3543764	14.13735	8.677638	0.329369	5.67
2 4-D dimethylamine salt	-0.2539	-0.03311	12.8700025	14.61112	8.635	0.3139	-0.99
2 4-D isopropyl ester	-0.25311	-0.03249	12.3792258	14.16	8.6756	0.328623	3.42
2 4-D-Butotyl	-0.25361	-0.0329	12.6898439	14.44468	8.649979	0.319305	3.92
2 4-Dichlorophenoxyacetic acid	-0.2539	-0.03311	12.8700025	14.61112	8.635	0.3139	-0.99
2 4-Dihydroxybenzophenone	-0.25314	-0.0775	12.3978629	14.177	8.67407	0.328064	2.98
2 4-Diisopropylphenol	-0.23648	-0.0095	2.04806678	6.339066	9.379484	0.638558	4.16
2 4-Dimethyl-N-phenyl-5-thiazolecarboxamide	-0.24687	-0.06615	8.50271154	10.85045	8.973459	0.444919	2.11
2 6-Di-tert-butyl-4-methoxyphenol	-0.22075	-0.00621	-7.7239796	1.885068	9.780344	0.931719	4.6
2 7 8 9-Tricyclazole	-0.24878	-0.03589	9.6892728	11.81565	8.886592	0.409322	1.47
2-((Dimethylamino)methyl)phenol	-0.23779	-0.01502	2.86188628	6.839087	9.334482	0.614143	-0.02

2-(2 4 5-Trichlorophenoxy)propionic acid	-0.25659	-0.04182	14.541128	16.20132	8.491881	0.263766	0.17
2-(2-(2-(2-Phenoxyethoxy)ethoxy)ethoxy)ethanol	-0.24569	-0.01346	7.76965275	10.27524	9.025228	0.46691	0.98
2-(2H-Benzotriazol-2-yl)-4-methylphenol	-0.25181	-0.06079	11.5716187	13.43337	8.740996	0.352851	3.18
2-(2H-Benzotriazol-2-yl)-6-dodecyl-4-methylphenol	-0.2391	-0.07694	3.67570579	7.358956	9.287694	0.589729	8.01
2-(2-Naphthyloxy)acetamide	-0.23261	-0.04928	-0.3561176	4.977835	9.501995	0.710684	1.48
2-(3-Hydroxypropyl)-6-((3-hydroxypropyl)amino)-1H-benz(de)isoquinoline-1 3(2H)-dione	-0.2221	-0.09198	-6.8853107	2.15506	9.756045	0.906559	0.12
2-(Butylthio)ethanol	-0.23819	0.02533	3.11038079	6.995721	9.320385	0.606689	1.49
2-(N N'-Dimethylureido)-5-trifluoromethyl-1 3 4-thiadiazole	-0.27067	-0.0713	23.2881346	25.89028	7.619875	0.001356	0.68
2-(Thiocyanomethylthio)benzothiazole	-0.25381	-0.0575	12.8140912	14.55936	8.639658	0.315577	3.69
2-[(4-Dodecylphenyl)azo]-4-(2 4-xylylazo)resorcinol	-0.22645	-0.10603	-4.1829329	3.168421	9.664842	0.825488	11.47
2-Anthracenesulfonic acid 1-amino-9 10-dihydro-4-[(4-methyl-3-sulfophenyl)amino]-9 10-dioxo- sodium salt (1:2)	-0.22026	-0.12645	-8.0283854	1.792286	9.788694	0.940852	-0.4
2-Chloro-N-phenylacetamide	-0.25367	-0.03439	12.7271181	14.47903	8.646887	0.318186	1.75
2-Ethylhexyl diphenyl phosphate	-0.27346	-0.02352	25.0213837	28.08237	7.422587	-0.05064	6.45
2-Ethylhexyl salicylate	-0.25933	-0.05765	16.2433154	17.90713	8.338358	0.212701	5.34
2-Hydroxy-4-methoxybenzophenone	-0.25221	-0.07727	11.8201132	13.65487	8.721062	0.345397	3.13
2-Hydroxyacetophenone	-0.28624	-0.07959	32.9607832	39.27428	6.415314	-0.28882	0.71
2-Mercaptobenzothiazole	-0.23232	-0.0528	-0.5362761	4.882807	9.510547	0.716088	2.63
2-Methyl-3(2H)-isothiazolone	-0.24923	-0.04555	9.96882912	12.04919	8.865573	0.400935	0.23
2-Methyl-4 6-dinitrophenol	-0.2827	-0.11046	30.7616068	35.98502	6.711348	-0.22285	0.17

2-Naphthalenesulfonic acid 7-(benzoylamino)-4-hydroxy-3-[2-[4-[2-(4-sulfophenyl)diazenyl]phenyl]diazenyl]- compd. with 2 2' 2''-nitrilotris[ethanol] (1:2)	-0.23984	-0.12683	4.13542062	7.661395	9.260474	0.575937	2.17
2-Naphthalenesulfonic acid 7-amino-4-hydroxy-3-[[4-[(4-sulfophenyl)azo]phenyl]azo]- compd. with 2 2' 2''-nitrilotris[ethanol] (1:2)	-0.22606	-0.11964	-4.4252151	3.068637	9.673823	0.832756	0.26
2-Naphthalenol	-0.23215	-0.04701	-0.6418862	4.827553	9.51552	0.719257	2.66
2-Naphthoxyacetic acid	-0.23241	-0.04938	-0.4803648	4.912194	9.507903	0.714411	-0.88
2-Phenoxyethanol	-0.24736	-0.01425	8.80711731	11.09405	8.951536	0.435786	1.13
2-Phenylphenol	-0.24475	-0.03132	7.18569067	9.828551	9.06543	0.484429	3.31
3 3 5-Trimethylcyclohexyl salicylate	-0.25937	-0.05872	16.2681648	17.93268	8.336059	0.211955	4.99
3 3'-(1 4-Phenylenediimino)bis(4 5 6 7-tetrachloro-1H-isoindol-1-one)	-0.24288	-0.10766	6.02397886	8.970303	9.142673	0.519281	7.85
3 3-Dimethyl-1-(methylthio)butanone-O-(N-methylcarbamoyl)oxime	-0.23487	-0.02107	1.0478764	5.751724	9.432345	0.668564	2.56
3 4-Dihydro-6-methyl-N-phenyl-2H-pyran-5-carboxamide	-0.23908	-0.04063	3.66328106	7.35087	9.288422	0.590102	1.93
3-(5-(Aminosulfonyl)benzoxazol-2-yl)-7-(diethylamino)coumarin	-0.21675	-0.08877	-10.208925	1.20885	9.841203	1.006268	2.6
3-Chloro-4-methylbenzenamine hydrochloride	-0.22704	-0.0173	-3.8164035	3.322721	9.650955	0.814492	2.26
3-Dimethylaminophenol	-0.21533	-0.00505	-11.09108	1.013301	9.858803	1.032732	1.78
3-Ethoxy-4-hydroxybenzaldehyde	-0.24723	-0.07304	8.7263566	11.02915	8.957377	0.438209	1.44
3-Hydroxycarbofuran	-0.24912	-0.02568	9.90049313	11.99188	8.87073	0.402985	1.13
4-((4 5-Dihydro-3-methoxy-4-methyl-5-oxo-	-0.26782	-0.05774	21.5176112	23.74401	7.81304	0.054472	-3.1

1H-1 2 4-triazol-1-yl)carbonylsulfamoyl)-5-methylthiophene-3-carboxylic acid							
4-(1 1 3 3-Tetramethylbutyl)phenol	-0.23711	-0.00992	2.43944563	6.577057	9.358065	0.626817	4.69
4-(2-Methylbutan-2-yl)phenol	-0.24006	-0.0113	4.2720926	7.752531	9.252272	0.571837	3.66
4-Aminobenzoic acid	-0.23701	-0.0544	2.377322	6.538974	9.361492	0.62868	-1.8
4-Chloro-3 5-dimethylphenol	-0.24937	-0.01553	10.0558022	12.12232	8.858991	0.398326	3.29
4-Chloro-3-methyl-2(3H)-benzothiazolone	-0.25107	-0.03431	11.1119038	13.02849	8.777436	0.366643	2.46
4-Chloro-3-methylphenol	-0.24515	-0.01825	7.43418517	10.01738	9.048435	0.476974	2.78
4-Chlorophenoxyacetic acid	-0.25059	-0.02646	10.8137104	12.76924	8.800768	0.375589	-1.56
4-Hydroxy-3-methoxybenzaldehyde	-0.24625	-0.07342	8.11754506	10.54622	9.00084	0.456474	1.08
4-Hydroxybenzoic acid	-0.26354	-0.05967	18.85872	20.6973	8.087243	0.134238	-1.58
4-Methoxybenzaldehyde	-0.25818	-0.07259	15.5288937	17.18062	8.403745	0.234133	1.53
4-Methoxyphenol	-0.22999	-0.01732	-1.9837566	4.15461	9.576085	0.759513	1.51
4-Nitro-3-methylphenol	-0.27084	-0.09971	23.3937447	26.02127	7.608085	-0.00181	1.73
4-Nitrophenol	-0.2728	-0.10422	24.6113678	27.55568	7.469989	-0.03834	1.12
4-Propylanisole	-0.2375	-0.0115	2.68172777	6.726685	9.344598	0.619548	3.22
4-tert-Butylphenol	-0.24091	-0.01129	4.80014342	8.109906	9.220108	0.555996	3.21
4-Thiazolidinecarboxylic acid 3-acetyl-	-0.25061	-0.02197	10.8261352	12.77999	8.799801	0.375216	-3.94
4-Tolyl acetate	-0.2651	-0.01852	19.8278486	21.78324	7.989508	0.105165	2.09
5 7-Dimethoxy-2H-chromen-2-one	-0.24427	-0.07495	6.88749726	9.604393	9.085605	0.493375	1.47
5-(((2 6-Difluorophenyl)methoxy)methyl)-4 5-dihydro-5-methyl-3-(3-methyl-2-thienyl)isoxazole	-0.23424	-0.05463	0.65649755	5.530055	9.452295	0.680305	4.59
5-(2-Chloro-4-(trifluoromethyl)phenoxy)-2-nitrobenzoic acid	-0.28475	-0.11229	32.0351411	37.87215	6.541506	-0.26105	1.02
5-Chloro-2-methyl-3(2H)-isothiazolone	-0.25651	-0.05535	14.4914291	16.15282	8.496246	0.265257	1.35
5-Hydroxythiabendazole	-0.22805	-0.0553	-3.1889549	3.596206	9.626341	0.795669	2.02

7-(Benzoylamino)-4-hydroxy-3-[[4-[(4-sulfophenyl)azo]phenyl]azo]-2-naphthalenesulfonic acid	-0.23984	-0.12683	4.13542062	7.661395	9.260474	0.575937	2.17
8-Hydroxyquinoline	-0.23962	-0.06038	3.99874864	7.57082	9.268626	0.580038	1.82
8-Hydroxyquinoline-5-sulfonic acid	-0.26129	-0.08109	17.4609384	19.18061	8.223745	0.176172	-1.41
Acephate	-0.28164	-0.02579	30.1030964	35.02829	6.797454	-0.20309	-0.98
Acetic acid (2 4-dichlorophenoxy)- compd. with methanamine (1:1)	-0.2539	-0.03311	12.8700025	14.61112	8.635	0.3139	-0.99
Acetic acid (2 4-dichlorophenoxy)- compd. with N-ethylethanamine (1:1)	-0.2539	-0.03311	12.8700025	14.61112	8.635	0.3139	-0.99
Acibenzolar-S-methyl	-0.26966	-0.09426	22.660686	25.11892	7.689297	0.020179	2.8
Acid Blue 25	-0.21633	-0.12507	-10.469844	1.148583	9.846628	1.014095	2.28
Acid Red 337	-0.25484	-0.0386	13.4539646	15.15729	8.585844	0.296381	1.66
Acid Violet 9	-0.20486	-0.09277	-17.595424	0.291385	9.923775	1.227863	1.95
Acifluorfen-sodium	-0.28475	-0.11229	32.0351411	37.87215	6.541506	-0.26105	1.02
Aclonifen	-0.24734	-0.10101	8.79469259	11.08405	8.952436	0.436159	3.84
Acrinathrin	-0.25681	-0.07398	14.6778	16.33508	8.479843	0.259666	6.46
Alanycarb	-0.24556	-0.022	7.68889204	10.21286	9.030843	0.469333	3.13
Aldicarb	-0.241	-0.02581	4.85605468	8.148235	9.216659	0.554318	1.28
Allura Red C.I.16035	-0.24717	-0.10813	8.68908242	10.99926	8.960066	0.439328	-0.97
alpha-Tocopherol	-0.21401	-0.00051	-11.911112	0.852439	9.873281	1.057333	10.51
alpha-Vitamin E	-0.21377	0.00174	-12.060209	0.825356	9.875718	1.061806	10.51
Amaranth	-0.24873	-0.11997	9.65821099	11.78984	8.888914	0.410254	-4.49
Ametryn	-0.2484	-0.02523	9.45320302	11.62026	8.904177	0.416404	2.59
Aminocarb	-0.2269	-0.0132	-3.9033766	3.285743	9.654283	0.817101	2.09
Ancymidol	-0.2421	-0.0503	5.53941457	8.62427	9.173816	0.533818	1.47
Anethole	-0.226	-0.0205	-4.4624892	3.053442	9.67519	0.833875	2.94
Anilazine	-0.2759	-0.0669	26.5372002	30.07327	7.243406	-0.09612	4.4
Anilofos	-0.26347	-0.03346	18.8152335	20.64923	8.091569	0.135543	3.6
Anisole	-0.2465	-0.0139	8.27285413	10.66836	8.989848	0.451814	1.82
Aramite	-0.2388	-0.0168	3.48933491	7.23815	9.298567	0.59532	4.23
Asulam	-0.24285	-0.04655	6.00534177	8.956864	9.143882	0.51984	-0.58
Azafenidin	-0.2496	-0.0441	10.1986865	12.24297	8.848133	0.394039	3.5
Azamethiphos	-0.2641	-0.0567	19.2066123	21.08389	8.05245	0.123802	1.52
Azinphos-ethyl	-0.2659	-0.0837	20.3248376	22.35106	7.938405	0.090255	3.96

Azinphos-methyl	-0.2673	-0.0839	21.1945684	23.36254	7.847372	0.064163	3.24
Aziprotryne	-0.2579	-0.0565	15.3549475	17.00604	8.419456	0.239352	3.07
Azoxystrobin	-0.25756	-0.06173	15.1437272	16.79528	8.438425	0.245688	4.22
Barban	-0.2536	-0.0289	12.6836316	14.43896	8.650494	0.319491	3.57
Basic Blue 7	-0.18613	-0.08168	-29.231179	2.162746	9.755353	1.576935	4.5
Beflubutamid	-0.25667	-0.04543	14.5908269	16.2499	8.487509	0.262275	4.55
Benazolin	-0.25637	-0.03716	14.404456	16.06813	8.503869	0.267866	-1.53
Bendiocarb	-0.2387	-0.0144	3.42721128	7.198112	9.30217	0.597184	1.57
Benfuracarb	-0.24	-0.0173	4.23481842	7.727621	9.254514	0.572955	3.5
Benfuresate	-0.2442	-0.0235	6.84401072	9.571926	9.088527	0.49468	2.21
Benodanil	-0.2508	-0.0386	10.9441701	12.88233	8.79059	0.371675	3.99
Bensultap	-0.2481	-0.0688	9.26683215	11.46718	8.917954	0.421995	3.28
Bentazone	-0.2698	-0.0733	22.747659	25.22514	7.679737	0.01757	-0.19
Benthiavalicarb	-0.26174	-0.04588	17.7404948	19.47927	8.196866	0.167785	-2.88
Benzenepropanoic acid 3-(2H-benzotriazol-2-yl)-5-(1 1-dimethylethyl)-4-hydroxy- methyl ester	-0.23997	-0.07726	4.21618133	7.715181	9.255634	0.573515	4.4
Benzethonium chloride	-0.23503	-0.02759	1.1472742	5.808751	9.427212	0.665582	2.37
Benzobicyclon	-0.25624	-0.09862	14.3236953	15.98968	8.510929	0.270289	4.17
Benzocaine	-0.2353	-0.0501	1.31500799	5.905657	9.418491	0.66055	1.5
Benzothiazole	-0.2597	-0.0456	16.4731728	18.14413	8.317028	0.205805	2.11
Benzovindiflupyr	-0.24943	-0.05185	10.0930764	12.15374	8.856164	0.397208	3.85
Benzoximate	-0.2496	-0.0704	10.1986865	12.24297	8.848133	0.394039	3.89
Benzthiazuron	-0.2471	-0.0428	8.64559588	10.96445	8.9632	0.440632	1.93
Benzyl salicylate	-0.26	-0.0609	16.6595437	18.33746	8.299629	0.200214	4.05
beta-Cyfluthrin	-0.25729	-0.04174	14.9759934	16.62886	8.453403	0.25072	5.59
beta-Cypermethrin	-0.25494	-0.03862	13.5160882	15.21599	8.580561	0.294517	5.44
Bifenazate	-0.2248	-0.0327	-5.2079728	2.75828	9.701755	0.856239	3.95
Bifenox	-0.2752	-0.1113	26.1023348	29.49506	7.295444	-0.08307	4.63
Binapacryl	-0.2851	-0.11621	32.2525738	38.19921	6.512071	-0.26758	4.77
Biotin	-0.24	-0.009	4.23481842	7.727621	9.254514	0.572955	-2.57
Bisphenol A	-0.2341	-0.0152	0.56952448	5.481419	9.456672	0.682914	4.04
Bisphenol A diglycidyl ether	-0.23325	-0.01493	0.04147365	5.190993	9.482811	0.698756	4.04
Bispyribac	-0.26417	-0.06627	19.2500989	21.13247	8.048078	0.122497	0.41
Bispyribac-sodium	-0.26417	-0.06627	19.2500989	21.13247	8.048078	0.122497	0.41
Bitertanol	-0.2406	-0.0352	4.60756018	7.978602	9.231926	0.561773	4.32
Boscalid	-0.2525	-0.0565	12.0002717	13.81661	8.706505	0.339992	4.92
Bromofenoxim	-0.2566	-0.1131	14.5473404	16.20739	8.491335	0.26358	3.33

Bromofos	-0.26838	-0.04555	21.8655035	24.15831	7.775752	0.044035	4.64
Bromophenol blue	-0.2617	-0.0565	17.7156453	19.45263	8.199264	0.168531	5.67
Bromophos-ethyl	-0.2678	-0.04516	21.5051865	23.72928	7.814365	0.054844	5.35
Bromothymol blue	-0.2502	-0.0526	10.5714283	12.56057	8.819549	0.382857	9.12
Bromoxynil	-0.27004	-0.06091	22.8967557	25.40775	7.663302	0.013097	1.19
Bromoxynil heptanoate	-0.2822	-0.0726	30.4509887	35.53211	6.75211	-0.21353	5.45
Bromoxynil octanoate	-0.2822	-0.0725	30.4509887	35.53211	6.75211	-0.21353	5.9
Bumetrizole	-0.2388	-0.0815	3.48933491	7.23815	9.298567	0.59532	5.33
Buprofezin	-0.2441	-0.0288	6.7818871	9.525643	9.092692	0.496543	3.87
Butamiphos	-0.26044	-0.10719	16.9328876	18.62288	8.273941	0.192013	4.04
Butocarboxim	-0.2376	-0.018	2.74385139	6.765334	9.34112	0.617684	1.33
Butralin	-0.24207	-0.11192	5.52077749	8.611102	9.175001	0.534377	4.82
Butylate	-0.2475	0.00675	8.89409039	11.16415	8.945226	0.433177	3.53
Butylated hydroxytoluene	-0.2304	-0.00345	-1.7290497	4.278196	9.564962	0.751871	5.27
Butylparaben	-0.26112	-0.05508	17.3553283	19.0684	8.233844	0.17934	2.96
C.I. Acid Black 1 disodium salt	-0.2488	-0.15759	9.70169753	11.82598	8.885662	0.408949	8.96
C.I. Acid Blue 145	-0.25949	-0.14076	16.3427132	18.00942	8.329152	0.209719	-0.4
C.I. Acid Blue 324	-0.21721	-0.12065	-9.923156	1.277198	9.835052	0.997695	1.52
C.I. Acid Blue 62	-0.21438	-0.122	-11.681255	0.895496	9.869405	1.050438	1.48
C.I. Acid Blue 74	-0.23409	-0.1365	0.56331211	5.477953	9.456984	0.683101	-3.88
C.I. Acid Blue 80	-0.21245	-0.11303	-12.880241	0.688311	9.888052	1.086407	5.09
C.I. Acid Orange 24 monosodium salt	-0.23863	-0.11269	3.38372474	7.170155	9.304686	0.598488	2.88
C.I. Acid Orange 7	-0.23685	-0.1053	2.2779242	6.478282	9.366955	0.631662	1.87
C.I. Acid Orange 8 monosodium salt	-0.23633	-0.09742	1.95488134	6.283078	9.384523	0.641354	2.38
C.I. Acid Red 1	-0.24113	-0.11515	4.9368154	8.203765	9.211661	0.551896	-2.4
C.I. Acid Red 106 disodium salt	-0.2488	-0.11689	9.70169753	11.82598	8.885662	0.408949	-0.56
C.I. Acid Red 14	-0.24613	-0.11511	8.04299671	10.48785	9.006094	0.45871	-1.53
C.I. Acid Red 17 disodium salt	-0.23712	-0.11659	2.44565799	6.580871	9.357722	0.62663	0.31
C.I. Acid Red 73 disodium salt	-0.24191	-0.11193	5.42137968	8.541046	9.181306	0.537359	1.08
C.I. Acid Red 88 monosodium salt	-0.2358	-0.11554	1.62562612	6.087339	9.40214	0.651231	2.86
C.I. Acid Violet 12 disodium salt	-0.24381	-0.13811	6.60172858	9.392076	9.104713	0.501948	-2.25
C.I. Acid Violet 19	-0.22133	-0.10746	-7.3636626	1.998482	9.770137	0.92091	-6.3

C.I. Acid Violet 34 disodium salt	-0.28078	-0.14318	29.5688332	34.26163	6.866453	-0.18706	3.03
C.I. Acid Violet 43	-0.2709	-0.13744	23.4310189	26.06759	7.603917	-0.00293	3.3
C.I. Acid Yellow 36 monosodium salt	-0.216	-0.09503	-10.674852	1.102661	9.85076	1.020246	2.62
C.I. Basic Blue 41	-0.22397	-0.12335	-5.7235989	2.56387	9.719252	0.871708	0.43
C.I. Direct Red 81 disodium salt	-0.23984	-0.12683	4.13542062	7.661395	9.260474	0.575937	2.17
C.I. Direct Violet 9 disodium salt	-0.21576	-0.11413	-10.823949	1.070055	9.853695	1.024718	2.88
C.I. Food Red 11	-0.23497	-0.11442	1.11000002	5.787331	9.42914	0.6667	-3.16
C.I. Pigment Orange 36	-0.23133	-0.11477	-1.1513	4.56573	9.539084	0.734539	3.24
C.I. Pigment Orange 5	-0.24387	-0.13656	6.63900276	9.419631	9.102233	0.50083	4.15
C.I. Pigment Red 170	-0.2345	-0.11076	0.83044371	5.628008	9.443479	0.675087	5.21
C.I. Pigment Red 23	-0.23771	-0.10903	2.81218738	6.807982	9.337282	0.615634	5.05
C.I. Pigment Red 52	-0.24154	-0.11696	5.19152227	8.380177	9.195784	0.544254	-0.16
C.I. Pigment Yellow 1	-0.25355	-0.11009	12.6525698	14.41036	8.653067	0.320423	3.98
C.I. Pigment Yellow 109	-0.25162	-0.10428	11.4535838	13.32881	8.750407	0.356392	8.36
C.I. Pigment Yellow 73	-0.24043	-0.11439	4.50195002	7.907068	9.238364	0.564941	3.91
C.I. Pigment Yellow 74	-0.2398	-0.12099	4.11057117	7.644885	9.26196	0.576683	3.15
C.I. Pigment Yellow 97	-0.23768	-0.09958	2.7935503	6.796337	9.33833	0.616193	3.49
C.I. Solvent Blue 104	-0.20784	-0.10929	-15.74414	0.367781	9.9169	1.172324	11.48
C.I. Solvent green 7	-0.24782	-0.11738	9.09288599	11.32525	8.930728	0.427213	-5.64
C.I. Solvent Orange 7	-0.22527	-0.08399	-4.9159917	2.871901	9.691529	0.84748	5.35
C.I. Solvent Red 169	-0.22747	-0.11719	-3.5492719	3.437713	9.640606	0.806478	3.81
C.I. Solvent Red 2	-0.22536	-0.09048	-4.8600805	2.893949	9.689545	0.845802	4.12
C.I. Solvent Red 23	-0.23189	-0.11396	-0.8034077	4.743694	9.523068	0.724102	6.73
C.I. Solvent Red 26	-0.2276	-0.10598	-3.4685112	3.472899	9.637439	0.804055	8.27
C.I. Solvent Red 49	-0.20364	-0.06227	-18.353332	0.289741	9.923923	1.2506	6.13
C.I. Solvent Yellow 14	-0.23258	-0.09874	-0.3747546	4.967959	9.502884	0.711243	4.32
Cadusafos	-0.26661	-0.03103	20.7659154	22.86119	7.892493	0.077023	4
Calcium oxytetracycline	-0.25212	-0.10213	11.7642019	13.60487	8.725562	0.347074	-3.63
Capsaicin	-0.2334	-0.0098	0.13465909	5.241638	9.478253	0.69596	3.75
Captafol	-0.27104	-0.05056	23.517992	26.17581	7.594177	-0.00554	3.95
Captan	-0.2701	-0.0693	22.9340299	25.45351	7.659184	0.011979	3.25
Carbaryl	-0.2422	-0.0533	5.6015382	8.66824	9.169858	0.531954	2.46
Carbetamide	-0.2489	-0.02428	9.76382115	11.8777	8.881007	0.407085	1.65
Carbofuran	-0.24167	-0.01615	5.27228298	8.436518	9.190713	0.541832	2.05
Carbophenothion	-0.245	-0.0371	7.34099973	9.946355	9.054828	0.47977	4.89
Carbosulfan	-0.2409	-0.0164	4.79393106	8.105653	9.220491	0.556182	5.16

Carboxin	-0.2214	-0.0426	-7.3201761	2.012433	9.768881	0.919605	1.51
Cartap	-0.2298	0.00015	-2.1017914	4.097998	9.58118	0.763054	-1.13
Chinomethionate	-0.26383	-0.09348	19.0388785	20.89705	8.069266	0.128834	3.86
Chlomethoxyfen	-0.2746	-0.1043	25.7295931	29.00397	7.339642	-0.07189	4.46
Chloramben	-0.2441	-0.068	6.7818871	9.525643	9.092692	0.496543	-1.34
Chlorantraniliprole	-0.27146	-0.06927	23.7789112	26.50184	7.564834	-0.01337	4.87
Chlorbenside	-0.2473	-0.042	8.76984314	11.06407	8.954234	0.436905	5.43
Chlorbromuron	-0.243	-0.0339	6.09852721	9.024163	9.137825	0.517044	2.85
Chlorbufam	-0.253	-0.02836	12.3108898	14.09776	8.681202	0.330673	3.08
Chlorfenson	-0.27415	-0.06875	25.4500368	28.63839	7.372545	-0.0635	4.4
Chlorfluazuron	-0.261	-0.0595	17.2807799	18.98939	8.240955	0.181577	6.56
Chlorhexidine diacetate	-0.22643	-0.02428	-4.1953577	3.163261	9.665306	0.825861	-0.95
Chlormephos	-0.26756	-0.02723	21.3560898	23.55288	7.830241	0.059317	2.78
Chlornitrofen	-0.2807	-0.1066	29.5191343	34.19075	6.872833	-0.18557	5.23
Chloroneb	-0.263	-0.0334	18.5232524	20.32796	8.120484	0.144302	2.87
Chlorotoluron	-0.2348	-0.0197	1.00438986	5.726867	9.434582	0.669868	2.44
Chloroxuron	-0.2364	-0.0287	1.99836788	6.309173	9.382174	0.640049	3.43
Chlorpropham	-0.25062	-0.02627	10.8323475	12.78537	8.799317	0.37503	3.21
Chromafenozide	-0.24609	-0.04395	8.01814726	10.46843	9.007842	0.459456	5.23
Cinosulfuron	-0.2674	-0.0624	21.256692	23.43565	7.840791	0.062299	0.93
Cinoxate	-0.2401	-0.0759	4.29694205	7.769162	9.250775	0.571092	2.67
Clethodim	-0.2274	-0.0524	-3.5927585	3.418848	9.642304	0.807783	4.52
Clodinafop	-0.2533	-0.0475	12.4972607	14.26784	8.665895	0.325082	-0.01
Clodinafop-propargyl	-0.2532	-0.0475	12.4351371	14.21103	8.671007	0.326946	3.86
Clomeprop	-0.2489	-0.0371	9.76382115	11.8777	8.881007	0.407085	5.02
Cloquintocet-mexyl	-0.241	-0.0675	4.85605468	8.148235	9.216659	0.554318	4.83
Cloransulam-methyl	-0.2729	-0.074	24.6734914	27.63516	7.462836	-0.0402	2.75
Clorophene	-0.2451	-0.0231	7.40312336	9.993679	9.050569	0.477906	4.36
Clothianidin	-0.2583	-0.0833	15.603442	17.25571	8.396986	0.231897	-3.11
Colchicine	-0.23851	-0.0849	3.30917639	7.12236	9.308988	0.600725	1.46
Coumaphos	-0.2598	-0.08479	16.5352964	18.20846	8.311239	0.203941	4.15
Crufomate	-0.2625	-0.02415	18.2126343	19.98898	8.150992	0.153621	3.19
Cyanofenphos	-0.26718	-0.059	21.12002	23.27495	7.855254	0.066399	4.51
Cyantraniliprole	-0.27768	-0.07191	27.6430008	31.56908	7.108783	-0.12929	4.12
Cyclanilide	-0.2527	-0.0509	12.124519	13.92872	8.696415	0.336264	-0.94
Cyclaniliprole	-0.27499	-0.06286	25.9718752	29.32271	7.310956	-0.07916	6.32
Cycloate	-0.25034	0.00937	10.6584014	12.63527	8.812825	0.380248	3.2
Cycloxydim	-0.22923	-0.05462	-2.4558961	3.930668	9.59624	0.773677	1.93
Cyhalofop-butyl	-0.2545	-0.0543	13.2427442	14.95856	8.60373	0.302718	4.83

Cypermethrin	-0.25494	-0.03862	13.5160882	15.21599	8.580561	0.294517	5.44
Cyphenothrin	-0.23975	-0.03896	4.07950936	7.624274	9.263815	0.577615	5.32
Cyprodinil	-0.2234	-0.0394	-6.0777035	2.434974	9.730852	0.882331	3.21
Cyprosulfamide	-0.26321	-0.07826	18.6537121	20.47119	8.107593	0.140389	0.86
D-alpha-Tocopheryl acetate	-0.23214	-0.0119	-0.6480986	4.824313	9.515812	0.719443	10.42
Dazomet	-0.2318	-0.0315	-0.8593189	4.714848	9.525664	0.72578	1.28
Decyl diphenyl phosphate	-0.27388	-0.02329	25.282303	28.42016	7.392186	-0.05847	7.42
Deltamethrin	-0.2589	-0.04044	15.9761838	17.63369	8.362968	0.220714	5.74
Demeton-S	-0.2369	-0.0171	2.30898601	6.497216	9.365251	0.63073	2.14
Demeton-S-methyl	-0.2369	-0.0196	2.30898601	6.497216	9.365251	0.63073	1.43
Demeton-S-methylsulphon	-0.28555	-0.02642	32.5321302	38.62178	6.474039	-0.27596	-0.43
Denatonium benzoate	-0.26352	-0.02829	18.8462953	20.68356	8.088479	0.134611	0.41
Desmedipham	-0.2437	-0.031	6.53339259	9.341668	9.10925	0.503998	3.7
Desmetryn	-0.2487	-0.0255	9.6395739	11.77437	8.890306	0.410813	2.23
Diafenthiuron	-0.22262	-0.02115	-6.5622678	2.26468	9.746179	0.896868	7.04
Dialifor	-0.26476	-0.10118	19.6166283	21.54417	8.011025	0.111501	3.82
Diallate	-0.2574	-0.0225	15.0443294	16.69655	8.44731	0.24867	3.36
Diallyl disulfide	-0.2498	-0.0358	10.3229338	12.34837	8.838647	0.390312	2.76
Dicamba	-0.2716	-0.0422	23.8658843	26.61097	7.555012	-0.01598	-0.83
Dicamba-dimethylammonium	-0.27158	-0.04216	23.8534596	26.59537	7.556417	-0.0156	-0.83
Dichlofenthion	-0.26797	-0.03673	21.6107967	23.85463	7.803084	0.051676	4.58
Dichlofluanid	-0.2776	-0.0483	27.5933019	31.50106	7.114904	-0.1278	3.22
Dichlorophen	-0.2444	-0.0281	6.96825798	9.664839	9.080164	0.490952	4.27
Dichlorprop	-0.2686	-0.0335	22.0021755	24.32207	7.761014	0.039935	-0.41
Dichlorprop-P	-0.2686	-0.0335	22.0021755	24.32207	7.761014	0.039935	-0.41
Diclofop	-0.2529	-0.0357	12.2487662	14.0413	8.686283	0.332537	1.09
Dicloran	-0.2507	-0.10519	10.8820464	12.82842	8.795443	0.373539	2.29
Diclosulam	-0.2735	-0.0725	25.0462332	28.11445	7.419699	-0.05139	3.74
Diethofencarb	-0.2203	-0.011	-8.0035359	1.799756	9.788022	0.940106	3
Difenoxuron	-0.22485	-0.02488	-5.1769109	2.770245	9.700678	0.855307	2.67
Difethialone	-0.2402	-0.07303	4.35906568	7.810818	9.247026	0.569228	8.06
Diflubenzuron	-0.2483	-0.0552	9.3910794	11.56912	8.90878	0.418268	3.6
Diflufenican	-0.2521	-0.0725	11.7517772	13.59377	8.72656	0.347447	5.11
Diflufenzopyr	-0.2454	-0.0674	7.58949424	10.13634	9.037729	0.472315	-1.35
Diflufenzopyr-sodium	-0.24539	-0.06741	7.58328188	10.13157	9.038158	0.472502	-1.35
Dimepiperate	-0.248	-0.0188	9.20470852	11.41639	8.922525	0.423859	3.75
Dimethametryn	-0.2476	-0.0252	8.95621401	11.21437	8.940707	0.431314	3.47

Dimethenamid	-0.2468	-0.0204	8.459225	10.81588	8.976571	0.446223	2.92
Dimethenamid OA	-0.2498	-0.04454	10.3229338	12.34837	8.838647	0.390312	-1.12
Dimethenamide ESA	-0.24757	-0.02214	8.93757693	11.19929	8.942064	0.431873	-0.79
Dimethenamid-P	-0.24686	-0.02	8.49649918	10.84551	8.973904	0.445105	2.92
Dimethoate	-0.26698	-0.02758	20.9957728	23.12934	7.868359	0.070127	0.34
Dimethomorph	-0.24531	-0.05859	7.53358297	10.09344	9.041591	0.473993	3.28
Dimethyl (p-methoxybenzylidene)malonate	-0.24527	-0.08223	7.50873352	10.07439	9.043304	0.474738	2.32
Dimexano	-0.256	-0.0989	14.1745986	15.84538	8.523916	0.274762	3.74
Dimoxystrobin	-0.23991	-0.05419	4.17890716	7.690333	9.25787	0.574633	3.89
Dinitramine	-0.24707	-0.10632	8.6269588	10.94954	8.964541	0.441191	3.37
Dinobuton	-0.2998	-0.1181	41.3847469	53.21479	5.160669	-0.54154	4.54
Dinoseb	-0.2805	-0.1101	29.394887	34.01387	6.888752	-0.18185	1.38
Dinoterb	-0.2789	-0.1191	28.400909	32.61547	7.014608	-0.15203	1.28
Dioxabenzofos	-0.26523	-0.02381	19.9086093	21.87501	7.981249	0.102742	2.41
Dioxacarb	-0.2754	-0.0237	26.2265821	29.65969	7.280628	-0.0868	1.45
Dioxathion	-0.26517	-0.02841	19.8713351	21.83263	7.985063	0.10386	4.38
Diphenyl phosphite	-0.28366	-0.12724	31.3579936	36.8627	6.632357	-0.24074	3.37
Diphenylamine	-0.2094	-0.0204	-14.775011	0.448731	9.909614	1.14325	3.41
Diphenylurea	-0.2444	-0.0297	6.96825798	9.664839	9.080164	0.490952	2.4
Dipropetryn	-0.2483	-0.0253	9.3910794	11.56912	8.90878	0.418268	3.26
Dipyrithione	-0.2528	-0.0912	12.1866426	13.98495	8.691354	0.334401	-0.44
Disodium 4 4'-bis-(2-sulfostyryl)biphenyl	-0.22552	-0.08501	-4.7606827	2.933378	9.685996	0.84282	1.91
Disulfoton	-0.23444	-0.01985	0.78074481	5.599929	9.446006	0.676578	3.03
Disulfoton sulfone	-0.26524	-0.02507	19.9148217	21.88208	7.980613	0.102555	1.17
Dithianon	-0.2431	-0.151	6.16065084	9.069174	9.133774	0.51518	2.07
Dithiopyr	-0.28109	-0.08303	29.7614164	34.537	6.84167	-0.19284	5.77
Diuron	-0.2397	-0.0293	4.04844754	7.603691	9.265668	0.578547	2.53
DL-Methionine	-0.23161	-0.02032	-0.9773538	4.654258	9.531117	0.729321	-2.19
Drazoxolon	-0.2435	-0.0897	6.40914534	9.250374	9.117466	0.507726	1.85
Edifenphos	-0.27639	-0.06384	26.841606	30.48138	7.206676	-0.10525	4.74
Entsufon sodium	-0.23418	-0.00822	0.61922338	5.509183	9.454174	0.681423	1.39
Eosin	-0.23888	-0.11599	3.53903381	7.270263	9.295676	0.593829	0.8
EPN	-0.26851	-0.1078	21.9462643	24.25501	7.767049	0.041612	4.65
EPTC	-0.25177	0.01039	11.5467692	13.41133	8.742981	0.353597	2.8
Esfenvalerate	-0.25815	-0.0416	15.5102566	17.16187	8.405432	0.234692	6.61
Esprocarb	-0.25066	-0.01977	10.857197	12.80688	8.797381	0.374284	4.43

Ethaboxam	-0.23061	-0.05809	-1.5985901	4.342248	9.559198	0.747958	2.57
Ethanethiol	-0.2477	0.01771	9.01833764	11.2647	8.936177	0.42945	1.09
Ethidimuron	-0.2715	-0.0764	23.8037607	26.533	7.56203	-0.01411	-0.54
Ethiofencarb	-0.2324	-0.0228	-0.4865772	4.908924	9.508197	0.714597	2.59
Ethiolate	-0.25241	0.01075	11.9443604	13.76631	8.711032	0.341669	1.76
Ethion	-0.2638	-0.0313	19.0202415	20.87634	8.07113	0.129393	3.93
Ethoate-methyl	-0.2288	-0.0319	-2.7230277	3.806923	9.607377	0.781691	0.7
Ethofumesate	-0.266	-0.0273	20.3869612	22.42255	7.93197	0.088391	2.34
Ethoprop	-0.2506	-0.0245	10.8199228	12.77462	8.800285	0.375402	3.22
Ethoxyquin	-0.2735	-0.0292	25.0462332	28.11445	7.419699	-0.05139	3
Ethoxysulfuron	-0.25457	-0.05091	13.2862308	14.99936	8.600057	0.301413	1.4
Ethyl anthranilate	-0.2265	-0.0538	-4.1518711	3.181341	9.663679	0.824556	2.15
Ethylparaben	-0.2611	-0.0552	17.3429036	19.05522	8.23503	0.179713	2
Etofenprox	-0.23596	-0.02539	1.72502392	6.146088	9.396852	0.648249	6.3
Etoxazole	-0.24027	-0.04485	4.40255221	7.840047	9.244396	0.567923	5.62
Etridiazole	-0.2881	-0.0628	34.1162826	41.06063	6.254544	-0.32349	3.24
Eugenol	-0.24341	-0.01591	6.35323408	9.209442	9.12115	0.509403	2.61
Famoxadone	-0.24864	-0.05131	9.60229973	11.74347	8.893088	0.411931	5.25
Fenamidone	-0.2431	-0.036	6.16065084	9.069174	9.133774	0.51518	4.74
Fenamiphos	-0.2412	-0.0252	4.98030194	8.233746	9.208963	0.550591	3.31
Fenamiphos sulfone	-0.27654	-0.04391	26.9347914	30.60687	7.195382	-0.10804	1.53
Fenamiphos sulfoxide	-0.2565	-0.03165	14.5038538	16.16494	8.495155	0.264884	1.42
Fenazaflor	-0.2746	-0.0811	25.7295931	29.00397	7.339642	-0.07189	5.32
Fenchlorphos	-0.26989	-0.04457	22.8035703	25.29354	7.673581	0.015893	4.47
Fenfuram	-0.2411	-0.0465	4.91817831	8.190933	9.212816	0.552455	2.4
Fenhexamid	-0.2422	-0.0362	5.6015382	8.66824	9.169858	0.531954	4.49
Fenitrothion	-0.276	-0.0996	26.5993239	30.15633	7.23593	-0.09798	3.12
Fenobucarb	-0.26661	-0.01531	20.7659154	22.86119	7.892493	0.077023	3.16
Fenothiocarb	-0.24582	-0.01348	7.85041347	10.33783	9.019596	0.464488	2.91
Fenoxaprop	-0.2537	-0.0361	12.7457552	14.49623	8.64534	0.317627	0.57
Fenoxaprop-ethyl	-0.2537	-0.036	12.7457552	14.49623	8.64534	0.317627	4.5
Fenoxaprop-P	-0.25363	-0.03603	12.7022687	14.45613	8.648949	0.318932	0.57
Fenoxaprop-P-ethyl	-0.2536	-0.036	12.6836316	14.43896	8.650494	0.319491	4.5
Fenoxycarb	-0.24654	-0.02482	8.29770358	10.68797	8.988083	0.451069	3.31
Fenpropathrin	-0.2563	-0.0365	14.3609695	16.02586	8.507672	0.269171	4.85
Fenpyrazamine	-0.23782	-0.04389	2.88052337	6.85077	9.333431	0.613584	3.58
Fenpyroximate	-0.2414	-0.0607	5.10454919	8.31972	9.201225	0.546864	5
Fenson	-0.27379	-0.06333	25.2263917	28.3476	7.398716	-0.05679	3.79
Fensulfothion	-0.2625	-0.03839	18.2126343	19.98898	8.150992	0.153621	2.11

Fenthion	-0.2419	-0.0317	5.41516732	8.536678	9.181699	0.537545	3.8
Fenuron	-0.2361	-0.01604	1.811997	6.197736	9.392204	0.64564	1.32
Fipronil sulfide	-0.25963	-0.07186	16.4296862	18.09917	8.321075	0.207109	5.62
Flocoumafen	-0.2392	-0.072	3.73782941	7.399456	9.284049	0.587865	6.66
Florasulam	-0.27211	-0.0699	24.1827148	27.01044	7.51906	-0.02548	2.07
Florpyrauxifen benzyl	-0.26248	-0.05637	18.2002096	19.97548	8.152207	0.153994	5.21
Fluazifop	-0.2522	-0.0451	11.8139008	13.64931	8.721562	0.345583	0.13
Fluazifop-butyl	-0.2514	-0.04497	11.3169118	13.20826	8.761256	0.360493	5.09
Fluazifop-P	-0.2522	-0.04504	11.8139008	13.64931	8.721562	0.345583	0.13
Fluazifop-P-butyl	-0.2516	-0.045	11.4411591	13.31783	8.751395	0.356765	5.09
Fluazinam	-0.269	-0.1258	22.25067	24.62124	7.734088	0.03248	6.34
Flubendiamide	-0.25467	-0.05676	13.3483544	15.05776	8.594802	0.299549	4.98
Flubenzimine	-0.2592	-0.1214	16.1625547	17.82424	8.345819	0.215123	8.1
Flucarbazone-sodium	-0.26813	-0.0648	21.7101945	23.97291	7.792438	0.048694	1.63
Flucythrinate	-0.24896	-0.04049	9.80109533	11.90878	8.87821	0.405967	6.77
Fludioxonil	-0.24752	-0.0413	8.90651511	11.17419	8.944323	0.432805	3.57
Fluensulfone	-0.28165	-0.08272	30.1093087	35.03726	6.796647	-0.20328	1.82
Flufenacet	-0.27759	-0.07068	27.5870895	31.49257	7.115669	-0.12761	3.22
Flufenoxuron	-0.2569	-0.0575	14.7337113	16.38996	8.474904	0.257989	6.12
Flumethrin	-0.23948	-0.05632	3.91177557	7.513472	9.273788	0.582647	7.19
Flumetsulam	-0.2846	-0.0768	31.9419557	37.73242	6.554082	-0.25826	1.78
Flumiclorac-pentyl	-0.2586	-0.1047	15.7898129	17.44418	8.380024	0.226306	4.44
Flumioxazin	-0.25141	-0.10402	11.3231242	13.21373	8.760764	0.360306	1.48
Fluometuron	-0.2429	-0.0376	6.03640358	8.979268	9.141866	0.518908	2.2
Fluorescein	-0.2496	-0.0655	10.1986865	12.24297	8.848133	0.394039	3.86
Fluorescein sodium	-0.24956	-0.06546	10.1738371	12.22194	8.850025	0.394785	-1.3
Fluorodifen	-0.27929	-0.11359	28.6431912	32.9536	6.984176	-0.1593	4.23
Fluoxastrobin	-0.2599	-0.06102	16.59742	18.2729	8.305439	0.202077	5.22
Flurazole	-0.27499	-0.10103	25.9718752	29.32271	7.310956	-0.07916	4.67
Flurprimidol	-0.2666	-0.0498	20.759703	22.85396	7.893143	0.077209	3.53
Flusulfamide	-0.28739	-0.1164	33.6752049	40.37402	6.316338	-0.31026	4.45
Fluthiacet-methyl	-0.24721	-0.04682	8.71393187	11.01918	8.958274	0.438582	3.51
Flutianil	-0.2397	-0.04226	4.04844754	7.603691	9.265668	0.578547	5.61
Flutolanil	-0.2425	-0.0538	5.78790908	8.800844	9.157924	0.526363	4.56
Fluvalinate	-0.2432	-0.0439	6.22277446	9.1143	9.129713	0.513317	6.98
Folic acid	-0.23136	-0.0856	-1.1326629	4.575172	9.538235	0.73398	-7.44
Folpet	-0.29888	-0.1057	40.8132095	52.20172	5.251845	-0.5244	3.78
Fomesafen	-0.2857	-0.1127	32.6253156	38.76316	6.461315	-0.27876	2.29
Fonofos	-0.24987	-0.04031	10.3664203	12.38537	8.835316	0.389007	3.59

Fonofos oxon	-0.2767	-0.0365	27.0341892	30.74101	7.183309	-0.11103	2.77
Foramsulfuron	-0.26106	-0.06852	17.3180541	19.02887	8.237401	0.180458	-0.35
Forchlorfenuron	-0.2417	-0.0459	5.29092007	8.449548	9.189541	0.541272	2.73
Formetanate	-0.221	-0.0241	-7.5686706	1.933477	9.775987	0.92706	-0.25
Formetanate hydrochloride	-0.22096	-0.02406	-7.59352	1.925683	9.776689	0.927806	-0.25
Formothion	-0.2665	-0.0579	20.6975794	22.78177	7.89964	0.079073	0.28
Fosthiazate	-0.2695	-0.0265	22.5612882	24.99781	7.700197	0.023161	2.44
Fosthietan	-0.2796	-0.0517	28.8357744	33.22362	6.959874	-0.16507	1.88
Furathiocarb	-0.2406	-0.0214	4.60756018	7.978602	9.231926	0.561773	4.22
gamma-Cyhalothrin	-0.24844	-0.04322	9.47805247	11.64074	8.902333	0.415658	5.68
Halauxifen-methyl [ISO]	-0.25683	-0.05649	14.6902247	16.34727	8.478746	0.259293	3.34
Halfenprox	-0.2485	-0.0427	9.51532665	11.67151	8.899564	0.41454	7.97
Haloxyfop	-0.2536	-0.053	12.6836316	14.43896	8.650494	0.319491	0.73
Haloxyfop-P	-0.25356	-0.05298	12.6587821	14.41608	8.652553	0.320237	0.73
Harmine hydrochloride	-0.22997	-0.04308	-1.9961813	4.148632	9.576623	0.759885	1.82
Hexachlorophene	-0.2572	-0.044	14.9200821	16.57357	8.458379	0.252398	5.18
Hexaflumuron	-0.2651	-0.0616	19.8278486	21.78324	7.989508	0.105165	5.36
Hexyl salicylate	-0.2596	-0.0593	16.4110492	18.07992	8.322807	0.207669	4.53
Hexythiazox	-0.26348	-0.04277	18.8214459	20.6561	8.090951	0.135357	4.59
Hydroquinone	-0.2329	-0.0187	-0.175959	5.073835	9.493355	0.705279	1.36
Imibenconazole	-0.2531	-0.0345	12.3730135	14.15434	8.67611	0.32881	5.69
Imicyafos	-0.26268	-0.02582	18.3244568	20.11068	8.140039	0.150266	1.77
Indoxacarb	-0.2503	-0.0723	10.6335519	12.61391	8.814748	0.380993	5.39
Iodenphos	-0.25606	-0.04293	14.2118728	15.88139	8.520675	0.273644	4.8
Ioxynil	-0.25343	-0.05331	12.5780214	14.34186	8.659232	0.322659	1.76
Iprobenfos	-0.2721	-0.0306	24.1765024	27.00258	7.519768	-0.0253	3.88
Isoamyl salicylate	-0.2596	-0.0592	16.4110492	18.07992	8.322807	0.207669	3.93
Isocarbophos	-0.2633	-0.0614	18.7096233	20.53273	8.102054	0.138711	2.49
Isofenphos	-0.2583	-0.06	15.603442	17.25571	8.396986	0.231897	3.84
Isofetamid	-0.24918	-0.06284	9.93776731	12.02312	8.867919	0.401867	4.84
Isoprocarb	-0.2676	-0.0152	21.3809393	23.58223	7.827599	0.058572	2.72
Isoprothiolane	-0.25295	-0.06917	12.279828	14.06952	8.683744	0.331605	3.22
Isoproturon	-0.2297	-0.0131	-2.1639151	4.06837	9.583847	0.764917	2.57
Isotianil	-0.26761	-0.1015	21.3871516	23.58957	7.826938	0.058385	0.65
Isoxaben	-0.2493	-0.0453	10.0123157	12.08573	8.862284	0.399631	4.02
Karbutilate	-0.2388	-0.0193	3.48933491	7.23815	9.298567	0.59532	1.88
Kresoxim-methyl	-0.2423	-0.0696	5.66366183	8.712326	9.165891	0.53009	4.34
Lactofen	-0.28538	-0.11214	32.42652	38.46187	6.488432	-0.2728	5.45

L-Cysteine	-0.2573	-0.0236	14.9822058	16.635	8.45285	0.250534	-2.8
Lemax	-0.2475	-0.0744	8.89409039	11.16415	8.945226	0.433177	-0.76
Leptophos	-0.26391	-0.05176	19.0885774	20.95232	8.064291	0.127343	6.18
Linuron	-0.2443	-0.0328	6.90613435	9.618325	9.084351	0.492816	2.68
L-Methionine	-0.2316	-0.0203	-0.9835662	4.651081	9.531403	0.729507	-2.19
Lufenuron	-0.2631	-0.0627	18.5853761	20.3961	8.114351	0.142439	6.11
Malaoxon	-0.2885	-0.0445	34.3647771	41.45001	6.219499	-0.33094	0.97
Malathion	-0.2677	-0.0274	21.4430629	23.6557	7.820987	0.056708	1.86
Mandestrobin	-0.23806	-0.03192	3.02962007	6.944612	9.324985	0.609111	3.55
Mandipropamid	-0.2302	-0.03392	-1.8532969	4.217667	9.57041	0.755599	3.71
MCPA	-0.2462	-0.023	8.08648325	10.52188	9.003031	0.457406	-1
MCPA dimethylamine salt	-0.24618	-0.02295	8.07405852	10.51215	9.003907	0.457778	-1
MCPB	-0.24	-0.0164	4.23481842	7.727621	9.254514	0.572955	-0.19
m-Cresol	-0.2469	-0.0101	8.52134863	10.86529	8.972124	0.44436	2.18
Mecarbam	-0.2655	-0.0394	20.0763431	22.06623	7.96404	0.09771	2.02
Mecoprop	-0.2455	-0.0274	7.65161786	10.18413	9.033428	0.470451	-0.41
Mefenacet	-0.2546	-0.0321	13.3048678	15.01687	8.598482	0.300854	3.42
Mefentrifluconazole	-0.25836	-0.04081	15.6407162	17.29332	8.393601	0.230779	4.07
Mefluidide	-0.24252	-0.03708	5.8003338	8.809721	9.157125	0.52599	1.78
Mephosfolan	-0.2707	-0.0558	23.3067717	25.91337	7.617796	0.000797	2.2
Mepronil	-0.2404	-0.048	4.48331293	7.894479	9.239497	0.565501	4.19
Meptyldinocap	-0.2952	-0.11659	38.5270601	48.24736	5.607738	-0.45581	6.3
Merphos	-0.2285	-0.018	-2.9093986	3.721855	9.615033	0.787282	5.77
Metaflumizone	-0.23931	-0.06282	3.8061654	7.44414	9.280027	0.585815	6.86
Metamifop	-0.25274	-0.03686	12.1493684	13.9512	8.694392	0.335519	5.44
Methabenzthiazuron	-0.2645	-0.0243	19.4551068	21.36224	8.027398	0.116347	1.79
Methamidophos	-0.2439	-0.0399	6.65763984	9.433424	9.100992	0.500271	-0.32
Methfuroxam	-0.2756	-0.0137	26.3508294	29.82477	7.265771	-0.09052	3.12
Methidathion	-0.2341	-0.0439	0.56952448	5.481419	9.456672	0.682914	2.29
Methiocarb	-0.2587	-0.033	15.8519365	17.50723	8.374349	0.224442	3.13
Methomyl	-0.2391	-0.027	3.67570579	7.358956	9.287694	0.589729	0.72
Methoprotryne	-0.2516	-0.025	11.4411591	13.31783	8.751395	0.356765	2.24
Methoxychlor	-0.2486	-0.0246	9.57745028	11.72288	8.89494	0.412676	4.93
Methoxyfenozide	-0.24682	-0.04516	8.47164973	10.82575	8.975682	0.445851	4.75
Methyl (RS)-2-[4-(2 4-dichlorophenoxy)phenoxy] propionate	-0.25232	-0.03572	11.8884492	13.71611	8.71555	0.343347	4.72
Methyl 2-aminobenzoate	-0.2268	-0.0545	-3.9655002	3.259469	9.656648	0.818965	1.8
Methyl 3-methylorsellinate	-0.23937	-0.05338	3.84343958	7.468572	9.277829	0.584697	3.04

Methyl 4-(((2 5-dichlorophenyl)amino)carbonyl)-2-((2-hydroxy-3-(((2-methoxyphenyl)amino)carbonyl)-1-naphthyl)azo)benzoate	-0.23548	-0.10945	1.42683052	5.970729	9.412634	0.657195	8.3
Methyl disulfide	-0.2468	-0.0173	8.459225	10.81588	8.976571	0.446223	1.35
Methyl parathion	-0.2769	-0.1093	27.1584365	30.9091	7.168181	-0.11475	2.6
Methyl salicylate	-0.2601	-0.0604	16.7216673	18.40213	8.293808	0.19835	2.32
Methylene bis(thiocyanate)	-0.2925	-0.0606	36.8497222	45.44567	5.859889	-0.40549	1.16
Methylene blue	-0.22182	-0.12563	-7.0592568	2.097329	9.76124	0.911778	2.61
Methyleugenol	-0.2389	-0.0141	3.55145853	7.278303	9.294953	0.593456	2.76
Methylparaben	-0.2616	-0.0559	17.6535217	19.3861	8.205251	0.170394	1.64
Metobromuron	-0.2385	-0.02847	3.30296403	7.118385	9.309345	0.600911	2.24
Metolcarb	-0.2677	-0.0171	21.4430629	23.6557	7.820987	0.056708	1.98
Metominostrobin	-0.24927	-0.06182	9.99367857	12.07006	8.863694	0.40019	0.5
Metosulam	-0.2692	-0.0622	22.3749173	24.77152	7.720563	0.028752	3.45
Metoxuron	-0.2371	-0.0238	2.43323326	6.573243	9.358408	0.627003	1.77
Metrafenone	-0.245	-0.07525	7.34099973	9.946355	9.054828	0.47977	4.6
Metribuzin	-0.2553	-0.0697	13.7397332	15.42829	8.561454	0.287808	1.96
Molinate	-0.2532	0.00904	12.4351371	14.21103	8.671007	0.326946	2.34
Monalide	-0.2441	-0.0324	6.7818871	9.525643	9.092692	0.496543	4.5
Monolinuron	-0.23982	-0.02714	4.1229959	7.653138	9.261218	0.57631	2.08
Monuron	-0.2352	-0.0223	1.25288437	5.869668	9.42173	0.662413	1.93
Morin	-0.23843	-0.08104	3.25947749	7.090589	9.311847	0.602216	0.92
Musk ambrette	-0.2758	-0.1064	26.4750766	29.99032	7.250871	-0.09425	3.75
N N'-Disalicylidene-1 2-diaminopropane	-0.2452	-0.05763	7.46524699	10.04112	9.046299	0.476043	3.44
Napropamide	-0.2343	-0.0492	0.69377173	5.550969	9.450413	0.679187	3.21
Naptalam	-0.23156	-0.0709	-1.0084156	4.638383	9.532546	0.730252	0.23
Neburon	-0.2382	-0.0288	3.11659315	6.99966	9.320031	0.606502	3.86
Nigrosine	-0.2483	-0.0722	9.3910794	11.56912	8.90878	0.418268	-0.12
Nithiazine	-0.24858	-0.07478	9.56502555	11.7126	8.895866	0.413049	0.7
Nitrofen	-0.274	-0.106	25.3568513	28.51705	7.383466	-0.06071	4.62
Nitrofluorofen	-0.27928	-0.10628	28.6369788	32.9449	6.984959	-0.15911	4.9
Novaluron	-0.2601	-0.0581	16.7216673	18.40213	8.293808	0.19835	6.23
Noviflumuron	-0.2687	-0.0651	22.0642991	24.39669	7.754298	0.038071	6.25
o-Aminoazotoluene	-0.23263	-0.07151	-0.3436928	4.984424	9.501402	0.710311	3.98
o-Cresol	-0.24561	-0.01012	7.71995385	10.23683	9.028685	0.468401	2.18
Octabenzone	-0.25152	-0.07708	11.3914602	13.27395	8.755345	0.358256	6.23

Octhilinone	-0.24786	-0.04504	9.11773544	11.34547	8.928908	0.426468	3.33
Octinoxate	-0.23902	-0.07414	3.62600689	7.326639	9.290602	0.59122	5.38
Octrizole	-0.2472	-0.0604	8.70771951	11.0142	8.958722	0.438768	5.9
Octyl salicylate	-0.2596	-0.0595	16.4110492	18.07992	8.322807	0.207669	5.42
Omethoate	-0.2758	-0.0274	26.4750766	29.99032	7.250871	-0.09425	-0.55
Orbencarb	-0.25536	-0.0275	13.7770074	15.46382	8.558256	0.28669	3.73
Orthosulfamuron	-0.26309	-0.05066	18.5791637	20.38928	8.114965	0.142625	-0.23
Oxadiargyl	-0.2603	-0.0483	16.8459145	18.53182	8.282136	0.194623	4.77
Oxadiazon	-0.25376	-0.04521	12.7830294	14.53065	8.642242	0.316509	5.31
Oxamyl	-0.25774	-0.03622	15.2555497	16.90669	8.428398	0.242334	0.32
Oxamyl oxime	-0.251	-0.0255	11.0684173	12.99051	8.780854	0.367947	0.14
Oxathiapiprolin	-0.24359	-0.05475	6.4650566	9.291399	9.113774	0.506048	4.37
Oxycarboxin	-0.2518	-0.053	11.5654063	13.42786	8.741493	0.353038	0.17
Oxydemeton-methyl	-0.2622	-0.0246	18.0262634	19.78698	8.169172	0.159212	-0.54
Oxydisulfoton	-0.2626	-0.0236	18.2747579	20.05654	8.144911	0.151757	1.07
Oxyfluorfen	-0.2666	-0.1012	20.759703	22.85396	7.893143	0.077209	5.09
Oxytetracycline	-0.25212	-0.10213	11.7642019	13.60487	8.725562	0.347074	-6.1
Oxytetracycline hydrochloride	-0.25212	-0.10213	11.7642019	13.60487	8.725562	0.347074	-6.1
p p'-Methoxychlor olefin	-0.22939	-0.04364	-2.3564983	3.977258	9.592047	0.770695	4.58
Parathion	-0.2751	-0.1092	26.0402112	29.41293	7.302837	-0.08121	3.32
Pebulate	-0.25181	0.01044	11.5716187	13.43337	8.740996	0.352851	3.25
Pencycuron	-0.2374	-0.026	2.61960414	6.688151	9.348066	0.621412	5.01
Pendimethalin	-0.236	-0.11	1.74987337	6.160821	9.395526	0.647504	4.82
Penoxsulam	-0.25951	-0.07284	16.3551379	18.02223	8.327999	0.209346	2.04
Pentachloroaniline	-0.2857	-0.0914	32.6253156	38.76316	6.461315	-0.27876	3.75
Pentachloroanisole	-0.2766	-0.0528	26.9720656	30.65713	7.190858	-0.10916	4.84
Pentachlorophenol	-0.2666	-0.0536	20.759703	22.85396	7.893143	0.077209	2.79
Pentachlorothioanisole	-0.2511	-0.0618	11.1305409	13.04478	8.77597	0.366084	5.62
Pentanochlor	-0.2455	-0.0305	7.65161786	10.18413	9.033428	0.470451	4.46
Penthiopyrad	-0.24251	-0.04685	5.79412144	8.805282	9.157525	0.526176	5.09
Pentoxazone	-0.25478	-0.07527	13.4166904	15.12212	8.589009	0.297499	4.26
Pentyl 2-hydroxybenzoate	-0.2569	-0.0641	14.7337113	16.38996	8.474904	0.257989	4.09
Perfluidone	-0.2776	-0.0662	27.5933019	31.50106	7.114904	-0.1278	2.98
Permethrin	-0.25071	-0.03729	10.8882588	12.8338	8.794958	0.373352	5.7
Phenmedipham	-0.2421	-0.0295	5.53941457	8.62427	9.173816	0.533818	3.86
Phenol	-0.2511	-0.0152	11.1305409	13.04478	8.77597	0.366084	1.67
Phenol red	-0.25384	-0.05091	12.8327283	14.5766	8.638106	0.315018	4.1
Phenothrin	-0.23822	-0.02907	3.12901788	7.007543	9.319321	0.606129	5.57
Phenthoate	-0.2655	-0.0384	20.0763431	22.06623	7.96404	0.09771	3.36

Phenyl didecyl phosphite	-0.26671	-0.01945	20.828039	22.93351	7.885984	0.075159	9.76
Phorate	-0.245	-0.0189	7.34099973	9.946355	9.054828	0.47977	3.16
Phorate oxon	-0.24846	-0.01908	9.4904772	11.651	8.90141	0.415286	2.27
Phorate sulfone	-0.2679	-0.0277	21.5673101	23.80297	7.807733	0.052981	1.31
Phorate sulfoxide	-0.262	-0.0239	17.9020162	19.65289	8.18124	0.16294	1.2
Phosalone	-0.2525	-0.0351	12.0002717	13.81661	8.706505	0.339992	3.75
Phosmet	-0.26726	-0.10254	21.1697189	23.33332	7.850001	0.064908	2.15
Picolinafen	-0.2475	-0.0739	8.89409039	11.16415	8.945226	0.433177	5.35
Picric acid	-0.30623	-0.13292	45.3792961	60.56854	4.498832	-0.66138	-0.32
Pigment Yellow 65	-0.24005	-0.109	4.26588024	7.748377	9.252646	0.572024	3.15
Piperine	-0.22202	-0.07721	-6.9350096	2.138473	9.757537	0.90805	2.78
Piperonal	-0.2448	-0.0751	7.21675248	9.852054	9.063315	0.483497	1.31
Piperophos	-0.26146	-0.03048	17.5665486	19.29316	8.213615	0.173004	3.59
Poly(oxy-1 2-ethanediyl) alpha-[4-(1 1-dimethylethyl)phenyl]-omega-hydroxy- phosphate	-0.23824	-0.01	3.1414426	7.01543	9.318611	0.605757	2.67
Ponceau MX	-0.24477	-0.11215	7.19811539	9.837948	9.064585	0.484057	-1.26
Procaine hydrochloride	-0.23545	-0.0507	1.40819343	5.959858	9.413613	0.657754	0.31
Prochloraz	-0.2603	-0.0397	16.8459145	18.53182	8.282136	0.194623	3.62
Prodiamine	-0.2427	-0.1037	5.91215633	8.889825	9.149916	0.522635	4.42
Profenofos	-0.26157	-0.03708	17.6348846	19.36617	8.207045	0.170953	4.88
Profoxydim	-0.22833	-0.0543	-3.0150088	3.674113	9.61933	0.79045	3.98
Promecarb	-0.2611	-0.0126	17.3429036	19.05522	8.23503	0.179713	3.23
Prometryn	-0.2477	-0.021	9.01833764	11.2647	8.936177	0.42945	3
Propanil	-0.2493	-0.0375	10.0123157	12.08573	8.862284	0.399631	3.12
Propaphos	-0.2439	-0.0309	6.65763984	9.433424	9.100992	0.500271	4.16
Propaquizafop	-0.25546	-0.08137	13.839131	15.52312	8.552919	0.284826	4.36
Propham	-0.2455	-0.0196	7.65161786	10.18413	9.033428	0.470451	2.6
Propoxur	-0.2477	-0.0162	9.01833764	11.2647	8.936177	0.42945	2.09
Propyl gallate	-0.2503	-0.0569	10.6335519	12.61391	8.814748	0.380993	1.87
Propylparaben	-0.26112	-0.05514	17.3553283	19.0684	8.233844	0.17934	2.52
Prosulfocarb	-0.2514	-0.0204	11.3169118	13.20826	8.761256	0.360493	4.17
Prothiocarb	-0.23626	0.00309	1.9113948	6.25704	9.386866	0.642658	-0.99
Prothiofos	-0.2611	-0.0409	17.3429036	19.05522	8.23503	0.179713	5.6
Prothoate	-0.2638	-0.0296	19.0202415	20.87634	8.07113	0.129393	1.83
Pyraclofos	-0.2493	-0.0474	10.0123157	12.08573	8.862284	0.399631	4.19
Pyraflufen-ethyl	-0.2541	-0.0436	12.9942497	14.72647	8.624618	0.310173	4.47
Pyridaben	-0.25035	-0.07846	10.6646137	12.64062	8.812344	0.380062	5.2

Pyridalyl	-0.2541	-0.0411	12.9942497	14.72647	8.624618	0.310173	6.31
Pyridate	-0.2722	-0.0726	24.2386261	27.08125	7.512688	-0.02716	6.61
Pyriftalid	-0.24985	-0.07197	10.3539956	12.37479	8.836268	0.38938	3.76
Pyrimethanil	-0.2243	-0.039	-5.5185909	2.640211	9.712381	0.865558	2.43
Pyrimidifen	-0.23385	-0.03454	0.41421541	5.395132	9.464438	0.687574	4.74
Pyrimisulfan	-0.27068	-0.04836	23.2943469	25.89798	7.619182	0.00117	1.2
Pyriofenone	-0.25288	-0.0747	12.2363415	14.03002	8.687298	0.33291	3.81
Pyriproxyfen	-0.2339	-0.0345	0.44527722	5.412331	9.46289	0.686642	4.75
Pyrithiobac-sodium	-0.25639	-0.04848	14.4168808	16.08021	8.502781	0.267494	0.53
Quinoxyfen	-0.25171	-0.06974	11.5094951	13.37829	8.745954	0.354715	4.98
Quizalofop	-0.25266	-0.0815	12.0996695	13.90626	8.698436	0.33701	0.39
Quizalofop-ethyl	-0.25326	-0.08156	12.4724113	14.2451	8.667941	0.325828	4.39
Quizalofop-P	-0.25268	-0.08144	12.1120942	13.91749	8.697426	0.336637	0.39
Quizalofop-P-ethyl	-0.25327	-0.0815	12.4786236	14.25078	8.667429	0.325641	4.39
Quizalofop-P-tefuryl	-0.25312	-0.08156	12.3854382	14.16567	8.67509	0.328437	4.45
Resorcinol	-0.24555	-0.01153	7.68267968	10.20807	9.031274	0.46952	1.36
Rhodamine 6G	-0.2192	-0.10173	-8.6868958	1.601073	9.805903	0.960607	6.71
Ronoxon	-0.27134	-0.04423	23.7043629	26.40848	7.573237	-0.01113	3.58
Rotenone	-0.22994	-0.07191	-2.0148184	4.139672	9.57743	0.760445	3.32
Salicylic acid	-0.26144	-0.064	17.5541239	19.27991	8.214809	0.173376	-1.52
Scarlet red	-0.2288	-0.10293	-2.7230277	3.806923	9.607377	0.781691	7.75
Sethoxydim	-0.2286	-0.05452	-2.847275	3.750095	9.612491	0.785418	2.35
Siduron	-0.23504	-0.01384	1.15348656	5.812325	9.426891	0.665395	3.27
Silafluofen	-0.2409	-0.02544	4.79393106	8.105653	9.220491	0.556182	7.61
Silthiofam	-0.23964	-0.03502	4.01117337	7.579031	9.267887	0.579665	3.95
Simetryn	-0.24586	-0.01788	7.87526292	10.35712	9.017859	0.463742	2.17
Sodium 1-amino-4-p-anisidino-9 10-dihydro-9 10-dioxoanthracene-2-sulphonate	-0.21273	-0.1244	-12.706294	0.715697	9.885587	1.081189	2.13
Sodium 2-mercaptobenzothiolate	-0.23232	-0.0528	-0.5362761	4.882807	9.510547	0.716088	2.63
Sodium 2-methoxy-5-nitrophenolate	-0.2547	-0.10465	13.3669915	15.0753	8.593223	0.29899	1.41
Sodium 6-((4-amino-3-bromo-9 10-dihydro-9 10-dioxo-1-anthryl)amino)toluene-3-sulphonate	-0.25091	-0.13406	11.012506	12.94177	8.78524	0.369625	3.57
Sodium dicamba	-0.27158	-0.04216	23.8534596	26.59537	7.556417	-0.0156	-0.83

Sodium salicylate	-0.26144	-0.064	17.5541239	19.27991	8.214809	0.173376	-1.52
Sodium tetrathiocarbamate	-0.25573	-0.08548	14.0068648	15.68383	8.538455	0.279794	-2.76
Solvent Black 3	-0.20232	-0.1123	-19.173364	0.307351	9.922338	1.275201	8.13
Solvent Blue 101	-0.20536	-0.11268	-17.284806	0.297032	9.923267	1.218544	4.48
Solvent Yellow 43	-0.22108	-0.09049	-7.5189717	1.94912	9.774579	0.925569	4.03
Solvent Yellow 72	-0.24244	-0.08506	5.7506349	8.77424	9.160318	0.527481	3.46
Sulfallate	-0.2296	-0.02961	-2.2260387	4.038858	9.586503	0.766781	3.1
Sulfaquinoxaline	-0.24086	-0.08043	4.76908161	8.088653	9.222021	0.556928	1.01
Sulfentrazone	-0.26735	-0.05659	21.2256302	23.39908	7.844083	0.063231	2.04
Sulfur dioxide	-0.36319	-0.15642	80.7649135	146.5922	-3.2433	-1.72295	0.32
Sulfur monochloride	-0.26999	-0.08264	22.8656939	25.36965	7.666731	0.014029	1.29
Sulisobenzone	-0.27311	-0.08944	24.8039511	27.80244	7.44778	-0.04412	-0.44
Sulprofos	-0.22687	-0.0297	-3.9220137	3.277849	9.654994	0.81766	5.02
SX purple	-0.24757	-0.11022	8.93757693	11.19929	8.942064	0.431873	-4.28
tau-Fluvalinate	-0.24434	-0.04358	6.9309838	9.636917	9.082677	0.49207	6.98
Tebuthiuron	-0.25466	-0.03894	13.342142	15.05191	8.595328	0.299736	1.35
Teflubenzuron	-0.26378	-0.06127	19.0078167	20.86254	8.072372	0.129765	4.49
Temephos	-0.23338	-0.03788	0.12223437	5.23487	9.478862	0.696333	5.54
Terbufos sulfone	-0.26857	-0.02877	21.9835384	24.29971	7.763026	0.040494	2.31
Terbutryn	-0.24508	-0.01675	7.39069863	9.984205	9.051422	0.478279	2.87
Tetrasul	-0.24588	-0.0438	7.88768764	10.36678	9.01699	0.463369	6.58
Thiabendazole	-0.24033	-0.05633	4.43982639	7.865145	9.242137	0.566805	2.33
Thiacloprid	-0.26342	-0.04431	18.7841717	20.61493	8.094656	0.136475	2.06
Thiamethoxam	-0.2687	-0.05996	22.0642991	24.39669	7.754298	0.038071	1.07
Thiamine	-0.26185	-0.06613	17.8088307	19.55263	8.190263	0.165735	-3.1
Thiamine hydrochloride	-0.26185	-0.06613	17.8088307	19.55263	8.190263	0.165735	-3.1
Thiazopyr	-0.25068	-0.07748	10.8696217	12.81765	8.796412	0.373911	4.62
Thidiazuron	-0.24285	-0.06092	6.00534177	8.956864	9.143882	0.51984	1.86
Thiencarbazone-methyl	-0.26657	-0.06427	20.7410659	22.83229	7.895094	0.077768	0.65
Thifensulfuron	-0.29035	-0.09761	35.5140642	43.275	6.05525	-0.36542	-5.69
Thifensulfuron methyl	-0.29285	-0.10068	37.0671549	45.8041	5.827631	-0.41201	0.99
Thiobencarb	-0.25263	-0.02756	12.0810324	13.88943	8.699951	0.337569	3.73
Thiocyclam	-0.22885	-0.04168	-2.6919659	3.821202	9.606092	0.780759	0.59
Thiodicarb	-0.24981	-0.02858	10.3291462	12.35365	8.838171	0.390126	2.47
Thiometon	-0.23466	-0.02334	0.91741678	5.677324	9.439041	0.672477	2.32
Thiophanate	-0.24167	-0.05091	5.27228298	8.436518	9.190713	0.541832	3.22
Thiophanate-methyl	-0.24207	-0.05175	5.52077749	8.611102	9.175001	0.534377	2.51
Thiram	-0.23071	-0.07537	-1.5364665	4.372929	9.556436	0.746094	2.73

Thymol	-0.24069	-0.00734	4.66347144	8.016608	9.228505	0.560096	3.43
Tiadinil	-0.25117	-0.08725	11.1740275	13.08283	8.772545	0.364779	0.23
Tiocarbazil	-0.25034	-0.02109	10.6584014	12.63527	8.812825	0.380248	5
Tolclofos-methyl	-0.2707	-0.034	23.3067717	25.91337	7.617796	0.000797	4.38
Tolfenpyrad	-0.24295	-0.04571	6.0674654	9.001701	9.139847	0.517976	4.57
Tolpyralate	-0.2664	-0.07409	20.6354557	22.7097	7.906127	0.080936	2.01
Tolylfluanid	-0.26778	-0.04673	21.4927618	23.71455	7.81569	0.055217	3.73
Tralomethrin	-0.2586	-0.07892	15.7898129	17.44418	8.380024	0.226306	6.82
trans-Permethrin	-0.25114	-0.02732	11.1553904	13.06651	8.774014	0.365338	5.7
Triadimefon	-0.25149	-0.05267	11.3728231	13.25751	8.756824	0.358815	3.97
Triadimenol	-0.2538	-0.02576	12.8078788	14.55361	8.640175	0.315764	3.28
Tri-allate	-0.25608	-0.04024	14.2242975	15.8934	8.519594	0.273271	3.8
Triasulfuron	-0.26528	-0.05914	19.9396711	21.91036	7.978068	0.10181	1.49
Triazamate	-0.25093	-0.03867	11.0249308	12.9526	8.784266	0.369252	2.61
Tribufos	-0.26123	-0.04572	17.4236643	19.14097	8.227313	0.17729	5.57
Trichloronat	-0.263	-0.04212	18.5232524	20.32796	8.120484	0.144302	4.77
Triflumuron	-0.25672	-0.06375	14.6218887	16.28029	8.484774	0.261343	4.75
Triphenyl phosphate	-0.27691	-0.02767	27.1646489	30.91752	7.167424	-0.11494	5.09
Triphenyl phosphite	-0.26917	-0.02513	22.3562802	24.74895	7.722594	0.029312	5.95
Triton X-100	-0.23453	-0.00939	0.83665607	5.631523	9.443163	0.6749	4.15
Vamidothion	-0.24558	-0.02053	7.70131676	10.22244	9.02998	0.46896	0.32
Vernolate	-0.25141	0.01051	11.3231242	13.21373	8.760764	0.360306	3.33
XMC	-0.26151	-0.01406	17.5976104	19.32633	8.21063	0.172072	2.5
zeta-Cypermethrin	-0.25493	-0.03861	13.5098758	15.21012	8.581089	0.294704	5.44
Ziram	-0.2316	-0.03092	-0.9835662	4.651081	9.531403	0.729507	0.15

[1] Energies calculated at the mPW1PW91/MIDIX+ level of theory using Gaussian 16 software
[2] Calculated from linear model[8] $\Delta G_{et}^0 = 22.83 \times \Delta E_{HOMO\text{-}SOMO} - 45.51$
[3] Calculated from linear model[8] $\Delta G^{\ddagger} = 7.81 \times (\Delta E_{HOMO\text{-}SOMO})^2 - 18.79 \times \Delta E_{HOMO\text{-}SOMO} + 11.59$
[4] Calculated from linear model[8] $\log k = -0.09 \times \Delta G^{\ddagger} + 9.95$
[5] Calculated from linear model[8] $E_{cell}^0 = -0.03 \times \Delta G_{et}^0 + 0.70$
[6] Calculated using ChemAxon's cxcalc plugin (Marvin v.6.0, 2013; ChemAxon)

Table S1. Computed and predicted data for pesticides utilized in current analysis.

Compound Name	***Functional Class***
(R)-Mecoprop	*Aryl ether*
1-Phenoxy-2-propanol	*Aryl ether*
2 4-D dimethylamine salt	*Aryl ether*
2 4-Dichlorophenoxyacetic acid	*Aryl ether*
2-((Dimethylamino)methyl)phenol	*Phenol*
2-(2-(2-(2-Phenoxyethoxy)ethoxy)ethoxy)ethanol	*Aryl ether*
2-(Butylthio)ethanol	*Sulfide*
2-Phenoxyethanol	*Aryl ether*
3-Hydroxycarbofuran	*Aryl ether*
4-Chlorophenoxyacetic acid	*Aryl ether*
4-Thiazolidinecarboxylic acid 3-acetyl-	*Sulfide*
Acephate	*Sulfide*
Acetic acid (2 4-dichlorophenoxy) with methanamine (1:1)	*Aryl ether*
Acetic acid (2 4-dichlorophenoxy) with N-ethylethanamine (1:1)	*Aryl ether*
Bendiocarb	*Aryl ether*
Biotin	*Sulfide*
Carbetamide	*Aniline*
Cartap	*Sulfide*
Demeton-S-methylsulphon	*Sulfide*
Denatonium benzoate	*Aniline*
Dicamba	*Aryl ether*
Dicamba-dimethylammonium	*Aryl ether*
Dichlorprop	*Aryl ether*
Dichlorprop-P	*Aryl ether*
Dimethenamide ESA	*Sulfide*
Dimethoate	*Sulfide*
Dioxacarb	*Aryl ether*
Disulfoton sulfone	*Sulfide*
Entsufon sodium	*Aryl ether*
Ethanethiol	*Thiol*
Fenamiphos sulfone	*Aryl ether*
Fenamiphos sulfoxide	*Aryl ether*
L-Cysteine	*Thiol*
Malaoxon	*Sulfide*
MCPA	*Aryl ether*
MCPA dimethylamine salt	*Aryl ether*
MCPB	*Aryl ether*
Methyl disulfide	*Sulfide*
Methylene bis(thiocyanate)	*Sulfide*
Omethoate	*Sulfide*

Oxamyl	*Sulfide*
Oxamyl oxime	*Sulfide*
Oxydemeton-methyl	*Sulfide*
Oxydisulfoton	*Sulfide*
Phenol	*Phenol*
Phorate sulfone	*Sulfide*
Phorate sulfoxide	*Sulfide*
Prothiocarb	*Sulfide*
Pyrimisulfan	*Aniline*
Resorcinol	*Phenol*
Sodium dicamba	*Aryl ether*
Vamidothion	*Sulfide*

Table S2. Pesticides within safer chemical space. Pesticides identified by means of an octanol-water distribution coefficient, log $D_{o/w}$ (values < 1.7) and an energy difference between the highest occupied and the lowest unoccupied molecular orbitals, ΔE (values > 6 eV).

Functional Class	*SMARTS Pattern*
Phenol	c1ccccc1[OX2H]
Aniline	c1ccccc1[NX3H2]
Aryl Ether	c1ccccc1[OX2H0]
Sulfide	[#16X2H0]
Thiol	[#16X2H]

Table S3. SMARTS patterns used to mine US EPA's CompTox Chemical Dashboard for PPRI-oxidizable cores.

**3-methoxyacetophenone**<br>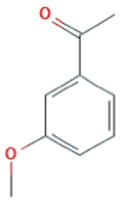	**2-Acetylnaphthalene**<br>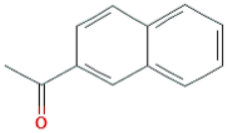	**Benzophenone**<br>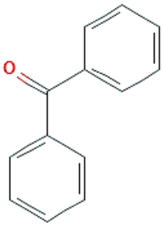	**4-carboxybenzophenone**<br>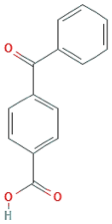
**9-Fluorenone**<br>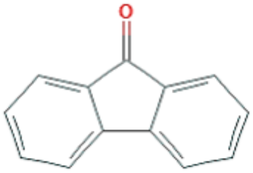	**Acetophenone**<br>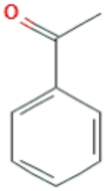	**Benzil**<br>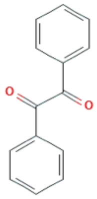	**Biacetyl**<br>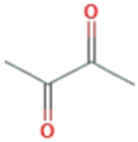
**2-Naphthaldehyde**<br>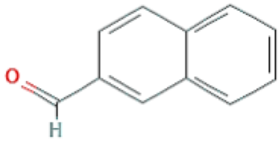	**Cinnamic acid**<br>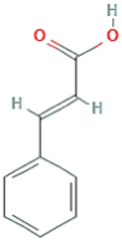	**Coumarin**<br>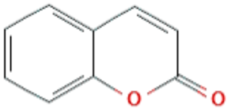	**Flavone**<br>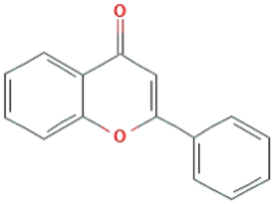
**Umbelliferone**<br>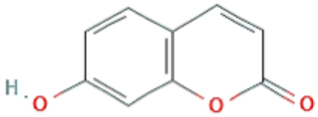	**Xanthone**<br>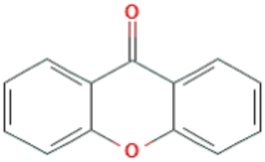	**Anthraquinone**<br>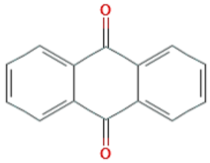	**Douroquinone**<br>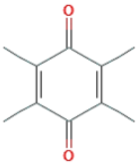
**Naphthoquinone**<br>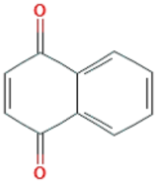	**Anthracene**<br>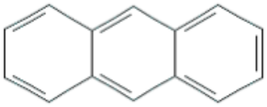	**Naphthalene**<br>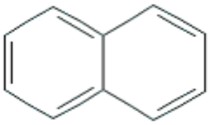	**Phenanthrene**<br>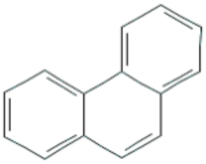
**Pyrene**<br>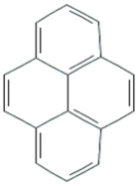	**Lumichrome**<br>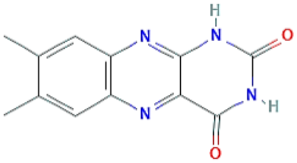	**Perinaphthenone**<br>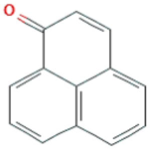	

Table S4. Structures of CDOM molecules used in this study. Sensitizing functional groups are color-coded, including ketones/aldehydes (red), coumarins/chromones (blue), quinones (orange), polyaromatic hydrocarbons (green), and other sensitizers (purple).

4-Cyanophenol 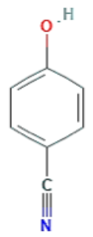	4-Nitrophenol 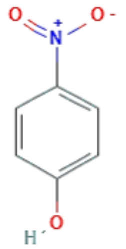	3-Nitrophenol 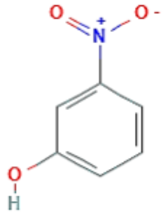	2-Nitrophenol 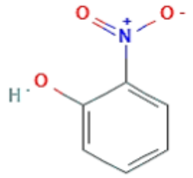
4-Hydroxybenzoic Acid 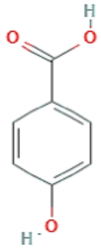	3-Hydroxybenzoic Acid 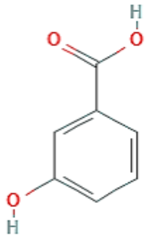	2-Hydroxybenzoic Acid 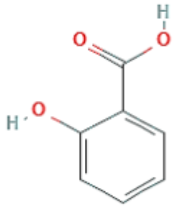	4-Nitroaniline 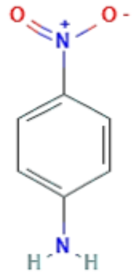
3-Nitroaniline 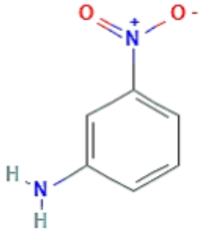	4-Aminobenzoic Acid 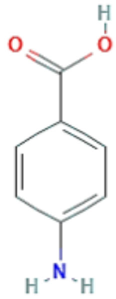	4-Chlorophenol 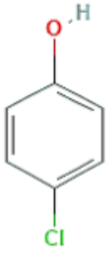	3-Chlorophenol 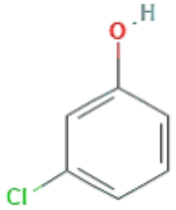
2-Chlorophenol 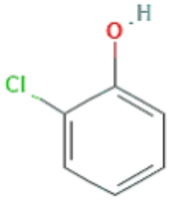	4-Methylphenol 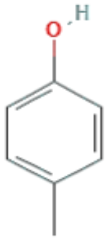	3-Methylphenol 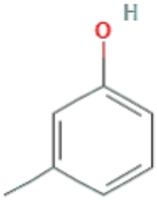	2-Methylphenol 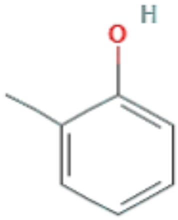
4-Ethylphenol 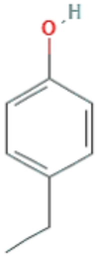	4-Chloroaniline 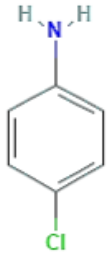	3-Chloroaniline 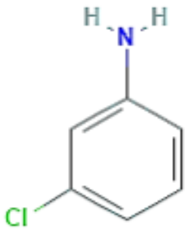	2-Chloroaniline 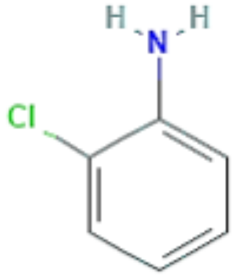

**4-Toluidine**<br>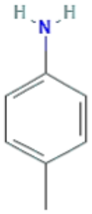	**3-Methylaniline**<br>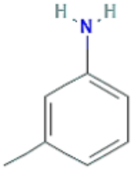	**2-Methylaniline**<br>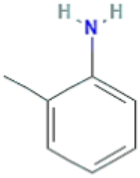	**2-Methyl-5-nitroaniline**<br>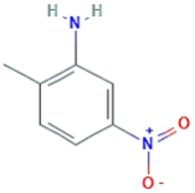
**4-Hydroxyphenol**<br>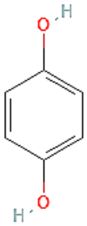	**3-Hydroxyphenol**<br>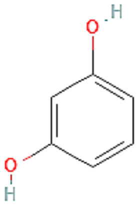	**2-Hydroxyphenol**<br>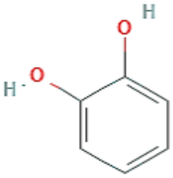	**4-Methoxyphenol**<br>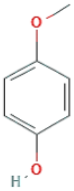
**3-Methoxyphenol**<br>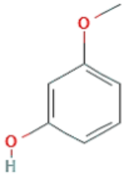	**2-Methoxyphenol**<br>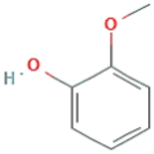	**4-Aminophenol**<br>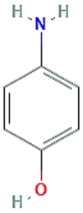	**3-Aminophenol**<br>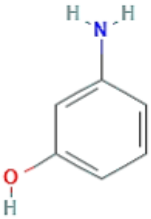
**3,4-Dimethoxyphenol**<br>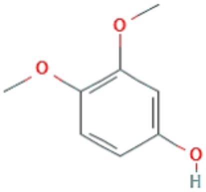	**2-Methoxy-4-formylphenol**<br>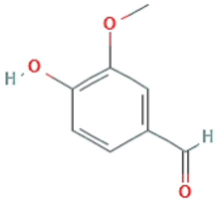	**2,6-Dimethoxyphenol**<br>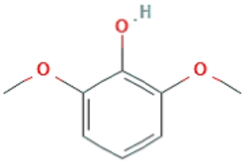	**4-Ethyl-2-methoxyphenol**<br>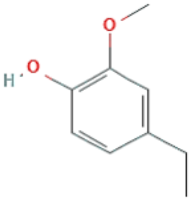
**2,4,6-Trimethylphenol**<br>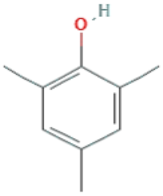	**2,5-Dimethylphenol**<br>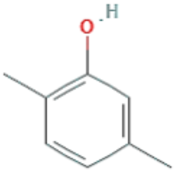	**4-Methoxyaniline**<br>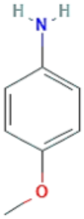	**3-Methoxyaniline**<br>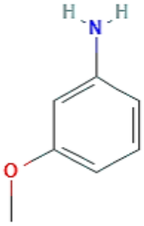
**2-Methoxyaniline**<br>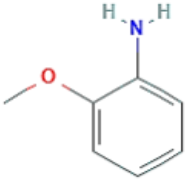	**4-Phenylenediamine**<br>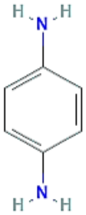	**4-Methyl-3-nitroaniline**<br>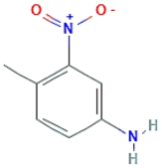	**2-Methoxy-5-nitroaniline**<br>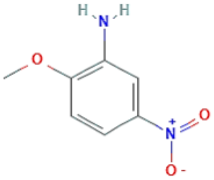

Table S5. Structures of oxidizable phenol and aniline pesticides used in Natural Bond Order (NBO) and Hirshfeld population analysis (HPA). Color labeling based on groups with combined strongly electron-withdrawing effect (orange), strongly electron-donating effect (green), and weakly withdrawing or donating effect (blue).

REFERENCES AND NOTES

1. P. Anastas, J. Warner, Green chemistry: Theory and practice (Oxford Univ. Press, 1998), pp. 29– 56.

2. A. Hamilton, Industrial poisons in the united states. *Nature* **116**, 604 (1925).

3. P. Coish, B.W. Brooks, E.P. Gallagher, T.J. Kavanagh, A. Voutchkova-Kostal, J.B. Zimmerman, P.T. Anastas, Current status and future challenges in molecular design for reduced hazard. *Sustainable Chem. Eng.* **4**, 5900–5906 (2016).

4. B. W. Brooks, T. Sabo-Attwood, K. Choi, S. Kim, J. Kostal, Carlie A. La Lone, L. M. Langan, L. Margiotta-Casaluci, J. You, X. Zhang, Toxicology advances for 21st century chemical pollution. *One Earth*, **2**, 312–316 (2020).

5. T. Clymer, V. Vargas, E. Corcoran, R. Kleinberg, J. Kostal, Redesigning hazardous chemicals by learning from structure-based drug discovery. *Green Chem.* **21**, 1935–1946 (2019).

6. National Research Council, Science for environmental protection: The road ahead (National Academies Press, 2012).

7. W. Jorgensen, The many roles of computation in drug discovery. *Science* **303**, 1813–1818 (2004).

8. J. Kostal, A. Voutchkova-Kostal, Going all in: A strategic investment in *in silico* toxicology. *Chem. Res. Toxicol.* **33**, 880–888 (2020).

9. R. Jayaraj, P. Megha, P. Sreedev, Organochlorine pesticides, their toxic effects on living organisms and their fate in the environment. *Interdiscip Toxicol.* **9**, 90–100 (2016).

10. W. J. Zhang, Global pesticide use: Profile, trend, cost/benefit and more. *Proceedings of the International Academy of Ecology and Environmental Sciences* **8**, 1–27 (2018).

11. K. Fenner, S. Canonica, L. P. Wackett, M. Elsner, Evaluating pesticide degradation in the environment: Blind spots and emerging opportunities. *Science* **341**, 752–758 (2013).

12. C. Damalas, I. Eleftherohorinos, Pesticide exposure, safety issues, and risk assessment indicators. *Int. J. Environ. Res. Public Health* **8**, 1402–1419 (2011).

13. C. K. Remucal, The role of indirect photochemical degradation in the environmental fate of pesticides: A review. *Environ Sci-Proc Imp* **16**, 628–653 (2014).

14. J. K. Challis, M. L. Hanson, K. J. Friesen, C. S. Wong, A critical assessment of the photodegradation of pharmaceuticals in aquatic environments: Defining our current understanding and identifying knowledge gaps. *Environ Sci-Proc Imp* **16**, 672–696 (2014).

15. S. W. Yan, W. H. Song, Photo-transformation of pharmaceutically active compounds in the aqueous environment: A review. *Environ Sci-Proc Imp* **16**, 697–720 (2014).

16. T. Zeng, W. A. Arnold, Pesticide photolysis in prairie potholes: Probing photosensitized processes. *Environ. Sci. Technol.* **47**, 6735–6745 (2013).

17. J. Lewer, J. Huang, J. Peloquin, J. Kostal, Structure-energetics-property relationships support computational design of photodegradable pesticides. *Environ. Sci. Technol.* 55, 11713–11722 (2021).

18. J. Kim, S.D. Shin, S. Jeong, G.J. Suh, Y.H. Kwak, Effect of prohibiting the use of Paraquat on pesticide-associated mortality. *BMC Public Health* **17**, 858 (2017).

19. R. L. Kellogg, R. F. Nehring, A. Grube, D. W. Goss, S. Plotkin, Environmental indicators of pesticide leaching and runoff from farm fields, in *Agricultural Productivity. Studies in Productivity and Efficiency*, V.E. Ball, G.W. Norton, Eds. (Springer, 2002).

20. M. May, W. Drost, S. Germer, T. Juffernholz, S. Hahn, Evaluation of acute-to-chronic ratios of fish and *Daphnia* to predict acceptable no-effect levels. *Environ Sci Eur* **28**, 16 (2016).

21. J. Kostal, A. Voutchkova-Kostal, P.T. Anastas, J.B. Zimmerman, Identifying and designing chemicals with minimal acute aquatic toxicity. *Proceedings Natl. Acad. Sci. U.S.A.* **112**, 6289–6294 (2015).

22. A. M. Voutchkova-Kostal, J. Kostal, K. A. Connors, B. W. Brooks, P. T. Anastas, J. B. Zimmerman, Towards rational molecular design for reduced chronic aquatic toxicity. *Green Chem.* **14**, 1001–1008 (2012).

23. G. Ankley, D. Villeneuve, The fathead minnow in aquatic toxicology: Past, present and future. *Elsevier* **78**, 91–102 (2006).

24. P. Coish, B. W. Brooks, E. P. Gallagher, M. Mills, T. J. Kavanagh, N. Simcox, G. A. Lasker, D. Botta, S. C. Schmuck, A. Voutchkova-Kostal, J. Kostal, M. L. Mullins, S. M. Nesmith, K. E. Mellor, J. Corrales, L. A. Kristofco, G. N. Saari, B. Steele, L. Q. Shen, F. Melnikov, J. B. Zimmerman, P. T. Anastas, The molecular design research network. *Society of Toxicology* **161**, 241–248 (2018).

25. A. T. Ford, M. Ågerstrand, B. W. Brooks, J. Allen, M. G. Bertram, T. Brodin, Z. C. Dang, S. Duquesne, R. Sahm, F. Hoffmann, H. Hollert, S. Jacob, N. Klüver, J. M. Lazorchak, M. Ledesma, S. D. Melvin, S. Mohr, S. Padilla, G. G. Pyle, S. Scholz, M. Saaristo, E. Smit, J. A. Steevens, S. van den Berg, W. Kloas, B. B. M. Wong, M. Ziegler, G. Maack, The role of behavioral ecotoxicology in environmental protection. *Environ. Sci. Technol.***55**, 5620–5628 (2021).

26. A. M. Voutchkova, J. Kostal, J. B. Steinfeld, J. W. Emerson, B. W. Brooks, P. Anastas, J. B. Zimmerman, Towards rational molecular design: Derivation of property guidelines for reduced acute aquatic toxicity. *Green Chem.* **13**, 2373–2379 (2011).

27. V. Lushchak, T.M. Matviishyn, V.V. Husak, J.M. Storey, K.B. Storey, Pesticide toxicity: A mechanistic approach. *EXCLI J.* **17**, 1101–1136 (2018).

28. R. M. Lopachin, T. Gavin, A. Decaprio, D. S. Barber, Application of the hard and soft, acids and bases (HSAB) theory to toxicant–target interactions. *Chem. Res. Toxicol.***25**, 239–251 (2012).

29. J. A. Pals, E. D. Wagner, M. J. Plewa, Energy of the lowest unoccupied molecular orbital, thiol reactivity, and toxicity of three monobrominated water disinfection byproducts. *Environ. Sci. Technol.* **50**, 3215–3221 (2016).

30. Richard M. Lo Pachin, T. Gavin, B. C. Geohagen, S. Das, Neurotoxic mechanisms of electrophilic type-2 alkenes: Soft–soft interactions described by quantum mechanical parameters. *Toxicol. Sci.* **98**, 561–570 (2007).

31. P. Griffin, S. Ramer, M. Winfough, J. Kostal, Practical guide to designing safer ionic liquids for cellulose dissolution using a tiered computational framework. *Green Chem.* **22**, 3626–3637 (2020).

32. C.M. Tice, Selecting the right compounds for screening: Does Lipinski's rule of 5 for pharmaceuticals apply to agrochemicals? *Pest Manag. Sci.***57**, 3–16 (2001).

33. K. Elokely, P. Velisetty, L. Delemotte, E. Palovcak, M.L. Klein, T. Rohacs, V. Carnevale, Understanding TRPV1 activation by ligands: Insights from the binding modes of capsaicin and resiniferatoxin. *Proceedings Natl. Acad. Sci. U.S.A.* **113**, E137–E145 (2016).

34. M. J. Caterina, M. A. Schumacher, M. Tominaga, T. A. Rosen, J. D. Levine, D. Julius, The capsaicin receptor: A heat-activated ion channel in the pain pathway. *Nature*, **389**, 816–824 (1997),

35. United States Environmental Protection Agency (2021, December 31) CompTox Chemicals Dashboard, https://comptox.epa.gov/dashboard/

36. M. J. Frisch, G. W. Trucks, H. B. Schlegel, G. E. Scuseria, M. A. Robb, J. R. Cheeseman, G. Scalmani, V. Barone, G. A. Petersson, H. Nakatsuji, X. Li, M. Caricato, A. V. Marenich, J. Bloino, B. G. Janesko, R. Gomperts, B. Mennucci, H. P. Hratchian, J. V. Ortiz, A. F. Izmaylov, J. L. Sonnenberg, D. Williams-Young, F. Ding, F. Lipparini, F. Egidi, J. Goings, B. Peng, A. Petrone, T. Henderson, D. Ranasinghe, V. G. Zakrzewski, J. Gao, N. Rega, G. Zheng, W. Liang, M. Hada, M. Ehara, K. Toyota, R. Fukuda, J. Hasegawa, M. Ishida, T. Nakajima, Y. Honda, O. Kitao, H. Nakai, T. Vreven, K. Throssell, J. A. Montgomery, Jr., J. E. Peralta, F. Ogliaro, M. J. Bearpark, J. J. Heyd, E. N. Brothers, K. N. Kudin, V. N. Staroverov, T. A. Keith, R. Kobayashi, J. Normand, K. Raghavachari, A. P. Rendell, J. C. Burant, S. S. Iyengar, J. Tomasi, M. Cossi, J. M. Millam, M. Klene, C. Adamo, R. Cammi, J. W. Ochterski, R. L. Martin, K. Morokuma, O. Farkas, J. B. Foresman, D. J. Fox, Gaussian 16, Revision B.01 (Gaussian Inc., 2016).

37. F. Weinhold, C. Landis, Natural bond orbitals and extensions of localized bonding concepts. *Chem. Educ. Res. Pract*. **2**, 91–104 (2001).

38. Natural Bond Orbital, https://nbo7.chem.wisc.edu/webnbo_css.htm [accessed 2 March 2022].

39. F. L. Hirshfeld, Bonded-atom fragments for describing molecular charge densities. *Theor. Chem. Acc.* **44**, 129–38 (1977).

40. R. K. Roy, K. Hirao, On non-negativity of Fukui function indices II. *J. Chem. Phys.* **113**, 1372–1379 (2000).

41. A. V. Marenich, S. V. Jerome, C. J. Cramer, D. G. Truhlar, Charge model 5: An extension of hirshfeld population analysis for the accurate description of molecular interactions in gaseous and condensed phases. *Journal of Chemical Theory and Computation* **8**, 527–541 (2012).

42. S. Saha, R. K. Roy, P. W. Ayers, Are the Hirshfeld and Mulliken population analysis schemes consistent with chemical intuition. *Journal of Quantum Chemistry* **109**, 1790–1806 (2009).

43. R. G. Parr, P. W. Ayers, R. F. Nalewajski, What is an atom in a molecule. *J. Phys. Chem.* **109**, 3957–3959 (2005).

44. P. W. Ayers, Atoms in molecules, an axiomatic approach. I. Maximum transferability. *J. Chem. Phys.* **113**, 10886 (2000).

45. R. G. Parr, R. F. Nalewajski, Information theory, atoms in molecules, and molecular similarity. *Proc. Natl. Acad. Sci. U.S.A.* **97**, 8879–8882 (2000).

46. P. W. Ayers, R. C. Morrison, R. K. Roy, Variational principles for describing chemical reactions: Condensed reactivity indices. *J. Chem. Phys.* **116**, 8731–8744 (2002).

47. R. F. Nalewajski, Hirshfeld analysis of molecular densities: Subsystem probabilities and charge sensitivities. *Phys. Chem. Chem. Phys.* **4**, 1710–1721 (2002).

48. P. W. Ayers, Information theory, the shape function, and the Hirshfeld atom. *Theor Chem Acc*. **115** 370–378 (2006).

49. R. K. Roy, K. Hirao, Mulliken population analysis based evaluation of condensed Fukui function indices using fractional molecular charge. *J. Chem. Phys.* **115**, 2901–2907 (2001).

50. R. K. Roy, S. Pal, K. Hirao, On non-negativity of Fukui function indices. *J. Chem. Phys.* **110**, 8236–8245 (1999).
